# Main chain selective polymer degradation: controlled by the wavelength and assembly†

**DOI:** 10.1039/d4sc02172j

**Published:** 2024-07-02

**Authors:** Phuong T. Do, Federica Sbordone, Henrik Kalmer, Anna Sokolova, Chao Zhang, Linh Duy Thai, Dmitri V. Golberg, Robert Chapman, Berwyck L. J. Poad, Hendrik Frisch

**Affiliations:** a School of Chemistry and Physics, Queensland University of Technology (QUT) 2 George Street Brisbane QLD 4000 Australia berwyck.poad@qut.edu.au h.frisch@qut.edu.au; b Centre for Materials Science, Queensland University of Technology (QUT) 2 George Street Brisbane QLD 4000 Australia; c Australian Centre for Neutron Scattering, Australian Nuclear Science and Technology Organisation (ANSTO) New Illawarra Road, Lucas Heights NSW 2234 Australia; d Central Analytical Research Facility, Queensland University of Technology (QUT) 2 George Street Brisbane QLD 4000 Australia; e Centre for Advanced Macromolecular Design, School of Chemistry, UNSW Sydney Kensington NSW 2052 Australia; f School of Environmental and Life Sciences, University of Newcastle Callaghan NSW 2308 Australia

## Abstract

The advent of reversible deactivation radical polymerization (RDRP) revolutionized polymer chemistry and paved the way for accessing synthetic polymers with controlled sequences based on vinylic monomers. An inherent limitation of vinylic polymers stems from their all-carbon backbone, which limits both function and degradability. Herein, we report a synthetic strategy utilizing radical ring-opening polymerization (rROP) of complementary photoreactive cyclic monomers in combination with RDRP to embed photoresponsive functionality into desired blocks of polyvinyl polymers. Exploiting different absorbances of photoreactive cyclic monomers, it becomes possible to degrade blocks selectively by irradiation with either UVB or UVA light. Translating such primary structures of polymer sequences into higher order assemblies, the hydrophobicity of the photodegradable monomers allowed for the formation of micelles in water. Upon exposure to light, the nondegradable blocks detached yielding a significant reduction in the micelle hydrodynamic diameter. As a result of the self-assembled micellar environment, telechelic oligomers with photoreactive termini (*e.g.*, coumarin or styrylpyrene) resulting from the photodegradation of polymers in water underwent intermolecular photocycloaddition to photopolymerize, which usually only occurs efficiently at longer wavelengths and a much higher concentration of photoresponsive groups. The reported main chain polymer degradation is thus controlled by the irradiation wavelength and the assembly of the polymers.

## Abstract 2

A pair of cyclic monomers enables selective photocleavage at different wavelengths and self-assembly into photoreactive micelles, which alter depolymerization products.

## Graphical abstract

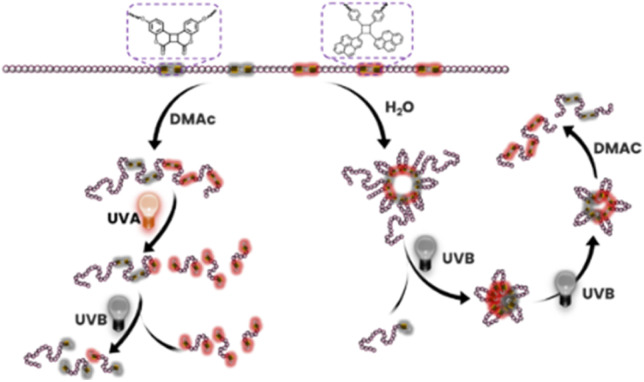

## Introduction

From vision to photosynthesis, nature's interactions with light stem from the precisely arranged macromolecular architectures of proteins.^1,2^ These proteins either enable photoreactions or are influenced by photoreactions. The underpinning macromolecular architectures derive their complex structures by translating defined biopolymer sequences—primary structures—into higher-order structures. The defining feature of these dynamic, stimuli-responsive structures is the hydrogen bonding of their polymer main chain, which is precisely arranged by the sequence of amino acid side chains. The advancement of reversible deactivation radical polymerization (RDRP) has empowered chemists to gain control over the sequences of vinyl polymers and expanded the possibilities within this widely used class of polymers.^3^

A major challenge, however, results from the non-functional carbon backbone of these polymers, which confines dynamic and stimuli-responsive interactions to the functional side chains or chain ends of polymers. Strategies to deconstruct vinyl polymers on demand with light thus traditionally rely on the translation of chain end or side chain functionalities into the main chains, such as the β-scission of polyvinyl ketones that enabled the design of light degradable polymers.^4–7^ Sumerlin and coworkers have developed elegant avenues to photochemically decarboxylate polyacrylates to induce similar radical main chain scission.^8^ Recent emerging avenues towards light controlled depolymerization guided by polymer chain ends are based on RDRP depolymerization.^9,10^ To embed functional groups directly into the main chain of vinyl polymers, the recent renaissance of radical ring-opening polymerization^11,12^ has enabled the incorporation of degradation targets such as esters,^13,14^ thioesters,^15,16^ disulfides,^17^ or amides^18^ based on thiolactones,^19^ cyclic ketal esters,^20^ cyclic allylic sulfides^18,21–23^ and sulfones^24,25^ as well as elegant ring-opening and closing mechanisms.^26–28^ These fundamental advancements paved the way for hydrolytic degradation of polymers^29^ on tunable timescales^30^ and gave rise to degradable networks^31^ and particles,^32^ latexes,^33^ and 3D printed materials^34^ and even enabled repolymerization.^35^

**Scheme 1:**
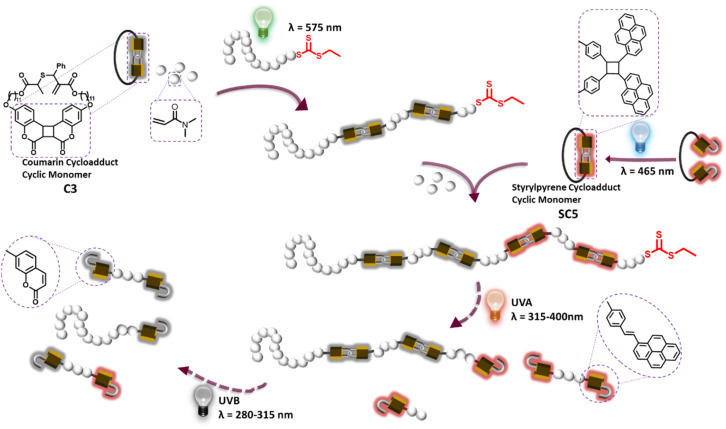
Triblock copolymer synthesis and its stepwise photodegradation: a diblock copolymer consisting of a PDMA nondegradable block and photodegradable copolymer of the coumarin cycloadduct and DMA block is prepared by green light-initiated RAFT polymerization. Chain-extension of this polymer with RAFT co-polymerization of DMA and the cyclic monomer resulted from intramolecular [2 + 2] cycloaddition of styrylpyrene under blue light yields a triblock polymer with the copolymer of DMA and styrylpyrene cycloadduct as the third block. Under UVA, the styrylpyrene cycloadduct experiences [2 + 2] cycloreversion, leading to the fragmentation of the third block. Subsequent UVB irradiation initiates the degradation of the second block as the coumarin dimer in the polymer backbone undergoes [2 + 2] cycloreversion.

To exploit the spatiotemporal control of light gated reactions to steer polymer main chain transformations, we have recently designed an allylic sulfide containing coumarin photocycloadduct, which provided access to photodegradable vinyl polymers.^36^ In addition to spatiotemporal reaction control of photoreactions, different molecules can absorb light of different wavelengths, offering additional energetic control over reactions: specific photon energies can initiate selective reactions.^37–40^ Here we report the main chain selective degradation of vinylic block-copolymers, where specific blocks are on-demand degraded into oligomers in response to different wavelengths of light (Scheme 1). Leveraging the difference in hydrophilicity between photodegradable and non-photodegradable monomers, the self-assembly of photoreactive block copolymers and its effect of the photodegradation is explored.

## Results and discussion

### Development and polymerization of a complementary photoreactive monomer

**Fig. 1:**
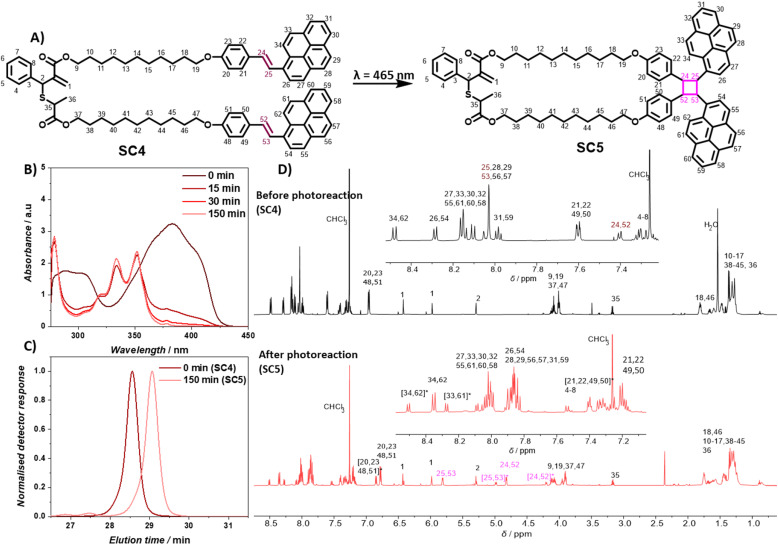
Transformation from linear monomer SC4 to cyclic monomer SC5: (A) reaction; (B) absorption spectrum of reaction solution under *λ* = 465 nm irradiation (0.05 mg mL^−1^ in THF); (C) SEC chromatogram of reaction solution (0.05 mg mL^−1^ in THF) before photoreaction and after 150 min of *λ* = 465 nm irradiation; (D) ^1^H NMR of the monomer before and after irradiation showing the appearance of cyclobutane proton resonances after the irradiation.

To enable selective polymer degradation, two monomers are required, which initiate degradation at different wavelengths. The previously developed coumarin based monomer C3 underwent photocycloreversion under UVB light irradiation. The photocycloreversion of cyclobutanes is largely controlled by the absorbance of the adjacent chromophores. We proposed that the stilbene derivative styrylpyrene^41–43^ would provide cyclobutanes with a suitable reactivity window to initiate photocycloreversion at longer wavelengths. To obtain a cyclic monomer for rROP, a telechelic styrylpyrene terminated allyl sulfide SC4 was synthesized (see Scheme S1† for synthetic details). The intramolecular [2 + 2] cycloaddition reaction of styrylpyrene end groups under blue light LED irradiation at dilute concentrations in toluene yielded the cyclic allyl sulfide SC5 (0.5 mg mL^−1^, *λ* = 465 nm, 10 W, and 1.05 A, Fig. 1A). The characteristic styrylpyrene absorbance maxima at *λ* = 383 nm decreased with irradiation time, approaching zero after 150 min of irradiation, suggesting a complete reaction (Fig. 1B). Meanwhile, two characteristic absorbance maxima (*λ* = 352 nm and 334 nm) of the styrylpyrene photocycloadduct increased over the course of reaction and an isosbestic point at *λ* = 355 nm was observed, indicating a selective photocycloaddition reaction.^42,44^ The photocycloaddition was further evidenced by the appearance of two pairs of distinct unresolved second-order multiplet resonances (*δ* = 5.81/4.82 ppm and 4.99/4.20 ppm) in the ^1^H NMR, attributed to the cyclobutane rings of two photocycloadduct isomers (Fig. 1D, full assignment of the ^1^H NMR and COSY NMR of SC5 is given in ESI, Fig. S20 and S21†).^42^ The SEC traces of linear SC4 and SC5 after photodimerization were acquired with a UV detector set to the isosbestic point of the [2 + 2] photocycloaddition and revealed that the product has a smaller pervaded volume compared to the starting material, as evidenced by later elution times (Fig. 1C). In addition, the mass of the product measured with a high resolution mass spectrometer (HR-MS) was found to align with the masses of SC4/SC5 (calculated for [SC4 + Na]^+^ = [SC5 + Na]^+^*m*/*z* 1233.6038, found in the starting material *m*/*z* 1233.6039, and found in the product *m*/*z* 1233.6029). These results indicate that the intramolecular [2 + 2] cycloaddition of the linear monomer SC4 occurred and afforded the desired cyclic monomer SC5 in quantitative yields.

**Fig. 2:**
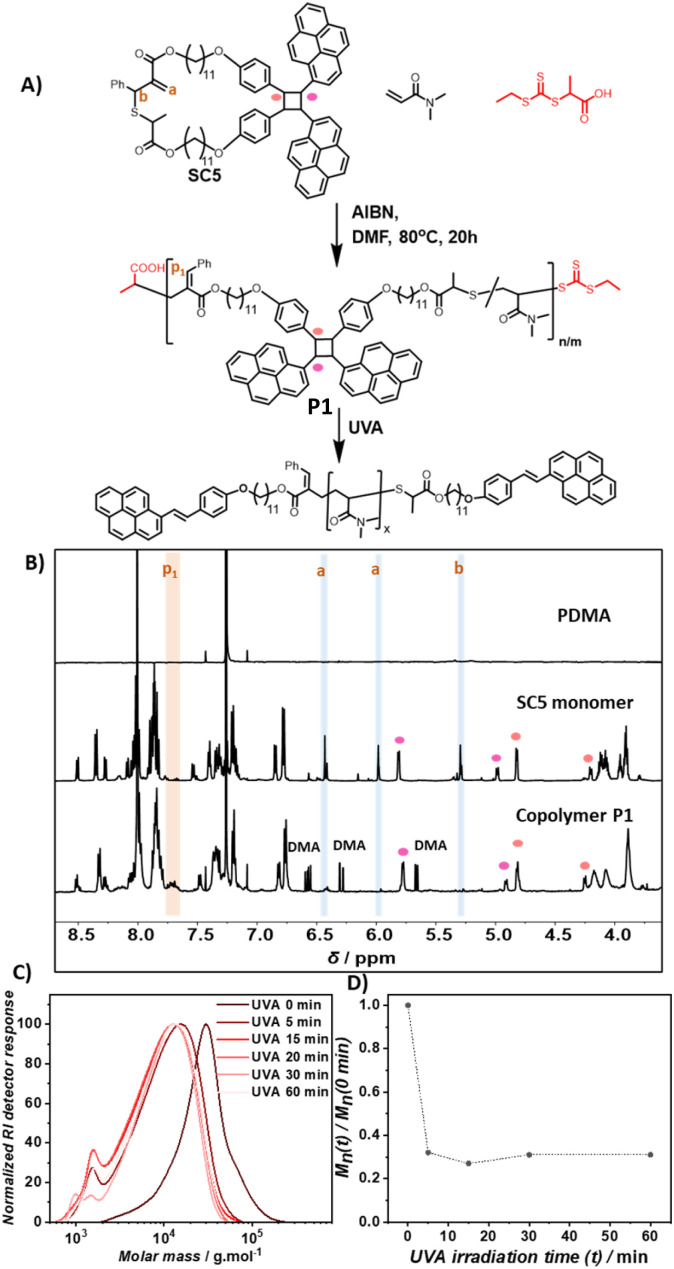
(A) Copolymerization of styrylpyrene based monomer SC5 and DMA, and photodegradation of the obtained copolymer P1 under UVA irradiation. Protons whose resonances change in the ^1^H NMR are denoted with orange letters; (B) ^1^H NMR spectra of PDMA, cyclic monomer SC5 and copolymer P1 P(DMA-*co*-SC5); (C) SEC traces of P1 in DMAc after UVA irradiation; (D) degradation upon UVA irradiation over time: reduction of the average molecular weight (*M*_n_) of P1 represented by the ratio of *M*_n_ under *t* min UVA irradiation to the *M*_n_ of pristine P1 plotted *vs.* UVA irradiation time.

Prior to the block copolymer synthesis, the ability of SC5 to copolymerize with DMA and the photodegradation behavior of the resulting copolymer were investigated. The polymerization of DMA and SC5 ([SC5] = 2%) in DMF with 2-(((ethylthio)carbonothioyl)thio)propanoic acid as the chain transfer agent ([DMA]/[CTA] = 500) yielded copolymer P(DMA-*co*-SC5) P1 (*M*_n_ = 20 kg mol^−1^ and *Đ* = 1.6, Fig. 2A). The ^1^H NMR spectra of control PDMA, cyclic monomer SC5, and copolymer P1 display the resonances of PDMA in both the homopolymer and the copolymer with the resonance at *δ* = 2.90 ppm attributable to the methyl protons of PDMA (Fig. S1†).^45^ In the ^1^H NMR spectrum of copolymer P1, the resonances of two double bond protons a and adjacent proton b of cyclic monomer SC5 deplete (*δ*: a = 6.43 ppm and 5.98 ppm, and b = 5.30 ppm, Fig. 2B). Meanwhile, a new resonance p1 of the resulting vinylarene proton *δ* = 7.70 ppm arises in copolymer P1, confirming the ring-opening polymerization of the cyclic monomer.^18,36^ The incorporation ratio of the styrylpyrene monomer and DMA determined from the integral of resonances p_1_ and the methyl proton of DMA was 1.8%, slightly lower than the feed ratio. Monitoring the copolymerisation of SC5 and DMA revealed that while the conversion of both monomers increased with polymerisation time, the conversion of SC5 remains slightly below DMA over the entire polymerisation time (Fig. S14†). The incorporation of the photodegradable monomers is thus expected to be largely random with only a slight gradient, yielding a marginally lower incorporation of the degradable monomer compared to the feed ratio.

Since UVB light effectively induced the photocycloreversion of coumarin derived cyclobutanes,^36^ the use of longer wavelengths is paramount for wavelength selective polymer degradation. The photodegradation of copolymer P1 was thus studied using longer wavelength UVA lamps (*λ* = 315–400 nm) for 60 min. After 5 min of UVA irradiation in DMAc (1 mg mL^−1^), the SEC trace of P1 shifted significantly to a lower molecular weight and the *M*_n_ decreased by approximately 65% (Fig. 2C and D). After 15 min, the SEC trace shifted only slightly further toward lower molecular weights with prolonged UVA irradiation and the molecular weight reduction reached a plateau at 70%. This suggests that the photodegradation is largely complete after 15 min of irradiation. The obtained reduction in *M*_n_ aligns well with the expected maximum reduction of 75% based on the cleavage of all degradable monomers (see Section 6.1.a in the ESI† for detailed estimation), indicating efficient cleavage of the embedded degradable monomers within P1.

To elucidate the photodegradation mechanism, SEC coupled to HR-MS was employed to analyze the degraded polymer solution. The acquired mass spectrum at an elution time of 19.0 min shows the *m*/*z* pattern of doubly charged polymer ions (Fig. S2†). The spacing between neighboring *m*/*z* peaks is equal to half of the DMA molar mass, indicating that these are DMA oligomer ions. The simulated data for *m*/*z* of DMA oligomers (15–30 repeat units) with telechelic styrylpyrene end groups and actual *m*/*z* are in good agreement, with mass deviation less than 5 ppm and all isotopic patterns of ions matching (Table S1 and Fig. S3†). These results indicate that clean photodegradation of the main chain of copolymer P1 occurred under UVA light *via* photocycloreversion of styrylpyrene cycloadducts.

### Diblock copolymer PDMA – P(coumarin-*co*-DMA)

**Fig. 3:**
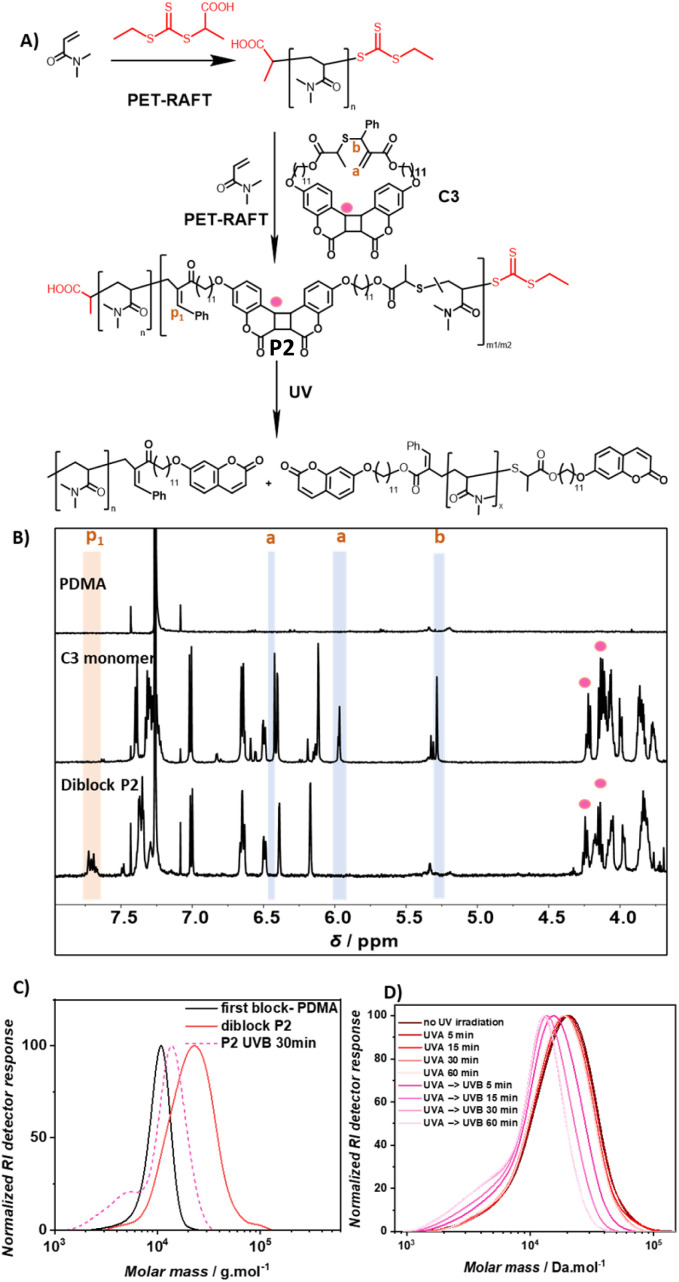
(A) Synthesis of diblock copolymer P2, PDMA-*b*-(DMA-*co*-C3), of coumarin based monomer C3 and DMA, and its photodegradation under UV light. Protons whose resonances change in the ^1^H NMR are denoted with orange letters; (B) ^1^H NMR spectra of PDMA, cyclic monomer C3 and copolymer P2 PDMA-*b*-(DMA-*co*-C3); (C) overlayed SEC traces of the first PDMA block, diblock copolymer P2 and UVB degraded P2 in DMAc; (D) SEC traces of polymer P2 in DMAc solution over the course of UVA and UVB irradiation.

To generate selectively photodegradable polymers, a photoresponsive diblock copolymer was synthesized by chain extension *via* photoinitiated electron/energy transfer addition–fragmentation chain transfer (PET-RAFT) polymerization (Fig. 3A).^46–48^ PET-RAFT polymerization has proven to be an effective method for block polymer synthesis and also enables the polymerization of low volume solutions in the presence of oxygen.^49,50^ To exclude undesired energy transfer between the photoinitiator and the photoreactive polymer main chains, ZnhTPP was utilized instead of previously reported photocatalysts for rROP utilizing blue light.^49,51^ The polymerization of DMA using 2-(((ethylthio)carbonothioyl)thio)propanoic acid as the chain transfer agent ([DMA]/[CTA] = 100) and ZnhTPP as the photocatalyst yielded a PDMA homopolymer (*M*_n_ = 9.6 kg mol^−1^ and *Đ* = 1.1). The subsequent copolymerization of coumarin based C3 and DMA ([C3]/[DMA] = 2/50) using the obtained PDMA as a macro-CTA ([DMA]/[CTA] = 50/1) resulted in copolymer P2 with an increased molecular weight (*M*_n_ = 18.5 kg mol^−1^) and narrow dispersity (*Đ* = 1.3). The ^1^H NMR of copolymer P2 revealed an incorporation ratio of 1% C3 (Fig. S26†) based on the resonance of PDMA methyl protons (*δ* = 2.90 ppm, Fig. S4†) and the resonance of vinylarene proton p_1_ (*δ* = 7.70 ppm, Fig. 3B). Diffusion-ordered spectroscopy (DOSY) NMR yielded compare diffusion coefficients of the resonances of coumarin protons (*δ* = 7.4, 7.0, 6.7, 6.5, 6.4, 6.2, 4.2, 4.1 ppm) and DMA protons (*δ* = 3.2, 2.9, 1.7 ppm) of P2 (Fig. S4, Appendix DOSY in the ESI†), confirming the successful copolymerization of C3 and DMA and the chain-extension of the pure PDMA block.

To test if C3 containing polymer blocks are sufficiently stable under UVA irradiation (*λ* = 315–400 nm), which is the target wavelength for the selective degradation of SC5 containing polymer blocks, P2 was irradiated with that wavelength. The degradation of P2 in DMAc was observed to be negligible as the SEC trace barely changed after 60 min of irradiation (Fig. 3D). In contrast, irradiation with UVB light resulted in a significant shift of the SEC trace to lower molecular weights within 5 min, indicative of effective polymer degradation that was complete after 30 min.

Overlaying the SEC traces of P2 after 30 min of UVB irradiation with unirradiated P2 and the first PDMA block shows a shift of the main polymer distribution to lower molecular weight ranges compared to diblock P2, yet higher than that of the first PDMA block (Fig. 3C). In addition, a shoulder can be observed at even lower molecular weights compared to the first block. The analysis of this low molecular weight shoulder (an elution time of 20.45 min) *via* SEC-MS displayed the characteristic *m*/*z* pattern of doubly charged coumarin-terminated DMA oligomer ions (Fig. S5, S6 and Table S2†). This observation indicates the successful degradation of the second photodegradable block, yielding degradation products of the first block (chain extended to the first coumarin monomer) and lower molecular weight fragments of the degraded second block. Since the photodegradation of specific polymer blocks yields two distinct polymer distributions, the *M*_p_ value was used to describe the efficiency of the block selective degradation. Based on its *M*_p_ and the incorporation ratio of C3 in P2, it is estimated that P2 (*M*_p_ = 23 kg mol^−1^) consists of one block of PDMA homopolymer (*M*_p_ = 11 kg mol^−1^ and DP = 111) and one block of copolymer of DMA and coumarin dimer monomer C3 (estimated DP_DMA_ = 103 and DP_C3_ = 2, Section 6.2.a of the ESI† details the estimation). The full photodegradation of C3 within P2 would result in the reduction of *M*_p_ to around 15 kg mol^−1^ (Section 6.2.b for detailed calculation), which aligns with the recorded *M*_p_ of 14 kg mol^−1^ obtained after irradiating P2 with UVB for 30 min. It can be concluded that the cycloreversion of the coumarin cycloadduct occurred effectively to disintegrate the polymer backbone of the photodegradable polymer block.

It is noteworthy that the presence of photodegradable comonomers affects the intrinsic physical properties of the PDMA polymer. Single block copolymers with different incorporation ratios of photodegradable monomer C3 to DMA were synthesized and subjected to glass transition (*T*_g_) measurement *via* DSC (Section 5.2-copolymer synthesis, ESI, Fig S13A and B†). The results show that the introduction of C3 into PDMA polymer chains leads to a significant decrease of *T*_g_ from 71 °C (pure PDMA) to 61 °C (P(DMA–2% C3)) (Fig. S13C†). The *T*_g_ of the copolymer reduced further to 44 °C and 35 °C as the incorporation ratios of C3 increased to 4% and 9%, respectively (Fig. S13C†). Moreover, higher incorporation ratios of the cyclic monomer yield an increase in dispersity as their propagating sulphur radical cannot be reversibly deactivated.

### Photo-depolymerization as a function of wavelength

**Scheme 2:**
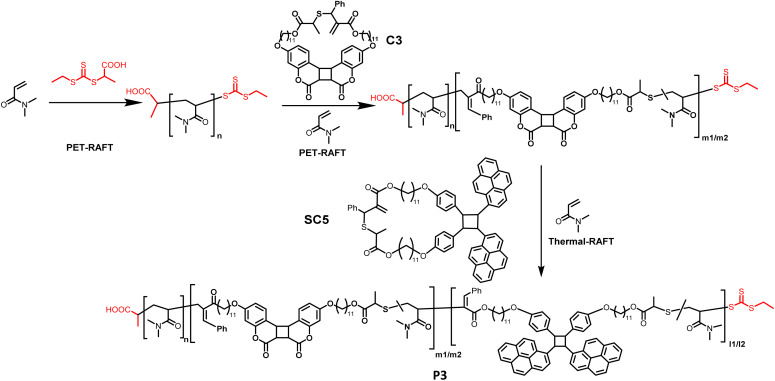
Chain-extension of PDMA with coumarin cyclic monomer C3 and DMA, followed by chain-extension with styrylpyrene cyclic monomer SC5 and DMA, *via* RAFT polymerization gives rise to triblock copolymer P3.

**Fig. 4:**
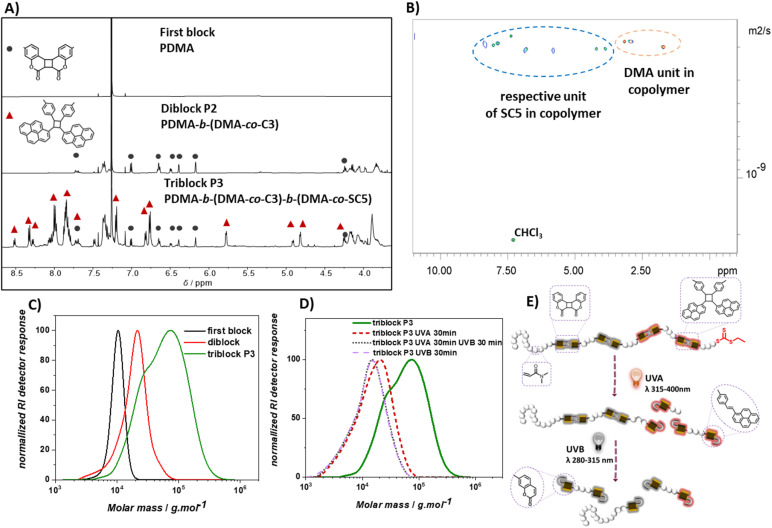
(A) Stacked ^1^H NMR spectra of the homopolymer PDMA, diblock copolymer PDMA-*b*-(DMA-*co*-C3) and triblock copolymer P3 PDMA-*b*-(DMA-*co*-C3)-*b*-(DMA-*co*-SC5); (B) DOSY NMR of polymer P3 showing similar diffusion coefficients for SC5 and DMA resonances, indicative of successful chain extension and copolymerization; (C) overlayed SEC traces of the first block PDMA, diblock polymer and triblock polymer P3; (D) overlayed SEC traces of triblock polymer P3, UVA degraded P3, and UVA–UVB degraded P3; (E) schematic illustration for stepwise UV-degradation of P3 corresponding to photolabile functional groups in each block of the polymer.

To gain access to polymers with wavelength selective degradability, the chain-extension of diblock polymers into a triblock polymer was carried out using thermal RAFT polymerization, due to the insolubility of SC5 in DMSO (Scheme 2). A diblock polymer (*M*_n_ = 16 kg mol^−1^ and *Đ* = 1.3) prepared by PET-RAFT was used as a macro-CTA and DMA and cyclic monomer SC5 and AIBN were introduced at ratios [DMA]/[CTA] = 500/1, [SC5]/[DMA] = 2/100, and [AIBN]/[CTA] = 1/10. Polymerization (80 °C and 22 h) afforded polymer P3 (*M*_n_ = 43 kg mol^−1^ and *Đ* = 1.8). The SEC trace of P3 shifted to a higher mass range compared to the diblock polymer precursor (Fig. 4C), suggesting successful chain-extension of the polymer; however, a shoulder at lower molecular weights was observed. RAFT copolymerization of cyclic allylsulfides has a propensity to yield chains without CTA, as the sulfur centered radical itself cannot directly be reversibly deactivated, but only after the addition of a DMA comonomer. As SC5 is the only monomer within P3 that absorbs light at *λ* = 360 nm (as shown in Fig. 1B),^36,52^ the SEC-UV trace of P3 at *λ* = 360 nm was used to investigate the origin of the shoulder of P3. The SEC-UV trace of P3 thus only represents the chain-extension product, *i.e.* the triblock polymer, which did not display a shoulder. The distribution of the unreacted diblock was estimated by subtraction of the normalized UV trace from the normalized RI trace (Fig. S8†). According to the integrated peak area, the non-reinitiated diblock accounts for <5% in relation to the triblock polymer P3 (Fig. S8†). The observed increased dispersity of P3 is thus a result of the third block copolymerization, and the shoulder, however, results from the second block. In the ^1^H NMR of P3, the resonances of the coumarin photocycloadduct, styrylpyrene photocycloadduct and DMA were observed (Fig. 4A and S8†). The incorporation ratio of coumarin and styrylpyrene in P3 was found to be 0.4% and 0.9%, respectively (refer to Section 6.2 in the ESI† for detailed calculation). In the DOSY NMR measurement of P3, only the diffusion coefficients of the styrylpyrene photocycloadduct and DMA are represented, as the coumarin photocycloadduct resonances were too weak to acquire reliable diffusion coefficients (Fig. 4B). As depicted in Fig. 4B, the diffusion coefficients of the styrylpyrene photocycloadduct (*δ* = 8.4, 8.3, 8.0, 7.9, 7.4, 6.9, 6.8, 5.8, 4.2, 3.8 ppm) and DMA (*δ* = 3.2, 2.9, 1.7 ppm) align, indicating that SC5 and DMA reside in the same polymer chains. Given the confirmation of the diblock polymer structure above in which the coumarin photocycloadducts are embedded into one block of the PDMA polymer chain and that the coumarin photocycloadduct resonances are clearly present in the ^1^H NMR of P3, it can be concluded that the chain extension of the diblock polymer with the styrylpyrene monomer and DMA was successful. If P3 was a mixture of only the copolymer of SC5 and DMA and diblock polymer, the DOSY NMR of P3 should have shown two different diffusion coefficients for DMA.

The investigation of the photodegradation behavior of P1 and P2 showed that the copolymer of DMA and SC5 degrades under UVA light in minutes, whereas polymers with the hypsochromically absorbing C3 coumarin cycloadducts in the main chain are sufficiently stable under UVA light and only rapidly degrade under UVB irradiation. Therefore, UVA and UVB light was employed in sequence to induce the selective photocycloreversion of SC5 and C3 respectively, causing stepwise degradation of triblock polymer P3 (Fig. 4E). When P3 in DMAc (1 mg mL^−1^) was exposed to UVA for 30 min, the styrylpyrene photocycloadduct in the third block of P3 was cleaved, resulting in a substantial shift in the SEC trace to a lower molar mass range, and the *M*_p_ reduced from 78 kg mol^−1^ to 21 kg mol^−1^ (Fig. 4D). Continuing to irradiate the polymer solution with UVB for 30 min further decreased the *M*_p_ of the polymer to 14 kg mol^−1^, as the second block of P3 degraded due to the dissociation of the coumarin based photocycloadducts, matching the expected *M*_p_ of 14 kg mol^−1^ for P3 fully degraded (Section 6.3 in the ESI† for detailed calculations). These results showcase the possibility to selectively and effectively degrade specific sections of polymer main chains using degradable rROP monomers. Such wavelength selective degradation of polymer materials has traditionally been limited to gels and networks, where different photoreactive moieties are used as crosslinkers.^53,54^ To initiate the simultaneous cleavage of both photoresponsive groups in the triblock polymer P3, the degradation of the polymer was conducted under UVB light irradiation. The SEC chromatogram of the UVB degraded polymer resembles that of the stepwise UVA–UVB degraded polymer (Fig. 4D), indicating that both the coumarin cycloadduct and styrylpyrene cycloadduct degraded concomitantly under those irradiation conditions.

### Higher order structure control over photochemistry

To examine the effect of self-assembly on the main chain selective photoreactivity, diblock copolymer P2 was dispersed in water (1.5 mg mL^−1^) and irradiated with UVB light. Dispersions of P2 in water after 0 min, 30 min and 90 min of UVB irradiation were analyzed by small-angle neutron scattering (SANS). Initial model-independent analysis using the Guinier approximation revealed a reduced radius of gyration (*R*_g_) of close to 60% upon 30 min of irradiation (Table 1, ESI, Section 7†), consistent with DLS results (Fig. S10†).

**Table 1:** Estimated values of *R*_g_ and diameter (*D*) of P2^a^

UVB irradiation/min	*R* ^Guinier^_g_/nm	*R*_g_^(*p*(*r*))^/nm	*D*, from *R*_g_^(*p*(*r*))^/nm	*D*_max_^(*p*(*r*))^/nm
0	33	36	∼100	120–140
30	12	9	∼25–30	40–50
90	15	15	∼40	50–60

a *D*_max_ is very sensitive to the choice of the lowest value of *q*, and the solution is not always stable; hence a feasible range is given for the *D*_max_.

**Fig. 5:**
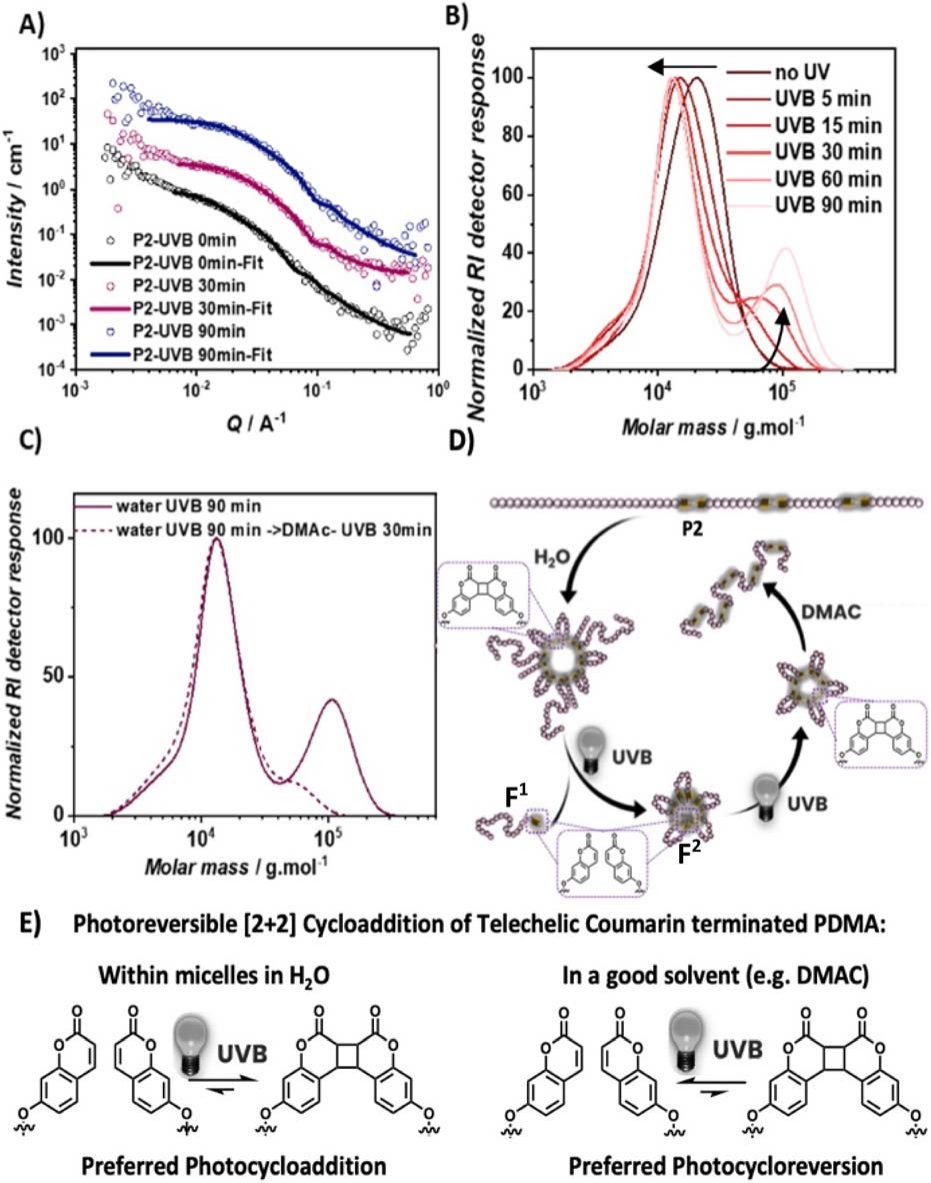
A dispersion of diblock copolymer P2 in water (1.5 mg mL^−1^) was irradiated with UVB for 90 min. (A) Experimental SANS data (scattering intensity plotted *versus* momentum transfer *q*) of P2 dispersion in water irradiated with UVB light for 0 min, 30 min and 90 min and their fit with a spherical polymer micelle model *via* SasView. Note that the data and fit of P2 irradiated with UVB for 30 min and 90 min are offset by 10 and 100, respectively; (B) SEC traces of polymers obtained from UVB irradiation of the P2 dispersion at different time points; (C) overlay SEC traces of the polymer obtained from 90 min UVB irradiation of P2 dispersion in water and 30 min UVB irradiation of its solution in DMAc; (D) schematic representation of the postulated photodegradation process of P2 in water. (E) Effect of the environmental confinement on telechelic coumarin terminated PDMA segments, shifting their photostationary state under UVB irradiation.

The recorded data show a good fit for a polymer micelle sphere model (Pedersen model, Fig. 5A).^55^ This analysis showed a drastic decrease in the volume of the micelle core and corona after UVB irradiation of 30 min and the radius of the polymer micelle core reduced by nearly 1.5 fold (Table S4†). Further irradiation up to 90 min only led to a minor impact on the micelle structure with the micelle core remaining similar in radius. Given the structure of P2 consisting of a hydrophilic PDMA block and a P(C3-*co*-DMA) block containing hydrophobic C3, it is likely that the aggregates are micelles where the hydrophobic segments of C3 form a core with the PDMA blocks facing towards water, forming the corona. To correlate the overall reduction in the diameter of the obtained self-assembly with the changes of the polymer primary structure, the P2 dispersion in water was analyzed *via* SEC after different irradiation times (Fig. 5B). The *M*_p_ value reduced from 21 kg mol^−1^ to 13 kg mol^−1^ within 60 min of UVB irradiation at a comparable rate to the degradation in DMAc, where no self-assembled structures are expected (Fig. S9†). These results indicate that the dissociation rate of the PDMA block from the P(DMA-*co*-C5) block is not strongly affected by the assembly.^56,57^ However, unlike degradation in DMAc, the degradation products in water display fragments that are significantly larger than the pristine polymer P2, which continued to increase in molecular weight with irradiation time. After 90 min of irradiation, the SEC of the polymer showed two distinguishable polymer distributions: one at *M*_p_ similar to that of the polymer obtained from UVB degradation in DMAc (13 kg mol^−1^) and one at *M*_p_ 110 kg mol^−1^ (substantially larger than P2*M*_p_ = 21 kg mol^−1^). We postulate that the diblock copolymer P2 degraded into two different fragments: PDMA (first block) with a single terminal coumarin end group (F^1^) and shorter DMA oligomers with two coumarin end groups (F^2^, Fig. 5D). F^1^ being a singly coumarin terminated PDMA of molecular weights > 10 kg mol^−1^ can likely disperse well in water and leave the micellar assembly. The significantly shorter doubly coumarin terminated DMA oligomers F^2^ in contrast have a higher ratio of hydrophobic termini to hydrophilic PDMA and remain assembled in water with the hydrophobic coumarin end groups facing the center. The high local concentration of coumarin groups in the hydrophobic core may kinetically favour the [2 + 2] cycloaddition of coumarin and shift the dynamic reaction equilibrium of the photostationary state between cycloaddition and reversion towards cycloaddition – even under UVB irradiation, which favours photocycloreversion in well dissolved systems (Fig. 5E). Such shifts in the wavelength dependence of the photostationary state of [2 + 2] photocycloadditions have been reported for confined surface^58^ and polymer side chain tethered systems.^59,60^

The hypothesis is that the observed high molecular weight polymer with *M*_p_ = 110 kg mol^−1^ is the intermolecular photocycloaddition product of coumarin terminated DMA oligomers, F2, dissolved in DMAc, a good solvent for both PDMA and coumarin (Fig. 5C). After subsequent UVB light irradiation in DMAC for 30 min, the high molecular weight polymer distribution indeed decreased in the SEC trace (Fig. 5C). This confirms that the observed high molecular weight polymer is the result of reversible photocycloaddition polymerization of coumarin terminated oligomers. To underpin that the UVB light induced polymer degradation yields intact coumarin terminated oligomers F^2^ that can be repolymerized, a single block copolymer P(DMA-*co*-C3) was synthesized (*M*_n_ 12.5 kg mol^−1^, *Đ* 1.3, and incorporation ratio 1.4%). UVB light irradiation yielded oligomers (*M*_n_ 5.7 kg mol^−1^ and *Đ* 1.4). Subsequently, re-polymerization *via* UVA induced the [2 + 2] cycloaddition of coumarin under dilute concentration (1.5 mg mL^−1^) in water. After 1 h of UVA irradiation, which initiates selectively the [2 + 2] photocycloaddition, the SEC trace of the polymer shifted significantly to higher molecular weights, giving rise to an increase of 32% in the *M*_n_ of the polymer (Fig. S11†). As the entire polymer distribution shifts towards higher molecular weights, irreversible photodegradation of the polymer under UVB degradation could not be detected, highlighting the reversibility of the [2 + 2] photocycloaddition and reversion based de- and repolymerization.

The photodegradation of triblock P3 in water was also carried out with UV irradiation and similar observations were obtained (Fig. S12†). The derived count rate and hydrodynamic diameter of the dispersion decreased upon UVA and UVB irradiation, indicating the degradation of P3 in water. After 20 min of UVA irradiation, the *M*_p_ of the polymer reduced from 77 kg mol^−1^ to 25 kg mol^−1^ due to the dissociation of styrylpyrene photocycloadducts in the third block of P3 and decreased further to 15 kg mol^−1^ upon subsequent UVB irradiation to induce cycloreversion of coumarin photocycloadducts. The SEC trace of polymer P3 in water after UVA and UVB irradiation also exhibited two peaks: one comparable to the degraded P3 in DMAc (15 kg mol^−1^) and one much larger polymer (*M*_p_ = 70 kg mol^−1^), which is not observed in the SEC of the polymer irradiated with UVA for 20 min, showing that rephotopolymerization progressed with prolonged UV irradiation as observed for P2. In a good solvent, the higher molecular weight polymer depleted in the SEC chromatogram when the polymer was dissolved in DMAC and exposed to UVA light for 30 min.

## Conclusion

We report block selective polymer degradation as a function of irradiation wavelengths and assembly: (1) using a pair of two cyclic allylsulfide monomers obtained from the intramolecular [2 + 2] photocycloaddition of either styrylpyrene (SC5) or coumarin (C3), radical ring-opening copolymerization yielded polymers that degrade at different wavelengths. The selective incorporation of these different photoreactive gates into different blocks of vinylic polymer using combinations of rROP and (PET) RAFT gave rise to degradable multi-block copolymers including PDMA-*block*-P(C3-*co*-DMA)-*block*-(SC5-*co*-DMA), P3. In organic solvents, UVA irradiation yielded degradation of the SC5 containing block, whereas subsequent UVB irradiation degraded the C3 containing block. (2) Utilizing the hydrophobicity of the photodegradable monomers, these primary sequences assembled in water into micelles, whose sizes were adjustable through irradiation degradation of the polymer main chain. (3) Finally, it was observed that the self-assembled state also exerted control over the photochemistry, toggling between depolymerization and depolymerization/repolymerization at identical wavelengths (*e.g.*Fig. 5E). This behaviour contrasts with previous elegant systems, where, for instance, reduction-controlled degradation of the polymer main chain governs the morphological transitions of self-assembled polymer structures in a one-way direction from chain to morphology.^61^ The interplay of main chain architecture and higher order assemblies with different wavelengths of light is thus relevant not only for natural but also for synthetic polymers.

## Data availability

The data supporting this article have been included as part of the ESI.†

## Author contributions

P. T. D. – investigation, formal analysis, and writing – original draft; F. S. – formal analysis and writing – review & editing; H. K. – formal analysis and writing – review & editing; A. S. – formal analysis and writing – review & editing; C. Z. – writing – review & editing; L. D. T. – formal analysis; D. V. G. – writing – review & editing; R. C. – review & editing; B. L. J. P. – funding acquisition and writing – review & editing; H. F. – conceptualization, funding acquisition, and writing – review & editing.

## Conflicts of interest

There are no conflicts to declare.

## Supplementary Material

SC-015-D4SC02172J-s001
