# Supplementary material for: Main chain selective polymer degradation: controlled by the wavelength and assembly
Source: Chem Sci. 2024 Jul 2;15(31):12410–9. doi: 10.1039/d4sc02172j (PMC11304539; doi:10.1039/d4sc02172j)
Supplement: SC-015-D4SC02172J-s001 [file SC-015-D4SC02172J-s001.pdf]

Electronic Supplementary Material (ESI) for Chemical Science.
This journal is © The Royal Society of Chemistry 2024

Supporting Information

Main Chain Selective Polymer Degradation: Controlled by Wavelength and Assembly

Phuong T. Do,[1,2] Federica Sbordone,[1,2] Henrik Kalmer,[1,2] Anna Sokolova,[6] Chao Zhang,[1,2,5] Linh Duy Thai,[1,2] Dmitri V. Golberg,[1,2] Robert Chapman,[3,4] Berwyck L. J. Poad, [1,2,5]* Hendrik Frisch[1,2]*

[1] School of Chemistry and Physics, Queensland University of Technology (QUT), 2 George Street, Brisbane, QLD 4000, Australia.

[2] Centre for Materials Science, Queensland University of Technology (QUT), 2 George Street, Brisbane, QLD 4000, Australia.

[3] Centre for Advanced Macromolecular Design, School of Chemistry, UNSW Sydney, Kensington, NSW 2052, Australia.

[4] School of Environmental and Life Sciences, University of Newcastle, Callaghan, NSW 2308, Australia.

[5] Central Analytical Research Facility, Queensland University of Technology (QUT), 2 George Street, Brisbane, QLD 4000, Australia.

[6] Australian Centre for Neutron Scattering, Australian Nuclear Science and Technology Organisation, New Illawarra Road, Lucas Heights, NSW 2234, Australia.

Contents

1. Additional data 4
2. Experimental details 13
SEC-ESI-MS 13
ESI-HRMS 14
THF-SEC 14
DMAc-SEC 14
NMR Measurements 15
UV-VIS Spectroscopy 15
Dynamic Light Scattering Measurements 15
Differential Scanning Calorimetry Measurement 15
Small-Angle Neutron Scattering Measurements 15
Degradation in DMAc with UV light 16
Degradation in water with UV light 16
Depolymerization and re-polymerization of a copolymer P(DMA-co-C3) 16
3. Synthesis of coumarin cyclic monomer (C3) 17
4. Synthesis of styrylpyrene cyclic monomer 18
4.1. SC1 19
4.2. SC2 20
4.3. SC3 21
4.4. SC4 22
4.5. SC5 23
5. Synthesis of polymer 25
5.1. Synthesis of copolymer of styrylpyrene monomer and DMA 25
5.2. Synthesis of copolymers and dicblock copolymers of coumarin based C3 monomer and DMA 26
5.3. Synthesis of triblock polymer 28
6. Calculation and estimation 28
6.1. Estimation of copolymer P1 28
a) Estimation of degree of polymerization (DP) 28
b) Estimation of polymer degradation 29
6.2. Estimation of diblock copolymer P2 30
a) Estimation of degree of polymerization 30
b) Estimation of polymer degradation 31
6.3. Calculation and estimation of triblock copolymer P3 31
a) Calculation of incorporation ratio of photoresponsive groups 31
b) Estimation of degree of polymerization 32
c) Estimation of photodegradation 33
7. SANS APENDIX 37

8. DOSY APENDIX 41
Diblock polymer P2 41
Triblock polymer P3..44

1. Additional data

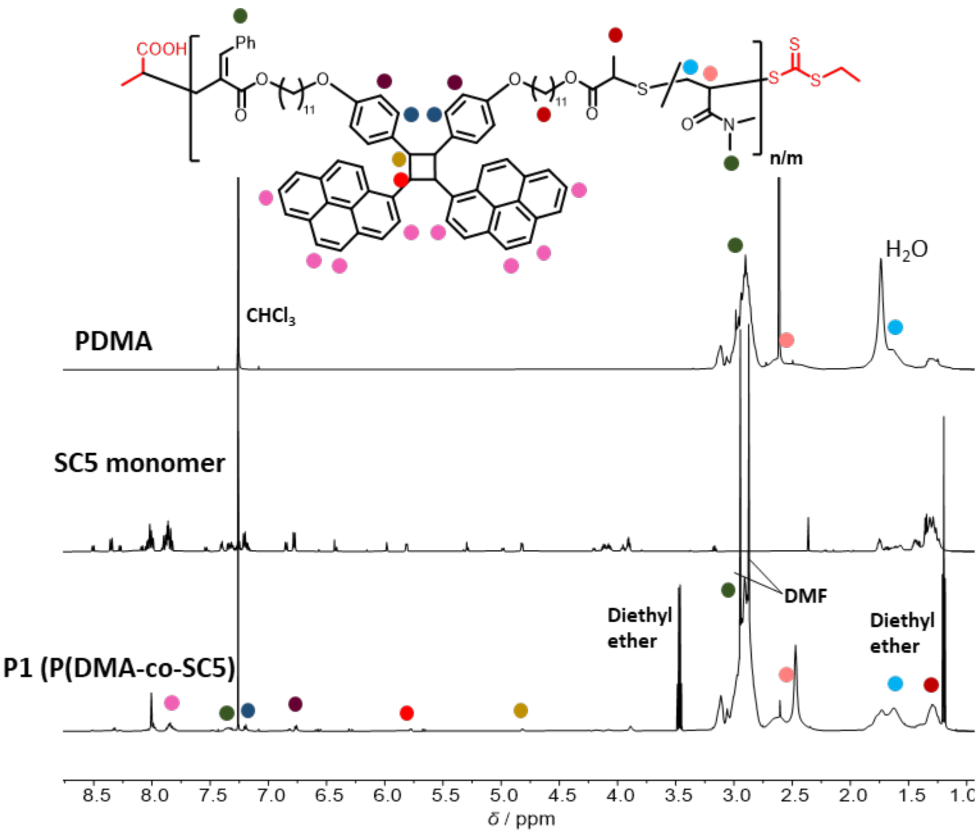

Figure S 1. Stacked [1]*H NMR spectra of homopolymer PDMA, cyclic monomer* ***SC5*** *and copolymer* ***P1*** *(P(DMA-co-****SC5****)) in $CDCl_3$* (for the full assignment of **SC5** see Fig. S20). *Integral of dimethyl proton in PDMA (δ= 2.9 ppm) is obtained from subtraction of integral of DMF protons.*

Table S 1. Table representing simulated mass to charge ratio m/z (the most abundant peak) of expected DMA oligomers with different numbers of DMA repeat units (chemical structure depicted in figure S3 (bottom)) and m/z found in the actual mass spectrum of photodegraded copolymer of styrylpyrene monomer and DMA (figure 3D in main manuscript), and their mass deviation.

charge state 2+ adduct Na+

DMA unit	Molecular formula of ion	*m/z* theo	*m/z* found	Δ *m/z* (ppm)
15	C158H221N15Na2O21S	1371.8113	1371.8136	1.68
16	C163H230N16Na2O22S	1421.3455	1421.3489	2.39
17	C168H239N17Na2O23S	1470.8797	1470.8821	1.63
18	C173H248N18Na2O24S	1520.4139	1520.4167	1.84
19	C178H257N19Na2O25S	1569.9481	1569.9508	1.72
20	C183H266N20Na2O26S	1619.4823	1619.4847	1.48
21	C188H275N21Na2O27S	1669.5182	1669.5215	1.98
22	C193H284N22Na2O28S	1719.0524	1719.0552	1.63
23	C198H293N23Na2O29S	1768.5866	1768.5892	1.47
24	C203H302N24Na2O30S	1818.1208	1818.1241	1.82
25	C208H311N25Na2O31S	1867.655	1867.6578	1.50
26	C213H320N26Na2O32S	1917.1892	1917.192	1.46
27	C218H329N27Na2O33S	1966.7234	1966.7267	1.68
28	C223H338N28Na2O34S	2016.2576	2016.2603	1.34
29	C228H347N29Na2O35S	2065.7918	2065.7939	1.02
30	C233H356N30Na2O36S	2115.326	2115.3284	1.13

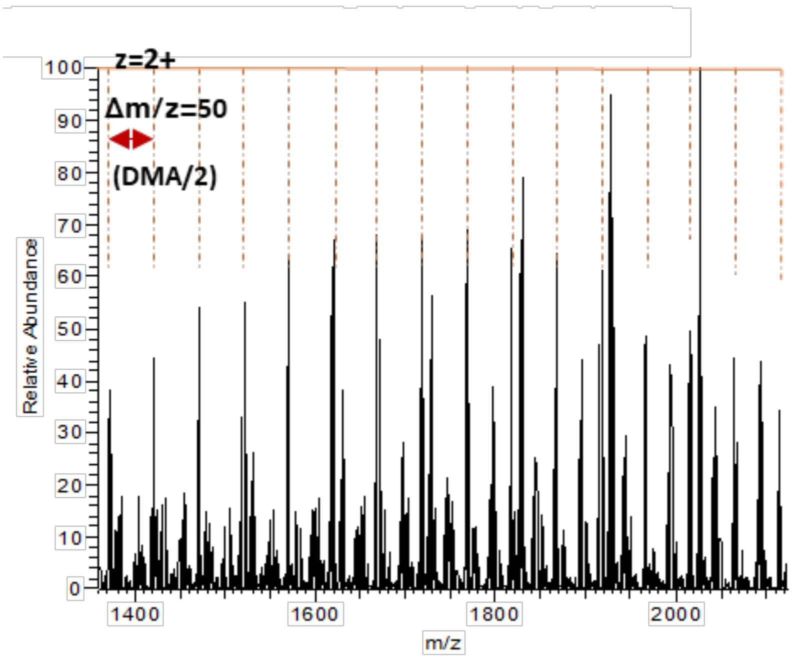

*Figure S 2. Mass spectrum at elution time 19.0 mins acquired from SEC-MS measurement of the copolymer **P1** irradiated with UVA 30 min.*

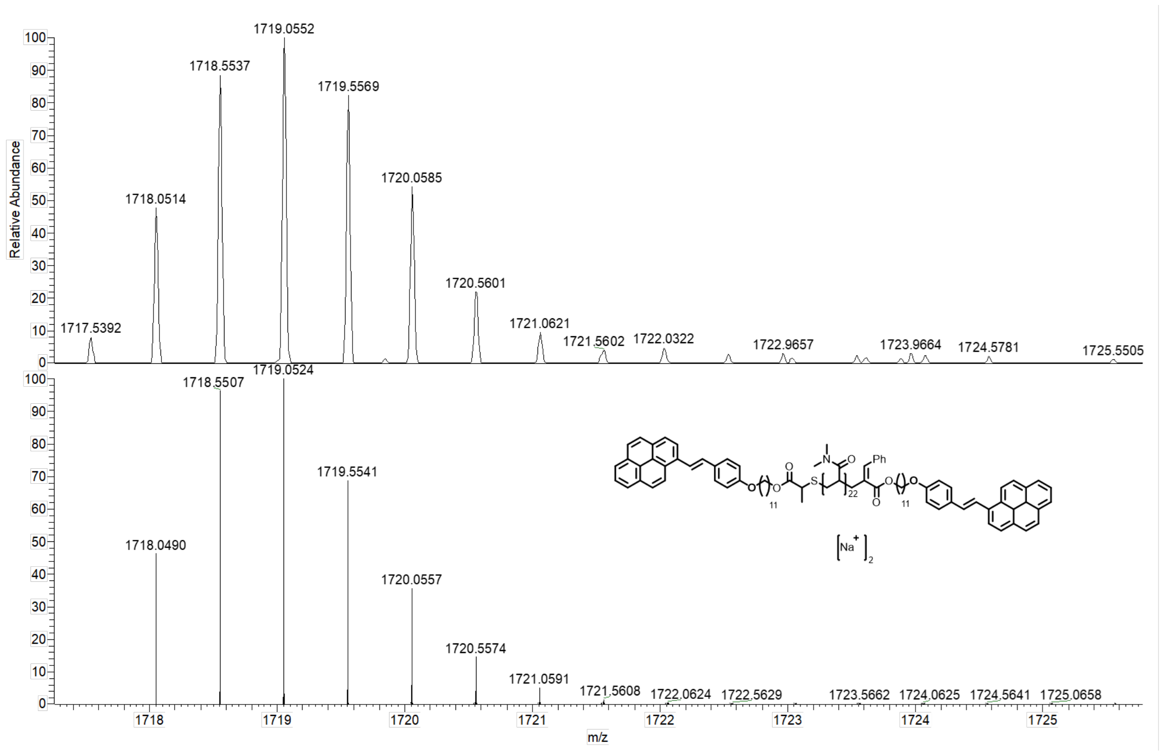

Figure S 3. A zoom in of mass spectrum of retention time 19.0 min for degraded copolymer of styrylpyrene monomer ***SC5*** *and DMA (upper) and the simulated for doubly charged sodiated oligomer ion with 22 DMA unit and expected styrylpyrene end groups (lower), showing good agreement between found m/z and simulated m/z.*

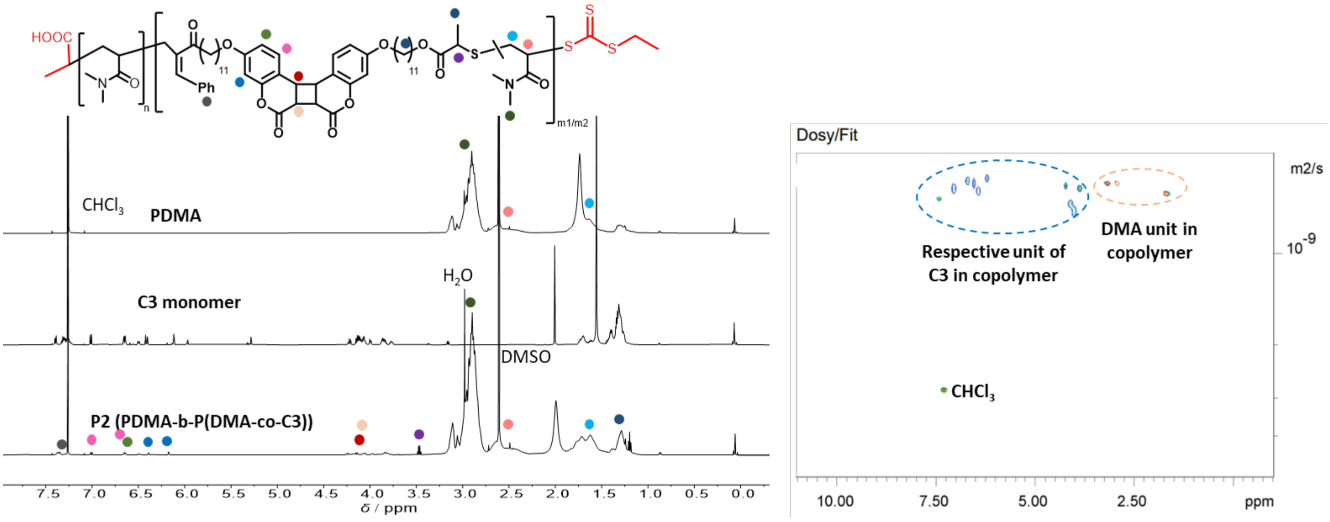

Figure S 4. Left: Stacked [1]*H NMR spectra of homopolymer PDMA, cyclic monomer* ***C3*** *and diblock copolymer* ***P2*** *(PDMA-b-P(DMA-co-****C3****)) in* $CDCl_3$ (for a full assignment of **C3** see Fig. S13)*; Right: DOSY NMR of polymer* ***P2*** *showing similar diffusion coefficients for coumarin and DMA, indicative of successful chain extension and copolymerization;*

Table S 2. Table representing simulated mass to charge ratio m/z (the most abundant peak) of expected DMA oligomers with different numbers of DMA repeat units (chemical structure depicted in figure S7 (bottom)) and m/z found in the actual mass spectrum of photodegraded diblock copolymer of coumarin monomer ***C3*** *and DMA (figure 6D in main manuscript), and their mass deviation.*

charge state 2+ adduct Na+

DMA unit	Molecular formular of ion	*m/z* theo	*m/z* found	Δ *m/z* (ppm)
20	C153H246N20Na2O30S	1461.3939	1461.399	3.49
21	C158H255N21Na2O31S	1510.9281	1510.9252	-1.92
22	C163H264N22Na2O32S	1560.4623	1560.4667	2.82
23	C168H273N23Na2O33S	1609.9965	1610.0028	3.91
24	C173H282N24Na2O34S	1659.5307	1659.5353	2.77
25	C178H291N25Na2O35S	1709.0649	1709.0613	-2.11
26	C183H300N26Na2O36S	1758.5991	1758.6034	2.44
27	C188H309N27Na2O37S	1808.635	1808.6387	2.05
28	C193H318N28Na2O38S	1858.1692	1858.1743	2.74
29	C198H327N29Na2O39S	1907.7034	1907.7054	1.05
30	C203H336N30Na2O40S	1957.2376	1957.2439	3.22
31	C208H345N31Na2O41S	2006.7718	2006.777	2.59
32	C213H354N32Na2O42S	2056.306	2056.3105	2.19
33	C218H363N33Na2O43S	2105.8402	2105.8435	1.57
34	C223H372N34Na2O44S	2155.3744	2155.3774	1.39
35	C228H381N35Na2O45S	2204.9087	2204.9158	3.22
36	C233H390N36Na2O46S	2254.4429	2254.4473	1.95
37	C238H399N37Na2O47S	2303.9771	2303.9812	1.78

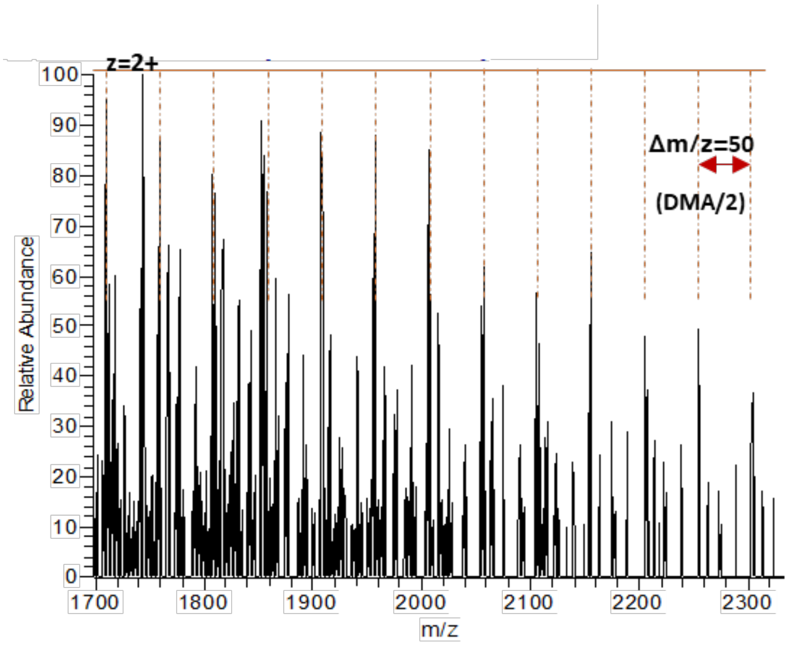

*Figure S 5. Mass spectrum at elution time 20.45 min acquired from SEC-MS measurement of copolymer **P2** irradiated with UVB 30 min.*

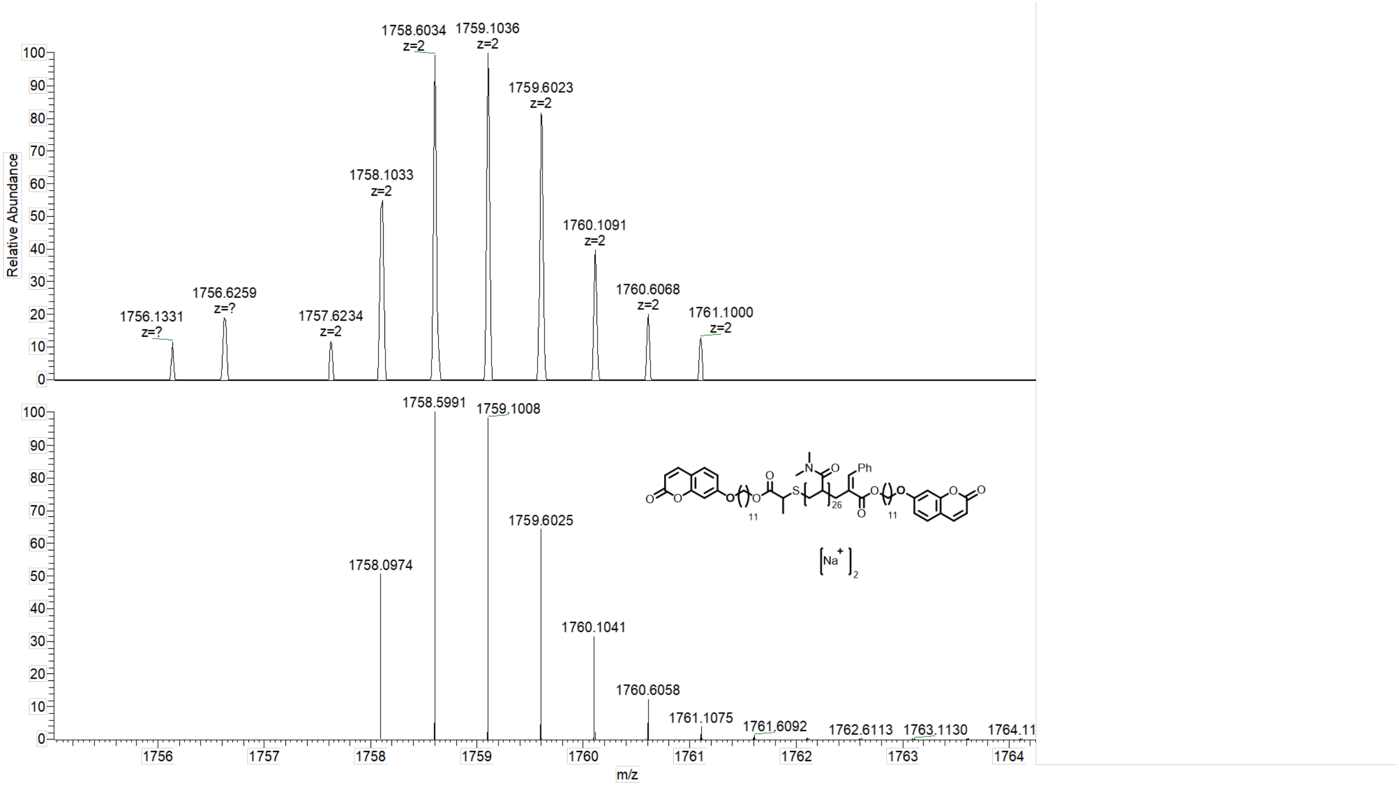

*Figure S 6. A zoom in of mass spectrum of retention time 20.45 min for degraded diblock copolymer of coumarin monomer **C3** and DMA (upper) and the simulated for doubly charged sodiated oligomer ion with 26 DMA unit and expected coumarin end groups (lower), showing good agreement between found m/z and simulated m/z.*

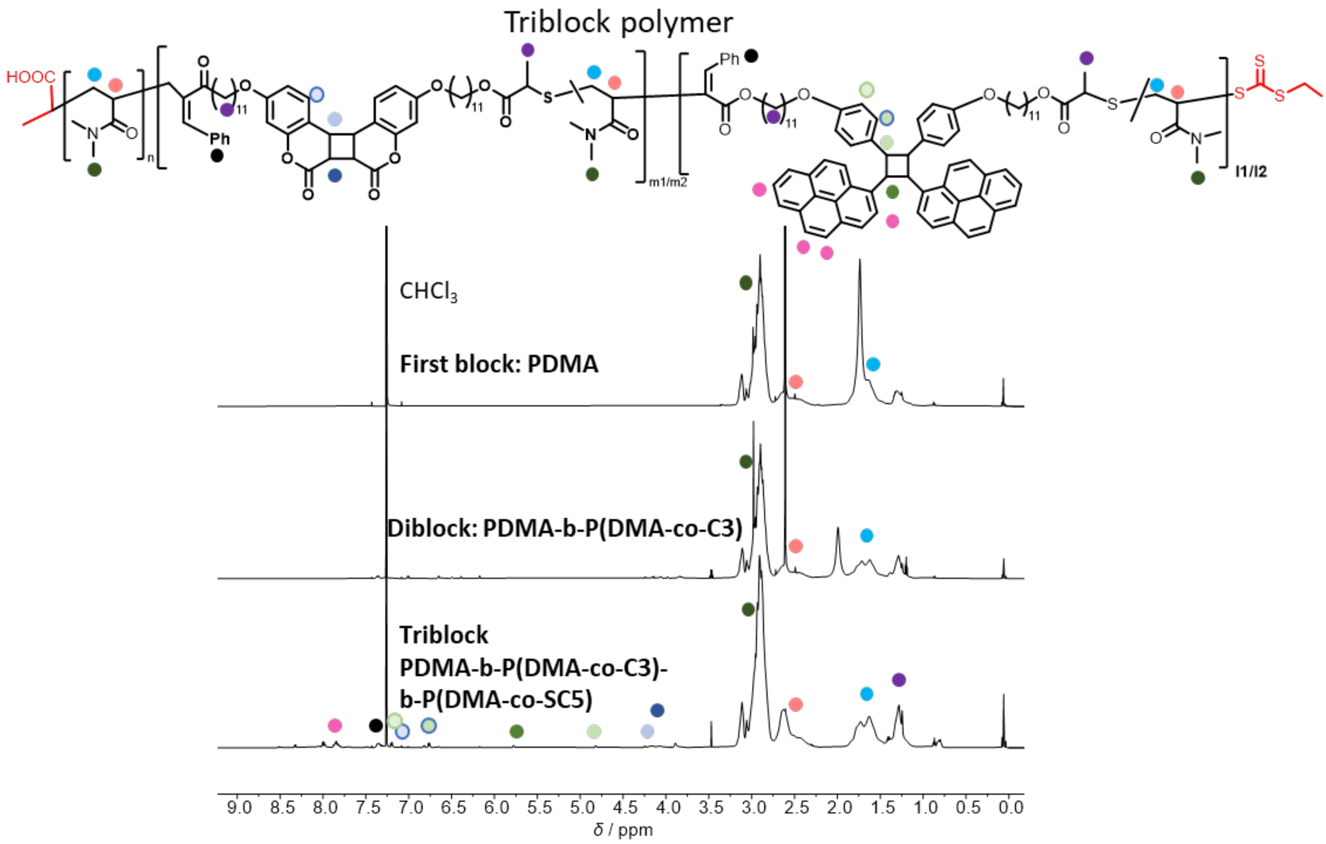

Figure S 7. Stacked ^{1}*H NMR spectra of homopolymer PDMA, diblock polymer* ***P2*** *(PDMA-b-P(DMA-co-****C3****)) and triblock polymer* ***P3*** *(PDMA-b-P(DMA-co-****C3****)-b-P(DMA-co-****SC5****)) in* $CDCl_3$ (See Fig. 4A for a zoom into the region between δ = 8.8 – 3.6 ppm).

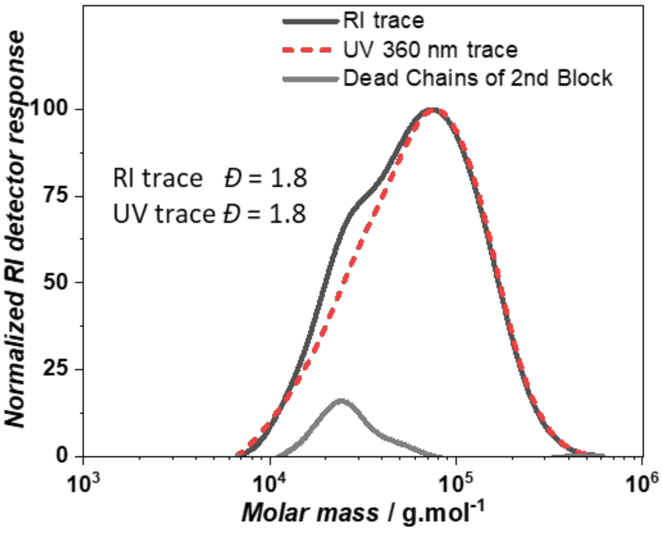

	peak area
RI trace	1.62×10^{7}
Dead chains of second block	0.07×10^{7}
**Ratio of dead chain to triblock P3**	**< 5%**

Figure S 8. Left: Overlay of RI trace and UV (λ = 360 nm) trace in SEC measurement of ***P3*** *and distribution of dead chains of second block obtained from subtraction of RI trace to UV (λ = 360 nm) trace. Right: the peak area integrated from respective peaks. The amount of dead chain of second block to chain extension product- triblock P3 is calculated from peak area of dead chain peak and UV trace peak.*

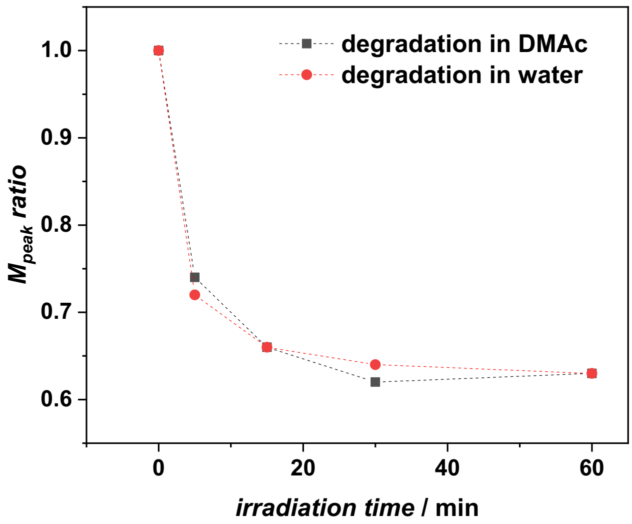

*Figure S 9. Comparison of photodegradation of diblock polymer **P2** in DMAc solvent and in water: reduction of averaged molecular weight (Mn) of polymer represented by ratio of Mp of polymer at t min UVB irradiation to Mn of pristine polymer plotted vs UVB irradiation time.*

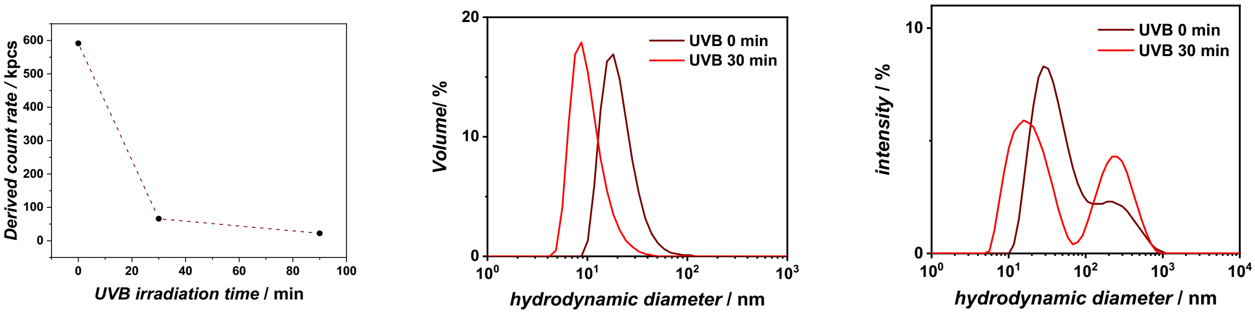

*Figure S 10. A dispersion of diblock copolymer **P2** in water (1.5 mg mL^{-1}) was irradiated with UVB for 90 min. Derived count rate of dispersion plotted vs UVB irradiation time (left), hydrodynamic diameter distribution by volume of the dispersion at UVB irradiation time 0 min, 30 min (middle), and hydrodynamic diameter distribution by intensity of the dispersion at UVB irradiation time 0 min, 30 min (right).*

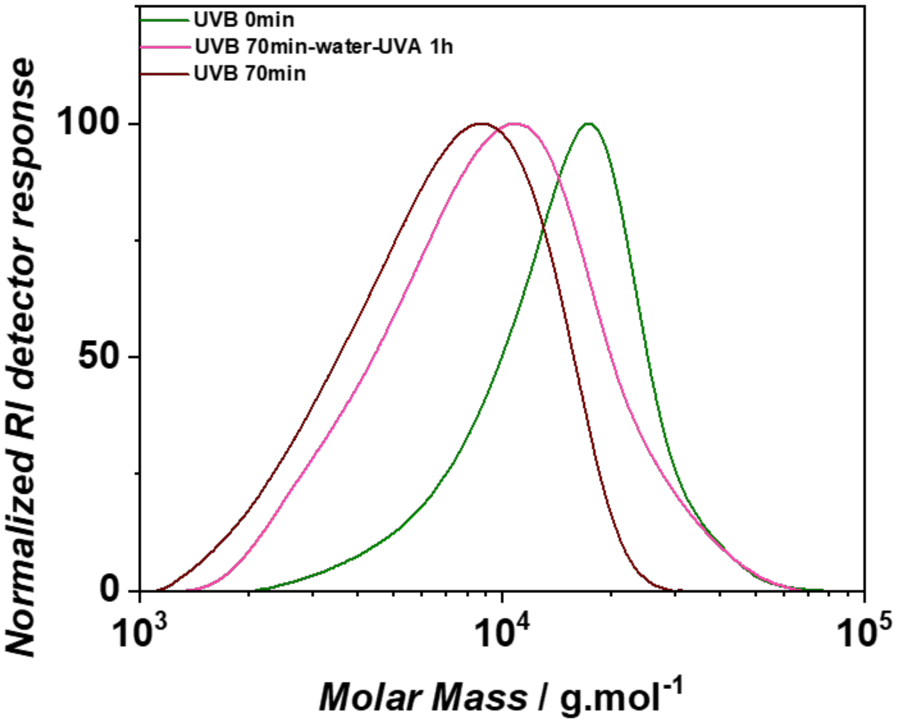

	UVB 0min	UVB 70 min	UVB 70min-water-UVA 1h
M_n (kg.mol^{-1})	12.5	5.7	7.5
M_p(kg.mol^{-1})	17.2	8.2	10.5
Đ	1.32	1.41	1.54

*Figure S 11. SEC traces and respective molar weight and dispersity during the depolymerization and repolymerization of a copolymer P(DMA-co-**C3**) (Mn 12.5 kg.mol^{-1}, Đ 1.3, incorporation ratio 1.4%). Refer to section 2 for detailed experiment.*

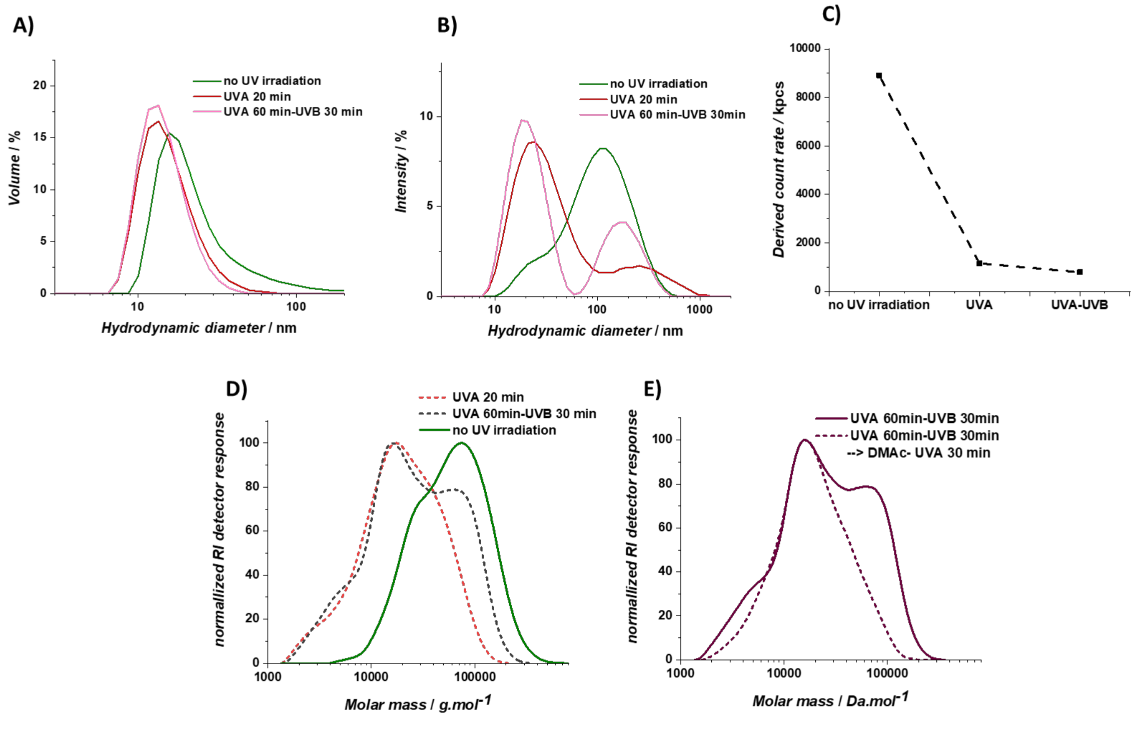

*Figure S 12. Degradation of **P3** in water (2 mg.mL^{-1}) under UV irradiation: A) Hydrodynamic diameter distribution by volume of the dispersion with UVA and UVB irradiation obtained via DLS; B) Hydrodynamic diameter distribution by intensity of the dispersion with UVA and UVB irradiation obtained via DLS; C) Derived count rate of dispersion plotted vs UV irradiation; D) overlay SEC of **P3**, **P3** dispersion irradiated with UVA 20 min, and UVA 60 min- UVB 30 min ; E) Overlay SEC traces of the polymer obtained from UVA- UVB irradiation of **P3** dispersion in water and 30-min UVA irradiation of its solution in DMAc.*

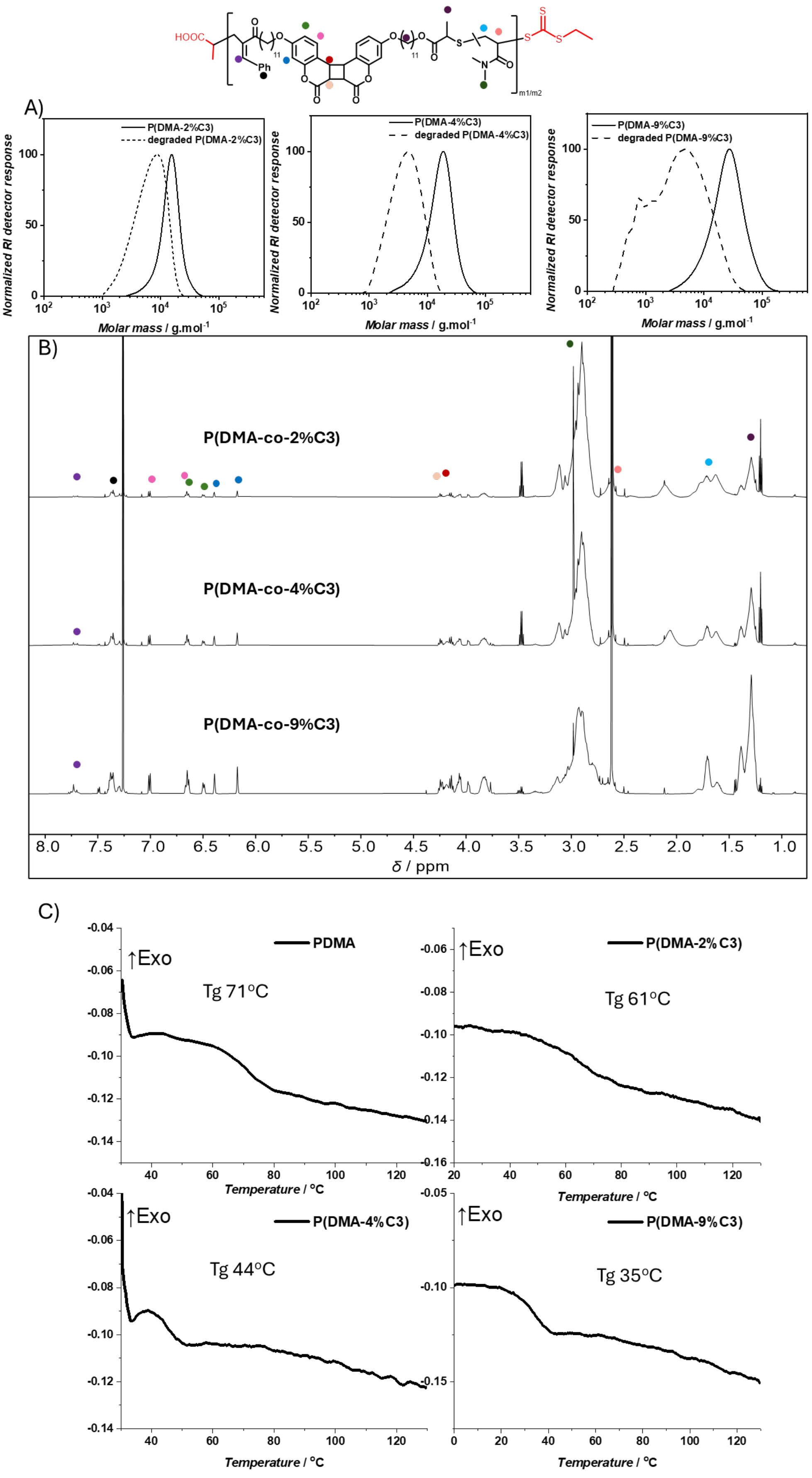

*Figure S 13. Copolymers of DMA and **C3** prepared at different molar ratios: (A) SEC chromatograms of the copolymers and the copolymers irradiated with UVB for 30min: P(DMA-2%C3) Mn = 13 kg mol^{-1}, Đ = 1.2, degraded polymer Mn = 5.3 kg mol^{-1}; P(DMA-4%C3) Mn = 14 kg mol^{-1}, Đ = 1.3, degraded polymer Mn = 3.5 kg mol^{-1}; P(DMA-9%C3) Mn = 20 kg.mol^{-1}, Đ = 1.5, degraded polymer Mn = 1.9 kg mol^{-1}; (B) ^{1}H NMR acquired in $CDCl_3$; (C) DSC measurement of copolymers from (A) and pure PDMA Mn = 19 kg mol^{-1}.*

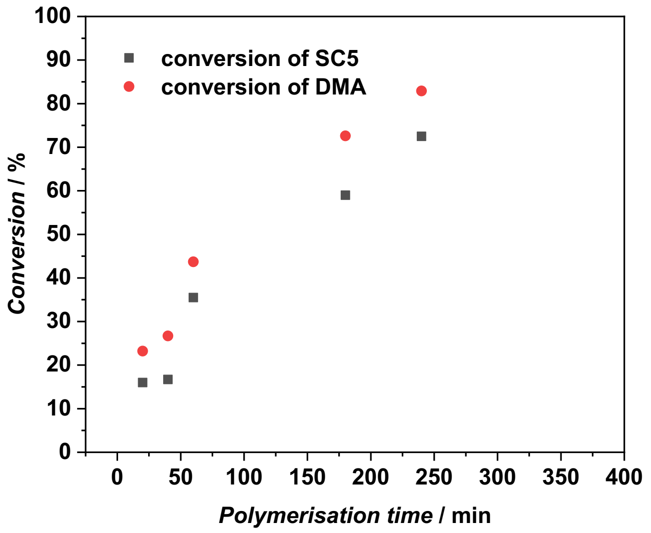

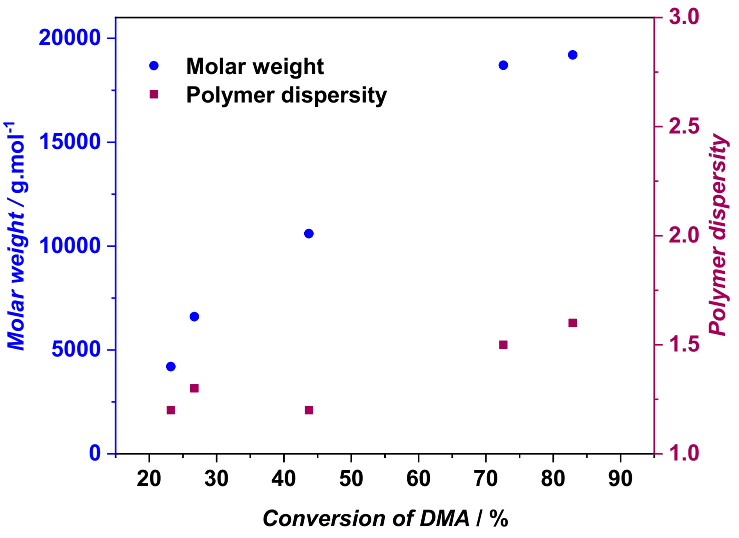

*Figure S 14. Plot of **SC5** and DMA conversion vs polymerisation time (top) and polymer molecular weight and polymer dispersity plotted vs DMA conversion (bottom). Refer to section 6.4 for detailed calculations.*

2. Experimental details

SEC-ESI-MS

Size exclusion chromatography coupled with electrospray ionisation mass spectrometry experiments were measured using a Q Exactive Plus Biopharma mass spectrometer (Thermo Fisher Scientific, San Jose, CA, USA) equipped with a HESI-II ionisation source. The mass spectrometer was calibrated up to *m/z* 2000 using premixed calibration solution (Pierce; Thermo Scientific) and for high mass mode (*m/z* 600-8000) using ammonium hexafluorophosphate solution. A constant spray voltage of +3.5 kV, a dimensionless sheath gas and a dimensionless auxiliary gas flow rate of 10 and 0 were applied, respectively. The capillary temperature was set to 320 °C, the S-lens RF level was set to 150 and the auxiliary gas heater temperature was set to 125 °C. The Q Exactive was coupled to an UltiMate 3000 UHPLC System (Dionex, Sunnyvale, CA, USA) consisting of a pump (LPG 3400SD), autosampler (WPS 3000TSL), and a temperature-controlled column department (TCC 3000).

Separation was performed on two mixed bed size exclusion chromatography columns (Agilent, Mesopore 250 × 4.6 mm, particle diameter 3 μm) with a precolumn (Mesopore 50 × 7.5 mm) operating at 30 °C. THF at a flow rate of 0.30 mL min^{-1} was used as eluent. The mass spectrometer was coupled to the column in parallel with an UV-detector (VWD 3400, Dionex) and a Refractive Index detector (RefractoMax520, ERC, Japan) as described earlier.[1] A split flow of 0.27 mL min^{-1} of the eluent were directed through the UV- and RI-detector and the remaining 30 μL min^{-1} directed into the electrospray source following post-column addition of 50 μM sodium iodide in methanol at 20 μL min^{-1} by a micro-flow HPLC syringe pump (Teledyne ISCO, Model 100DM). A 100 μL aliquot of polymer solution at 2 mg mL^{-1} concentration was injected into the SEC system for analysis.

ESI-HRMS

The working solution was prepared from stock solution of analyte sample 0.05 mg mL^{-1} and methanolic solution of sodium acetate 0.2mM with the volume ratio of 10:2. The working solution was then infused directly to the MS system at constant flow rate of 5 μL min^{-1} via a 500 μL syringe controlled by a syringe pump (Pump 11 Elite, Harvard Apparatus). Spectra were recorded on the same Q Exactive Plus Biopharma mass spectrometer as described above for the SEC-ESI-MS experiments, operating in positive ion mode. A constant spray voltage of 3.0 kV, a dimensionless sheath gas and a dimensionless auxiliary gas flow rate of 25 and 10 were applied, respectively. The capillary temperature was set to 320 °C, the S-lens RF level was set to 60, and the auxiliary gas heater temperature was set to 100 °C.

THF-SEC

The SEC measurements were conducted on a PSS SECurity^2 system consisting of a PSS SECurity Degasser, PSS SECurity TCC6000 Column Oven (35 °C), PSS SDV Column Set (8 x 150 mm 5 μm Precolumn, 8 x 300 mm 5 μm Analytical Columns, 100000 Å, 1000 Å and 100 Å) and an Agilent 1260 Infinity Isocratic Pump, Agilent 1260 Infinity Standard Autosampler, Agilent 1260 Infinity Diode Array and Multiple Wavelength Detector (A: 254 nm, B: 360 nm), Agilent 1260 Infinity Refractive Index Detector (35 °C). HPLC grade THF, stabilized with BHT, is used as eluent at a flow rate of 1 mL·min^{-1}. Narrow disperse linear poly (methyl methacrylate) ($\overline{M_n}$: 202 g·mol^{-1} to 2.2x106 g·mol^{-1}) standards (PSS ReadyCal) were used as calibrants. All samples were passed over 0.22 μm PTFE membrane filters. Molecular weight and dispersity analysis was performed in PSS WinGPC UniChrom software (version 8.2).

DMAc-SEC

SEC measurements were conducted on a PSS SECurity2 system consisting of a PSS SECurity Degasser, PSS SECurity TCC6000 Column Oven (60 °C), PSS GRAM Column Set (8x150 mm 10 μm Precolumn, 8x300 mm 10 μm Analytical Columns, 1000 Å, 1000 Å and 30 Å) and an Agilent 1260 Infinity Isocratic Pump, Agilent 1260 Infinity Standard Autosampler, Agilent 1260 Infinity Diode Array and Multiple Wavelength Detector (A: 254 nm, B: 360 nm), Agilent 1260 Infinity Refractive Index Detector (35 °C). HPLC grade DMAc, 0.01 M LiBr, is used as eluent at a flow rate of 1 mLmin^{-1}. Narrow disperse linear poly(styrene) (Mn: 266 g·mol-1 to 2.52x106 g·mol^{-1}) and poly(methyl methacrylate) (Mn: 202 g·mol-1 to 2.2x106 g·mol-1) standards (PSS ReadyCal) were used as calibrants. All samples were passed over 0.22 μm PTFE membrane filters. Molecular weight and dispersity analysis was performed in PSS WinGPC UniChrom software (version 8.2).

NMR Measurements

^{1}H- and COSY spectra were recorded on a Bruker System 600 Ascend LH, equipped with a BBO-Probe (5 mm) with z-gradient (1H: 600.13 MHz, 13C: 150.90 MHz,). All measurements were carried out in deuterated solvents. The chemical shift (δ) is recorded in parts per million (ppm) and relative to the residual solvent protons.[2] The measured coupling constants were calculated in Hertz (Hz). To analyze the spectra the software MESTRENOVA 11.0 was used. The signals were quoted as follows: s = singlet, bs = broad singlet, d = doublet, t = triplet, dd = doublet of doublets and m = multiplet.

Diffusion Ordered Spectroscopy (DOSY) NMR experiments based on 1H-NMR were performed at 35°C on a Bruker 400 Advance III HD spectrometer equipped with either BBO or Quattro Nucleus Probes (QNP) (5mm) with z-gradients (1H: 400.16 MHz). A sequence with longitudinal eddy current delay (LED) using bipolar gradients (Bruker ledbpgp2s) was employed in order to compensate for eddy currents. The diffusion gradient length δ and diffusion delay Δ were determined separately for each sample. Gradient strength was linearly incremented from 2% at 0.96 Gcm-1 to 95% at 45.7 Gcm-1 in 20 steps. The obtained data was processed with TopSpin 4.0.6 and Dynamics Center 2.5.3. After Fourier transformation of the 1D spectra, the signal decay with respect to gradient strength G was fitted to:

$$f(G) = I_0 e^{-DG^2\gamma^2\delta^2\left(\Delta-\frac{\delta}{3}\right)} \cdot 10^4$$

with γ = gyromagnetic ratio of 1
H and I0 = full signal intensity.

UV-VIS Spectroscopy

UV/vis spectra were recorded on a Shimadzu UV-2700 spectrophotometer equipped with a CPS-100 electronic temperature control cell positioner. Samples were prepared in THF and measured in Hellma Analytics quartz high precision cells with a path length of 10 mm at ambient temperature.

Dynamic Light Scattering Measurements

All dynamic light scattering (DLS) measurements were performed using a Malvern Zetasizer Nano-ZS instrument (Malvern Instruments) equipped with a backscattering detector (measurement angle = 175°).

Differential Scanning Calorimetry Measurement

DSC measurements were conducted using a Netzsch DSC 204 F1 Phoenix. Samples were dissolved in dichloromethane and dropped cast into DSC pans, followed by drying at 40 ºC at high vacuum overnight prior to DSC measurement. 3 cycles of heating/cooling ramps at scanning speed of 5 $°C \cdot min^{-1}$ were used over the temperature range from either 30 °C or -10 °C to 130 °C with isothermal exposures (10 min) at end temperatures. A nitrogen sample purge flow of 20 $mL \cdot min^{-1}$ was employed. Glass transition temperatures were determined from the 2nd heating ramp. The data was analysed using the TA Instruments Universal Analysis 2000 software (version 4.2E).

Small-Angle Neutron Scattering Measurements

Static small-angle neutron scattering (SANS) measurements were obtained on the Bilby instrument,[3, 4] in time-of-flight mode with an asymmetric detector array at the Australian Centre for Neutron Scattering (ACNS),

ANSTO, Lucas Heights, NSW. A neutron wavelength of 4–14 Å was used in these measurements, obtaining a q-range of 0.0017–0.820 $Å^{-1}$ at a single instrument configuration. Distance to main detector, rear detector, horizontal curtains, and vertical curtains was 12m, 2m and 1m, respectively. Left, right, top, and bottom curtains separations from the beam were 0.20, 0.38, 0.10 and 0.25m, respectively. Source and sample aperture diameters were 40.0 and 12.5 mm.

Samples were prepared in 1 mm (limited by available amount of sample) path-length Hellma quartz cells for a bit more than two hours (8000 sec each). Best possible contrast ($SLD_{polymer}$ = 0.9-1.1) was introduced by using D_2O (SLD = 6.36) as the solvent.

SANS data reduction including placing data on the absolute scale was done using standard Bilby procedures implemented in Mantid.[5]

SANS data was analysed using Primus software from ATSAS package[6] and also fit using Spherical polymer micelle model in SASView 5.0.5 [SasView 5.0.5. http://www.sasview.org/].

Degradation in DMAc with UV light

A quartz cuvette containing a solution of polymer in DMAc solvent (1 mg. mL^{-1}) was placed in a Luzchem photoreactor equipped with 3 UV lamps. The cuvette was irradiated with UV light for certain amount of time which is specified in the result section.

Degradation in water with UV light

Diblock polymer

1.5 mg of the polymer was dispersed in 1 mL of water. The dispersion was then filtered with 20 µm pore PTFE filter to remove any dust or unresolved solid fragment The dispersion was transferred to a quartz cuvette for photodegradation. The irradiation of the cuvette was carried out in a Luzchem photoreactor equipped with 3 UV lamps.

Triblock polymer

2 mg of the polymer was dissolved in 100 µL of THF. Subsequently, 900 µL of water was introduced to the solution for micelle formation to occur. The dispersion was then filtered with 20 µm pore PTFE filter and transferred to a quartz cuvette for photodegradation. The irradiation of the cuvette was carried out in a Luzchem photoreactor equipped with 3 UV lamps.

Depolymerization and re-polymerization of a copolymer P(DMA-co-C3)

Depolymerization

A copolymer P(DMA-*co*-**C3**) (Mn 12.5 kg.mol-1, Đ 1.3, incorporation ratio 1.4%) obtained from PET RAFT polymerization of DMA and **C3** was dissolved in THF solvent to the concentration of 1 $mg.mL^{-1}$. The solution was irradiated with UVB light for 70 min to degrade the polymer via cycloreversion of coumarin cycloadducts embedded in polymer backbone.

Re-polymerization

The resulting degraded polymer solution (containing 6 mg of polymer) was concentrated under reduced pressure over night, yielding a viscous liquid. The degraded polymer was dissolved in water to the concentration of 1.5 mg.mL^{-1}, followed by irradiation with UVA light.

3. Synthesis of coumarin cyclic monomer (C3)

Allylic sulfide cyclic monomer bearing coumarin as end groups was synthesized according to our previous publication[7].

HRMS: $[C53H66O10SNa]^+$ *m/z*= 917.4269; found: 917.4259, Δ*m/z*= 1.1ppm

^{1}H NMR (600 MHz, $CDCl_3$):

isomer 1: δ 7.39 (d, J = 7.6 Hz, 2H), 7.34 – 7.21 (m, 3H), 7.01 (d, J = 8.6 Hz, 2H), 6.65 (d, J = 8.6 Hz, 2H), 6.42 (s, 1H), 6.12 (d, J = 2.4 Hz, 2H), 5.97 (s, 1H), 5.28 (s, 1H), 4.25 – 3.97 (m, 12H), 3.16 (q, J = 7.3 Hz, 1H), 1.76 – 1.60 (m, 4H), 1.46 – 1.14 (m, 35H).

isomer 2: δ 7.39 (d, J = 7.6 Hz, 2H), 7.34 – 7.21 (m, 3H), 6.65 (d, J = 8.6 Hz, 2H), 6.49 (d, J = 8.2 Hz, 2H), 6.42 (s, 1H), 6.40 (d, J = 2.5 Hz, 2H), 5.97 (s, 1H), 5.28 (s, 1H), 3.90 – 3.72 (m, 12H), 3.16 (q, J = 7.3 Hz, 1H), 1.76 – 1.60 (m, 4H), 1.46 – 1.14 (m, 35H).

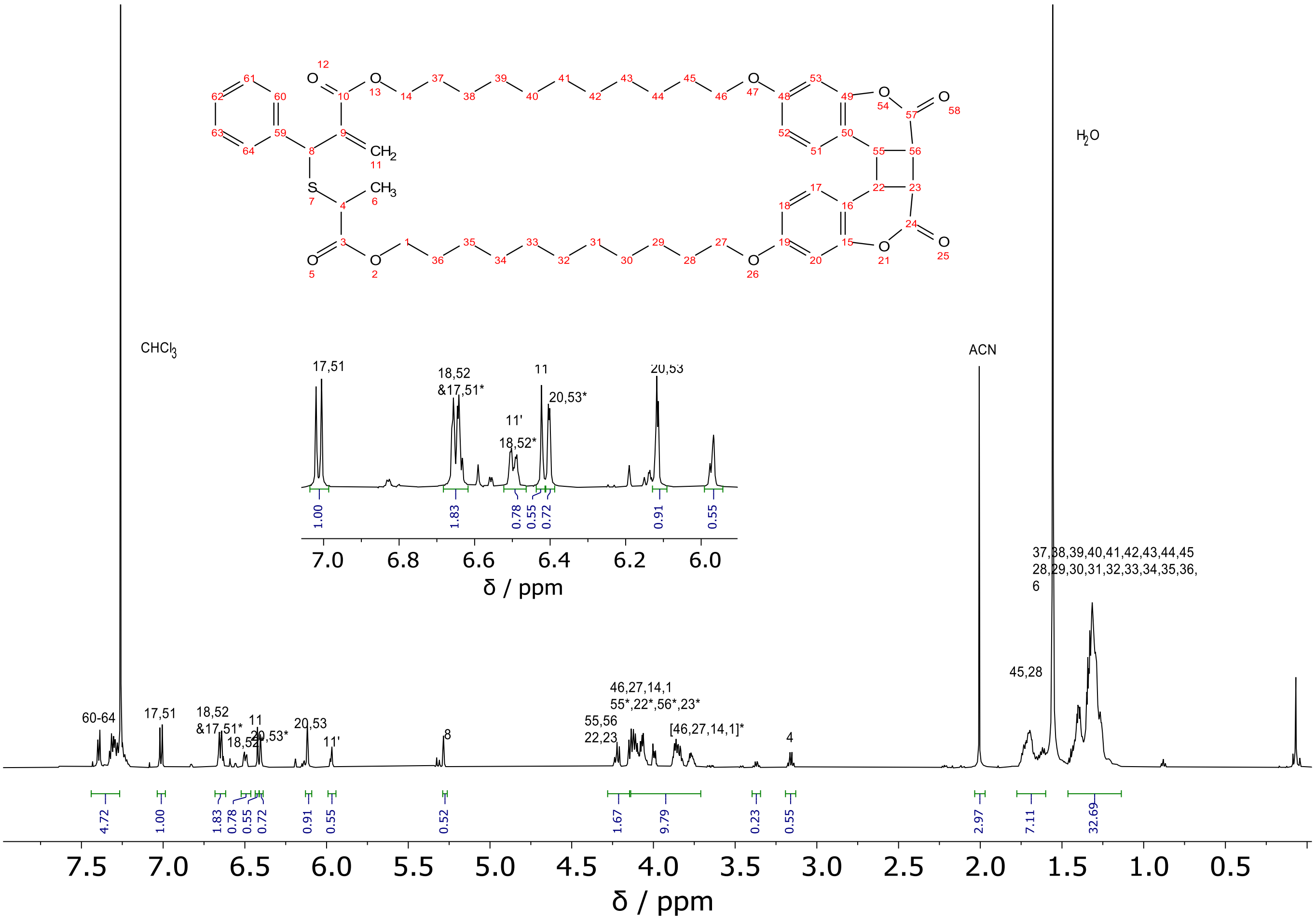

*Figure S 15. ^{1}H NMR spectrum of cyclic monomer **C3** acquired in $CDCl_3$.*

4. Synthesis of styrylpyrene cyclic monomer

Materials

Unless stated otherwise, all chemicals and solvents were used as received from the suppliers without further purification.

Benzaldehyde (Sigma- Aldrich, 99.0% min), tert-butyl acrylate (Sigma-Aldrich, 98.0% min), 1,8-Diazabicyclo [5.4.0] undec-7-ene (Sigma- Aldrich, 98.0% min), hydrochloric acid (32% in water, Thermo Fisher Scientific), sodium sulfate (anhydrous, granular, Thermo Fisher Scientific, 99.0% min), 4-methoxyphenol (Sigma-Aldrich, 99.0% min), acetic anhydride (Chem-Supply , 99.0% min), N, N-dimethylaminoprydine (Sigma-Aldrich, 99.0%), dichloromethane (Chem-Supply, 99.8%), ethyl acetate (Thermo Fisher Scientific, 99.5%), cyclohexane (Sigma-Aldrich, 99.5%), 1,4-Diazabicyclo [2.2.2] octane (Sigma-Aldrich, 99.0% min), THF (Thermo Fisher Scientific, 99.7%), 2-mercaptopropionic acid (Sigma-Aldrich, 95.0% min), trifluoracetic acid (Sigma-Aldrich, 99.0% min), acetonitrile (Fisher Scientific, 99.9% min), 4-acetoxystyrene (Acros Organics, 96 %), 1-bromopyrene (Alfa Aesar, 95 %), cyclohexane (Merck, analytical reagent grade), palladium(II) acetate (Merck, 47 % Pd), potassium hydroxide (Thermo Fischer Scientific, Analytical Reagent), hydrochloric acid (Thermo Fisher Scientific, 32 % aqueous solution), toluene (Thermo Fisher Scientific), triethylamine (Sigma-Aldrich > 99%), triphenylphosphine (Chem-supply, 99 %).11-bromo-1-undecanol (Combi-Blocks, 98.0% min), anhydrous potassium carbonate (Chem-Supply, 99.0% min), potassium iodine (Chem-Supply, 99.0%), acetone (Thermo Fisher Scientific, 99.0% min), oxalyl chloride (Sigma-Aldrich, 98.0% min), dimethylformamide (Thermo Fisher Scientific, 99.8% min), pyridine (Sigma-Aldrich, 99.0% min).

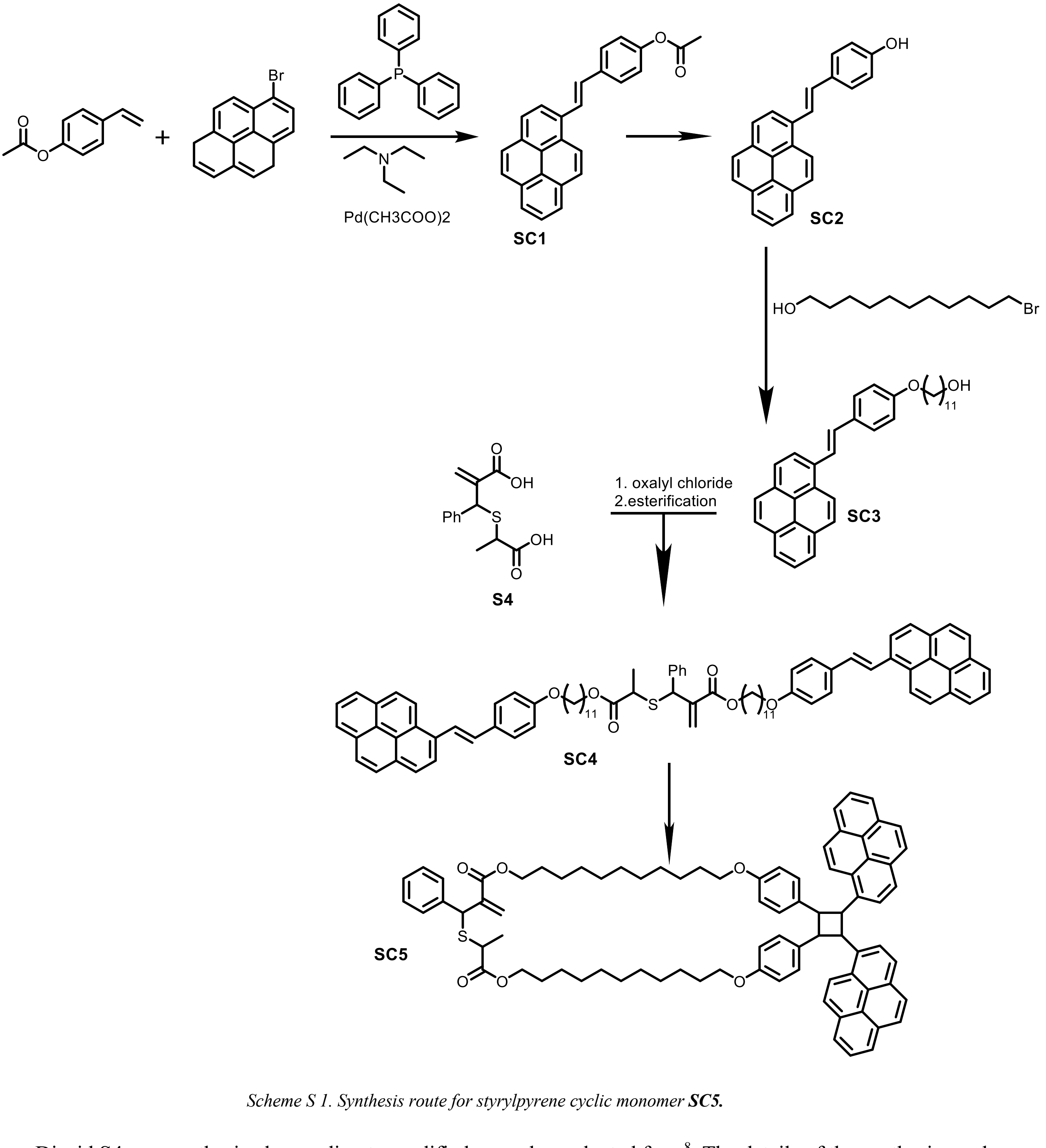

*Scheme S 1. Synthesis route for styrylpyrene cyclic monomer **SC5.***

Diacid **S4** was synthesized according to modified procedure adapted from[8]. The details of the synthesis can be found in our previous publication[7].

Synthesis of **SC1**-**SC2** was conducted following the literature-known procedure[9].

4.1. SC1

In a brown round-bottom flask, 4-acetoxystyrene (1.3 mL, 8.5 mmol, 1.2 eq), 1-bromopyrene (2.00 g, 7.0 mmol, 1.0 eq), triphenylphosphine (0.18 g, 0.7 mmol, 0.1 eq), triethylamine (9.7 mL, 70 mmol, 10 eq) were dissolved in dimethylformamide (36 mL) with stirring. The flask was degassed with Ar. After 10 minutes, palladium (II) acetate (90 mg, 0.4 mmol, 0.05 eq) was quickly added to the solution with Ar flowing. The mixture was degassed

further for 10 minutes and left stirring at 100°C for 4 days. The reaction mixture was then diluted with 50 mL of toluene and passed through an alumina column, following by washing with HCl 1M (25 mL x 3). After that, the solution was concentrated under reduced pressure and precipitated in cyclohexane to give yellow precipitate **SC1**.

Yield: 1.1 g, 3.04 mmol, 43 %

^{1}H NMR (600 MHz, $CDCl_3$) δ 8.48 (d, J = 9.3 Hz, 1H), 8.31 (d, J = 7.9 Hz, 1H), 8.21 – 8.12 (m, 5H), 8.06 (s, 2H), 8.01 (t, J = 7.6 Hz, 1H), 7.70 (d, J = 8.6 Hz, 2H), 7.33 (d, J = 16.0 Hz, 1H), 7.17 (d, J = 8.2 Hz, 2H), 2.34 (s, 3H).

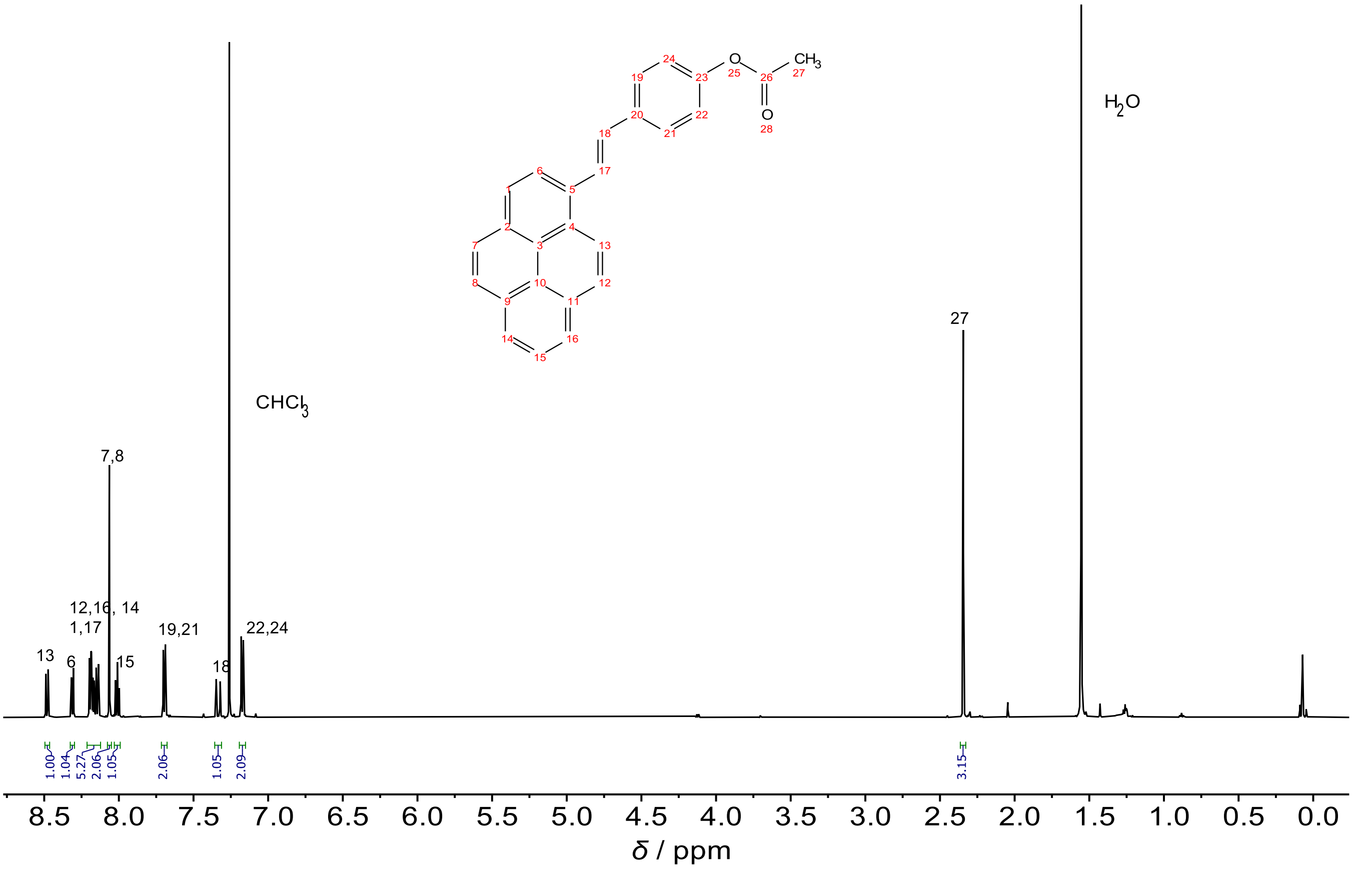

*Figure S 16. ^{1}H NMR spectrum of **SC1** acquired in $CDCl_3$.*

4.2. SC2

Acetoxystyrylpyrene **SC1** (1.1 g, 3.04 mmol, 1 eq) was dissolved in acetonitrile (62 mL) at 70 °C. A basic solution of KOH (1.7 g, 30.4 mmol, 10 eq) and 9 mL miliQ water was added dropwise into the **SC1** solution. The mixture was kept stirring at 70 °C for 16 h. After that, an acidic solution (300 mL of water, 7.7 mL of concentrate HCl) was added to the reaction mixture. The precipitate was collected via centrifugation. **SC2** was obtained in green-yellow solid.

Yield: 0.96 g, 3.0 mmol, 98 %.

HRMS: $[C24H15O]^-$ *m/z*= 319.1128; found *m/z*: 319.1129, Δ*m/z*= 0.3 ppm

^{1}H NMR (600 MHz, $CDCl_3$) δ 8.49 (d, J = 9.2 Hz, 1H), 8.30 (d, J = 8.1 Hz, 1H), 8.17 (m, 3H), 8.13 (d, J = 9.2 Hz, 1H), 8.08 – 8.03 (m, 3H), 8.00 (t, J = 7.6 Hz, 1H), 7.59 (d, J = 8.4 Hz, 2H), 7.30 (d, J = 16.0 Hz, 1H), 6.91 (d, J = 8.4 Hz, 2H), 4.95 (s, 1H).

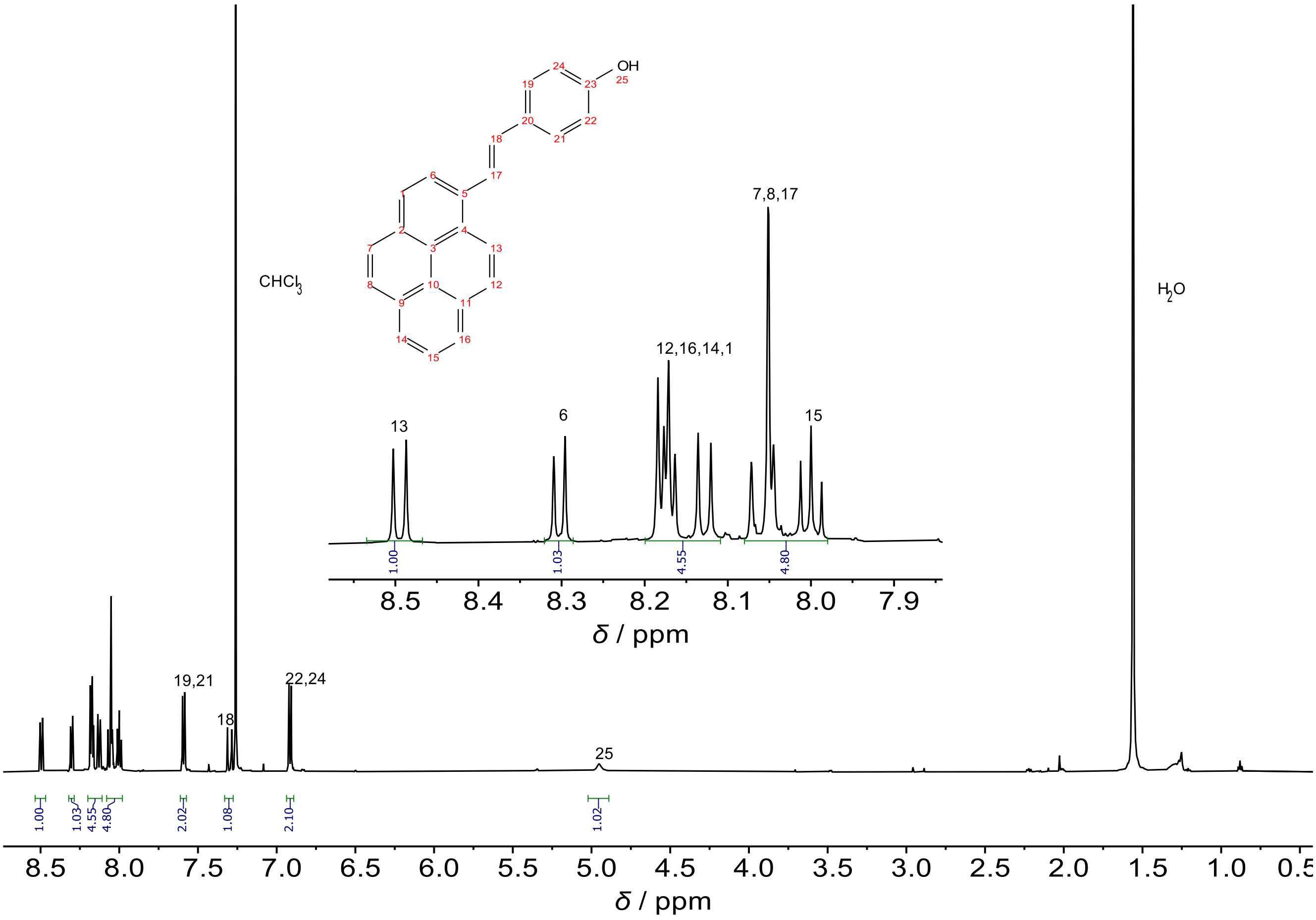

*Figure S 17. ^{1}H NMR spectrum of **SC2** acquired in $CDCl_3$.*

4.3. SC3

To a round-bottom flask, hydroxystryrylpyrene **SC2** (0.96 g, 3 mmol, 1.0 eq), 11-bromo-1-undecanol (0.75g, 3 mmol, 1.0 eq), anhydrous potassium carbonate (1.38 g, 10 mmol, 3.3 eq), 30 mg potassium iodise, and 75 mL fresh distilled acetone was added. The mixture was refluxed at 61 °C for 72 h. After that, while the mixture was still hot, the solid was filtered off and solvent was removed under reduced pressure. The obtained solid was then dissolved in dichloromethane and washed by HCl 1N. The DCM solvent was then removed to afford a yellow solid **SC3**.

Yield: 1.35 g, 2.75 mmol, 91 %.

HRMS: $[C35H38O2Na]^+$ *m/z* =513.2765, found *m/z* = 513.2767, Δ*m/z*= 0.4 ppm

^{1}H NMR (600 MHz, $CDCl_3$) δ 8.50 (d, J = 9.3 Hz, 1H), 8.31 (d, J = 8.0 Hz, 1H), 8.19 – 8.15 (m, 3H), 8.13 (d, J = 9.3 Hz, 1H), 8.08 – 8.04 (m, 3H), 8.00 (t, J = 7.6 Hz, 1H), 7.62 (d, J = 8.5 Hz, 2H), 7.31 (d, J = 16.0 Hz, 1H), 6.97 (d, J = 8.5 Hz, 2H), 4.02 (t, J = 6.5 Hz, 2H), 3.65 (t, J = 6.7 Hz, 2H), 1.86 – 1.78 (m, 2H), 1.52 – 1.23 (m, 16H).

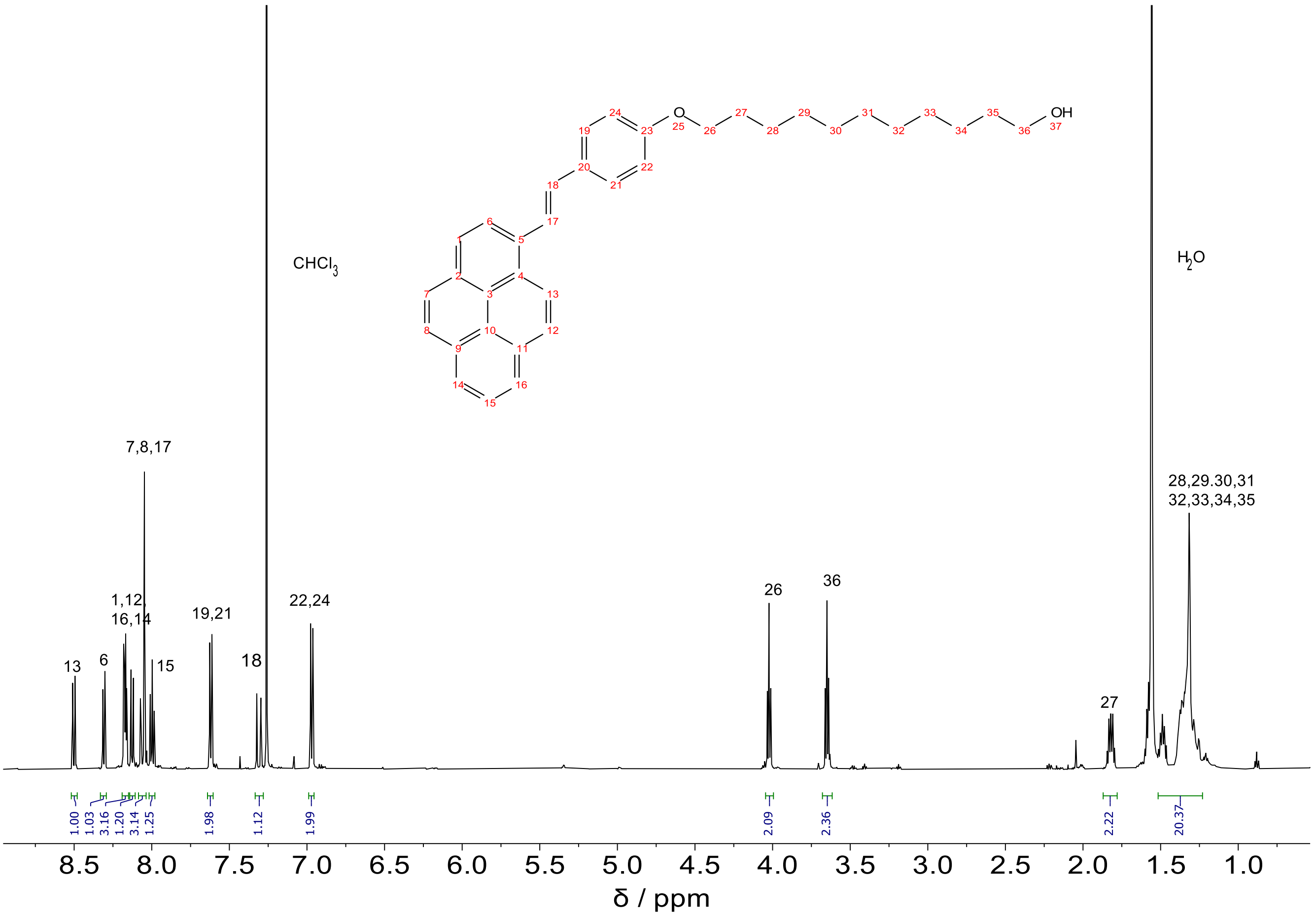

*Figure S 18. ^{1}H NMR spectrum of **SC3** acquired in $CDCl_3$.*

4.4. SC4

Diacid **S4** (184 mg, 0.69 mmol, 1 eq), dry dichloromethane (5 mL), oxalylchloride (0.24 mL, 2.75 mmol, 4 eq) were added in an ice cooled Schlenk line flask that connected to Ar gas. 1 drop of dimethylformamide was added to the flask. The mixture was then kept stirring under Ar for 3h. After that, the reaction mixture was brought to room temperature, and the solvent and excess oxalylchloride were removed by vacuum. To the reaction flask, **SC3** (1.35g, 2.75 mmol, 5 eq), pyridine (0.28 mL, 3.43 mmol, 5 eq), dry THF (50 mL) were added. The mixture was stirred under Ar for 16 h. To completion, the reaction mixture was concentrated under vacuum, followed by dissolving in dichloromethane to extract with HCl 1N (40 mL x3). The organic phase was then concentrated under reduced pressure and purified by a flash column chromatography with cyclohexane/DCM (98%/2% to 0%/100%) as eluent. The obtained solid was the mixture of **SC4** and a small content of **SC3**. Multiple-time precipitation in methanol removed **SC3**, affording yellow solid **SC4**.

Yield: 300 mg, 0.25 mmol, 35 %.

HRMS: $[C83H86NaO6S]^+$ *m/z* 1233.6038, found *m/z* 1233.6039, Δ*m/z*= 0.1 ppm

^{1}H NMR (600 MHz, $CDCl_3$) δ 8.48 (d, J = 9.2 Hz, 2H), 8.28 (d, J = 8.0 Hz, 2H), 8.18 – 8.13 (m, 6H), 8.10 (d, J = 9.3 Hz, 2H), 8.06 – 8.01 (m, 6H), 7.98 (t, J = 7.8 Hz, 2H), 7.60 (d, J = 8.7 Hz, 4H), 7.40 (d, J = 7.8 Hz, 2H), 7.35 – 7.21 (m, 5H), 6.95 (d, J = 8.3 Hz, 4H), 6.42 (s, 1H), 5.97 (s, 1H), 5.29 (s, 1H), 4.15 – 4.05 (m, 4H), 4.03 – 3.95 (m, 4H), 3.17 (q, J = 7.3 Hz, 1H), 1.85 – 1.76 (m, 4H), 1.70 – 1.22 (m, 35H).

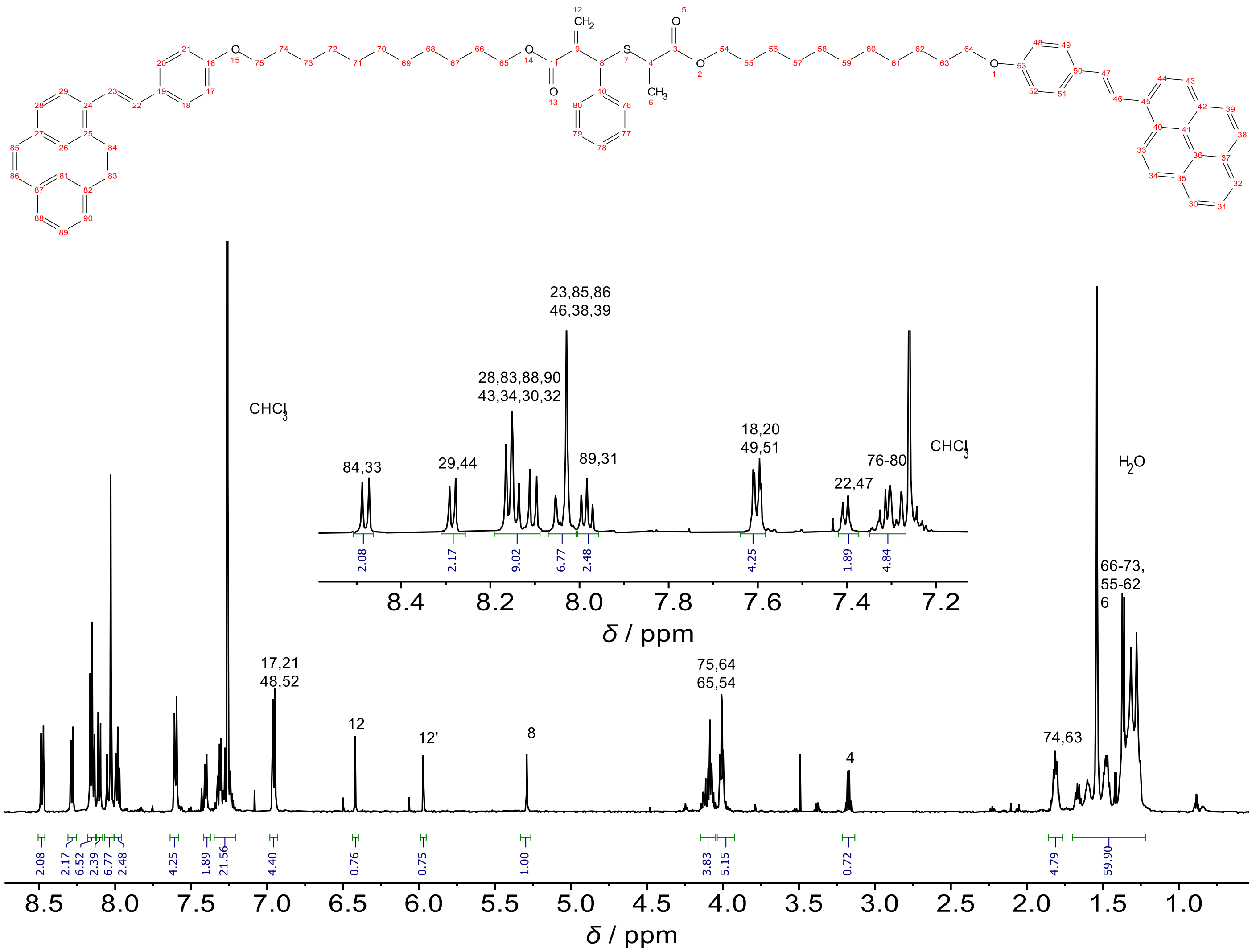

*Figure S 19. ^{1}H NMR spectrum of **SC4** acquired in $CDCl_3$.*

4.5. SC5

In order to prevent intermolecular cycloaddition reaction, the cyclisation reaction of linear monomer **SC4** was conducted at dilute concentration. 10 mL solution of **SC4** 0.5 mg/mL in toluene was degassed with Ar for 20 minutes. Then the solution was irradiated with a LED 465 nm (10W, 1.05 A). The reaction was monitored by SEC at isosbestic point of the photo cycloaddition reaction 355 nm. It was found that the reaction completed after 150 min. The reaction mixture was then concentrated under reduced pressure and freeze dried to remove solvent completely, giving **SC5** as a yellow viscous oil.

Yield: 5 mg, 100 %.

HRMS: $[C83H86NaO6S]^+$ *m/z* 1233.6038, found *m/z* 1233.6029, Δ*m/z*= -0.8 ppm

^{1}H NMR (600 MHz, $CDCl_3$) δ 8.35 (d, J = 9.3 Hz, 2H), 8.11 – 7.97 (m, 8H), 7.93 – 7.80 (m, 8H), 7.43 – 7.23 (m, 5H), 7.21 (d, J = 8.4 Hz, 4H), 6.78 (d, J = 8.4 Hz, 4H), 6.43 (s, 1H), 5.98 (s, 1H), 5.83 – 5.79 (m, 2H), 5.30 (s, 1H), 4.84 – 4.80 (m, 2H), 4.17 – 3.86 (m, 8H), 3.17 (q, J = 7.1 Hz, 1H), 1.82 – 1.13 (m, 39H).

^{1}H,^{1}H GCOSY (600MHz / 600 MHz, $CDCl^3$): δ ^{1}H / δ ^{1}H = 8.51 / 8.28, 8.35 / 7.89, 7.21 / 6.78, 5.81 / 4.82, 4.99 / 4.20.

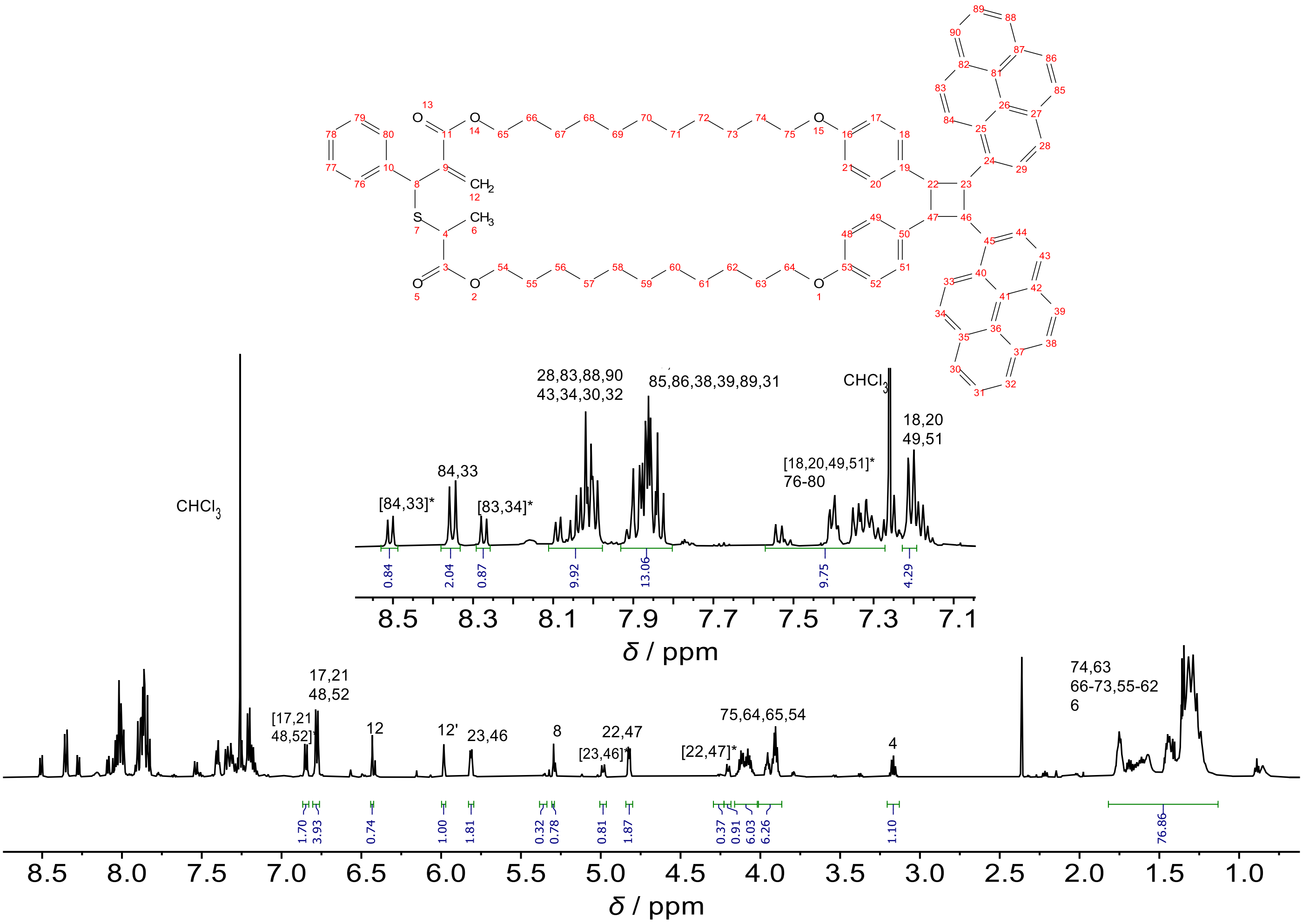

*Figure S 20. ^{1}H NMR spectrum of cyclic monomer **SC5** acquired in $CDCl_3$.*

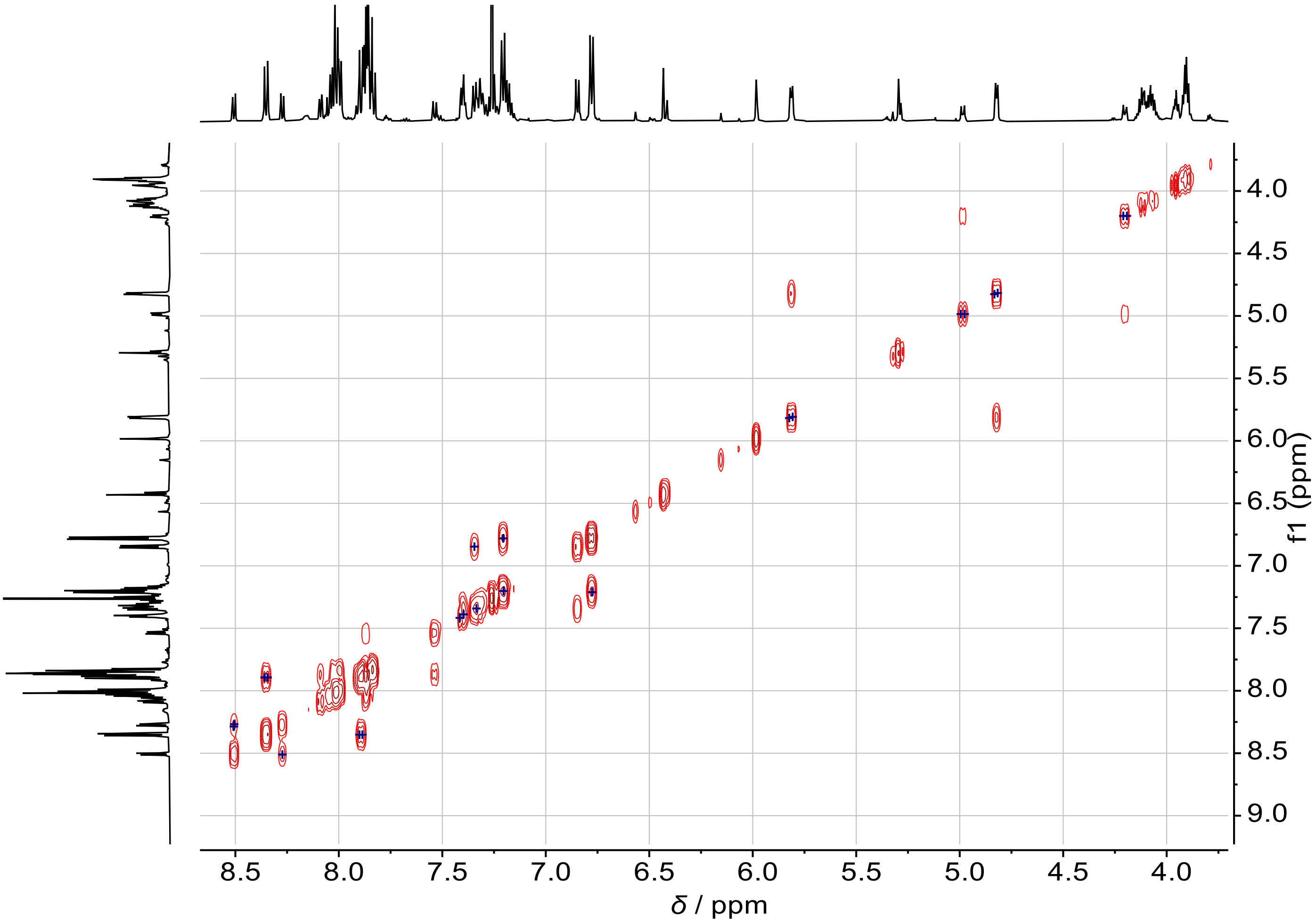

*Figure S 21. ^{1}H -^{1}H COSY of cyclic monomer **SC5** acquired in $CDCl_3$.*

5. Synthesis of polymer

Materials

Azobisisobutyronitrile (Sigman-Aldrich-Merck, recrystalized), Zinc acetate (Sigma Aldrich), free base (hTPP) (Sigma Aldrich), DMF (Sigma-Aldrich, > 99 %), DMSO (Sigma-Aldrich, > 99 %), N,N-Dimethylacrylamide (DMA) (Sigma Aldrich, after passing through a short plug of basic alumina), 5,10,15,20-Tetrakis(4-hydroxyphenyl)-21H,23H-porphine zinc (ZnhTPP) (synthesized according to [10]), 2-(((ethylthio)carbonothioyl) thio) propanoic acid (synthesized according to literature[11]).

5.1. Synthesis of copolymer of styrylpyrene monomer and DMA

Synthesis of copolymer P1

N,N- Dimethylacrylamide (DMA) (25.76 µL, 250 µmol, 2500 eq), azobisisobutyronitrile (AIBN) (6.57 µL from stock solution 5 mg/mL in DMF, 0.2 µmol, 2 eq), 2-(((ethylthio)carbonothioyl)thio)propanoic acid (CTA) (11.7 µL from stock solution 15 mg/mL in DMF, 0.5 µmol, 5 eq), cyclic monomer SC5 (6.1 mg, 5 µmol, 50 eq), and DMF (46 mL) were charged to a glass vial. Subsequently, the vial was capped and degassed with Argon gas for 8 minutes. The vial was then heat up at 80 ºC for 22 h. To completion, the vial was opened to exposure

to air for quenching polymerisation. The precipitation of reaction mixture with diethyl ether afforded a slight yellow polymer (Mn 20 kg.mol^{-1}, Đ 1.6).

A control sample which is the polymerization without cyclic polymer was also carried out, yielding a homopolymer PDMA (Mn 19 kg.mol^{-1}, Đ 1.3, Figure S20 below).

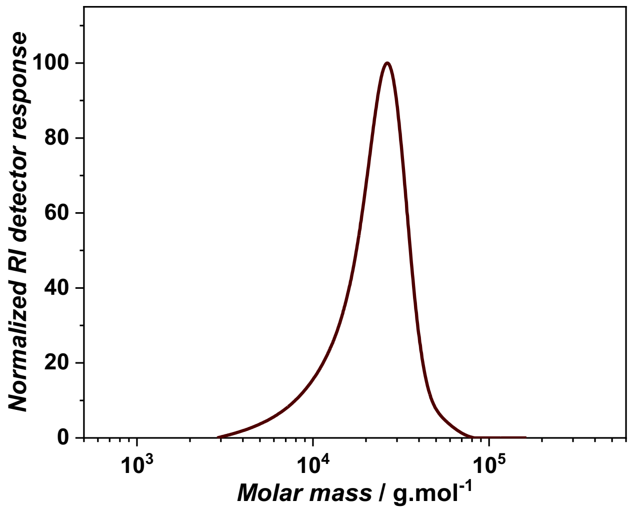

Figure S 22. SEC chromatogram of homopolymer PDMA without styrylpyrene cyclic monomer.

Kinetic study of copolymerisation of SC5 and DMA

N,N- Dimethylacrylamide (DMA) (161.78 µL, 1570 µmol, 2500 eq), azobisisobutyronitrile (AIBN) (41.25 µL from stock solution 5 mg/mL in DMF, 1.256 µmol, 2 eq), 2-(((ethylthio)carbonothioyl)thio)propanoic acid (CTA) (73.39 µL from stock solution 15 mg/mL in DMF, 3.14 µmol, 5 eq), cyclic monomer SC5 (38.0 mg, 31.14 µmol, 50 eq), and DMF (288.80 mL) were mixed and divided into equal parts of 80 µL in glass vials. Subsequently, these vials were capped and degassed with Argon gas for 8 minutes. The vial was then heated up at 70 °C for different time. To conduct a measurement, the vial was opened to exposure to air for quenching polymerisation.

5.2. Synthesis of copolymers and dicblock copolymers of coumarin based C3 monomer and DMA

Experimental setup

The polymerization setup adapted from literature [10] was illustrated in Figure S21 below. Briefly, in a photobox, the well plate of polymerization solution was placed on the top of the LED plate (λ_{max} = 572 nm) as the light source to catalyse the polymerization. The LED plate with the heat sink attached was put in contact with a metal surface (*e.g.* a heat plate without heating activation) to facilitate heat transfer during polymerization process, preventing overheating the LED plate and well plate. A fan was used to assist the heat reduction of the polymerization system. The LED plate was connected to a power supply which can be tailored to desired current and voltage. The current used in experiment was 0.05A/row of 8 LED bulbs. The polymerization time can be set up by a timer which connects power supply with the electricity power.

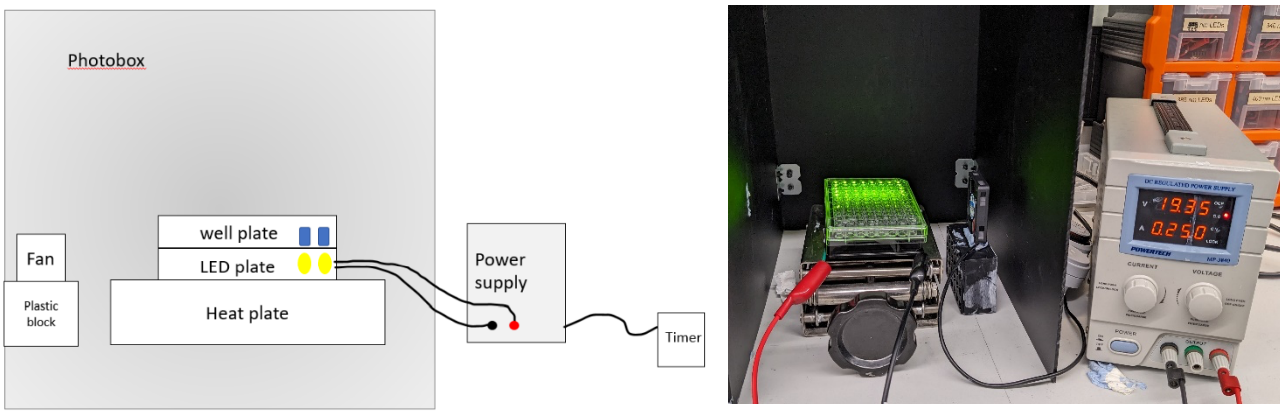

Figure S 23. Illustrative representation of experimental setup of PET-RAFT polymerisation experiment (left) and picture of actual setup (right)

Copolymer synthesis

Dimethyl acrylamide (DMA) monomer (5.2 µL, 50.4 µmol, 150 eq), ZnhTPP (3.36 µL of 0.01M in DMSO solution, 0.034 µmol, 0.1 eq), 2-(((ethylthio)carbonothioyl) thio) propanoic acid (13.3 µL of 5.3 mg/mL in DMSO solution, 0.34 µmol, 1 eq) were added in a well in the well plate. Cyclic monomer C3 (100 mg/mL DMSO) were added in the well to pre-determined feed ratio. After that DMSO was added to the total volume of polymerisation mixture of 100 µL. The polymerization solution was irradiated with an LED plate for 20h. The polymer product was precipitated out of dimethyl ether, and dried under reduced pressure.

Block copolymer synthesis

Dimethyl acrylamide (DMA) monomer (5.2 µL, 50.4 µmol, 100 eq), ZnhTPP (1.51 µL of 0.01M in DMSO solution, 0.015 µmol, 0.03 eq), 2-(((ethylthio)carbonothioyl) thio) propanoic acid (20.0 µL of 5.3 mg/mL in DMSO solution, 0.5 µmol, 1 eq) and DMSO (73.3 µL) were added in a well in the well plate. Total volume of polymerisation mixture was 100 µL. The polymerization solution was irradiated with an LED plate for 20h.

After that, 10 µL of the polymerization was taken out for SEC measurement. Thus, the remaining amount of CTA (PDMA with RAFT end group) and ZnhTPP in the well plate was 90% of the initial CTA and ZnhTPP amount before beginning the first block polymerization. The fresh DMA monomer (2.34 µL, 22.7 µmol) was then added to the desired ratio of [DMA]/[CTA]=50. Fresh ZnhTPP (1.51 µL of 0.01M in DMSO solution, 0.015 µmol) was added to desired ratio of [ZnhTPP]/[CTA]= 6 %. Cyclic monomer **C3** (8.12 µL of 100 mg/mL in DMSO solution, 0.91 µmol, 2 eq) was finally added to the mixture, [**C3**]/[CTA]=2. Subsequently, the well plate was submitted to PET RAFT polymerization for 20 h. The polymer was precipitated out of diethyl ether and dried under reduced pressure, giving a slight green polymer (Mn 18.5 kg.mol^{-1}, Đ 1.3).

A control sample which is the polymerization without cyclic polymer and desired ratio of [DMA]/[CTA]=100 in second block was also carried out, yielding a homopolymer PDMA (Mn 22 kg.mol^{-1}, Đ 1.2, Figure S22 below).

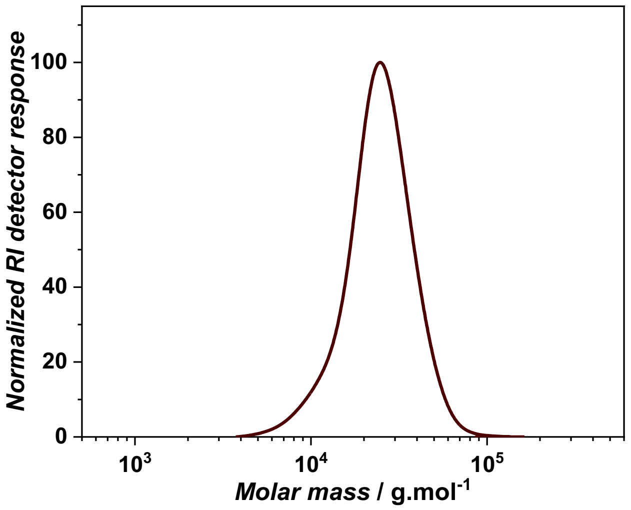

Figure S 24. SEC chromatogram of homopolymer PDMA without coumarin cyclic monomer.

5.3. Synthesis of triblock polymer

Diblock polymer containing coumarin dimer in the backbone was synthesized according to the procedure above. The diblock polymer after workup was dried with Nitrogen gas for 1h to remove diethyl ether solvent. After that, the polymer was dissolved with 70 µL DMF in a glass vial. An aliquot of 3 µL of this solution was used to measure SEC. The remaining 67 µL was used as macro-CTA for chain extension with DMA and **SC5** cyclic monomer. With the assumption that all of RAFT agent attended polymerisation reaction to result in polymer with RAFT end group and all polymer was precipitated in diethyl ether, the amount of CTA in this 67 µL solution was 0.43 µmol (5.0 eq). To this solution, DMA (21.9 µL, 213 µmol, 2500.0 eq), AIBN (1.4 µL from stock solution 5 mg/mL in DMF, 0.043 µmol, 0.5 eq), styrylpyrene monomer **SC5** (5.2 mg, 4.3 µmol, 50.0 eq) were added. Subsequently, the vial was capped and degassed with Argon for 8 minutes. The vial was then kept at 80°C for 22 h. After that, the vial was opened to air to stop the polymerisation. The mixture was precipitated with diethyl ether, followed by washing with water to remove unreacted diblock. Finally, the obtained polymer was dried under vacuum, giving a yellow polymer (Mn 40 kg.mol^{-1}, Đ 1.9).

6. Calculation and estimation

6.1. Estimation of copolymer P1

a) Estimation of degree of polymerization (DP)

+) Derived from averaged molar weight of polymer:

M_n=DP x $M_{monomer}$

M_{DMA}= 99 g.mol^{-1}, M_{SC5}=1212 g.mol^{-1}

Mn_{P1}= 20 000 g.mol^{-1}

$DP_{DMA} \times M_{DMA}$(g.mol^{-1})+ $DP_{SC5} \times M_{SC5}$(g.mol^{-1})= Mn_{P1} (g.mol^{-1})

➔ $99 \times DP_{DMA} + 1212 \times DP_{SC5} = 20000$ (1)

+) Incorporation ratio of SC5 to DMA is 1.8%

➔ $\frac{DP_{SC5}}{DP_{DMA}} = 0.018$ (2)

From (1) and (2), it can be calculated that DP_{DMA}=166, DP_{SC5}=3

Thus, each copolymer **P1** chain contains 166 units of DMA and 3 units of **SC5** in average.

b) Estimation of polymer degradation

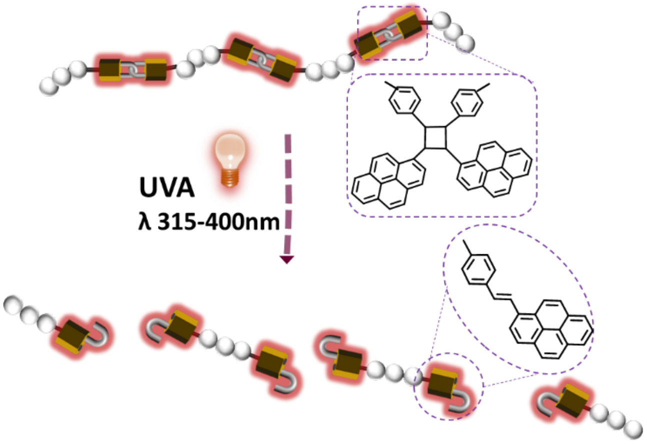

*Figure S 25. Illustrative representation of the degradation of copolymer **P1** DMA-co-**SC5**.*

As illustrated in above Figure, if all 3 styrylpyrene cycloadducts undergo cycloreversion reaction under UVA irradiation, each **P1** chain will disintegrate into 3+1=4 comparable fragments, assuming the even distribution of photoresponsive group in polymer chain. Hence, after complete degradation, Mn of the polymer will be:

$$Mn_{degraded} = \frac{Mn_{P1}}{4} = \frac{20000}{4} = 5000 \quad g.mol^{-1}$$

Thus, if **P1** is fully degraded, Mn should reduce by:

$$Mn\ reduction = \frac{20000-5000}{20000} = 75\ \%$$

6.2. Estimation of diblock copolymer P2

a) Estimation of degree of polymerization

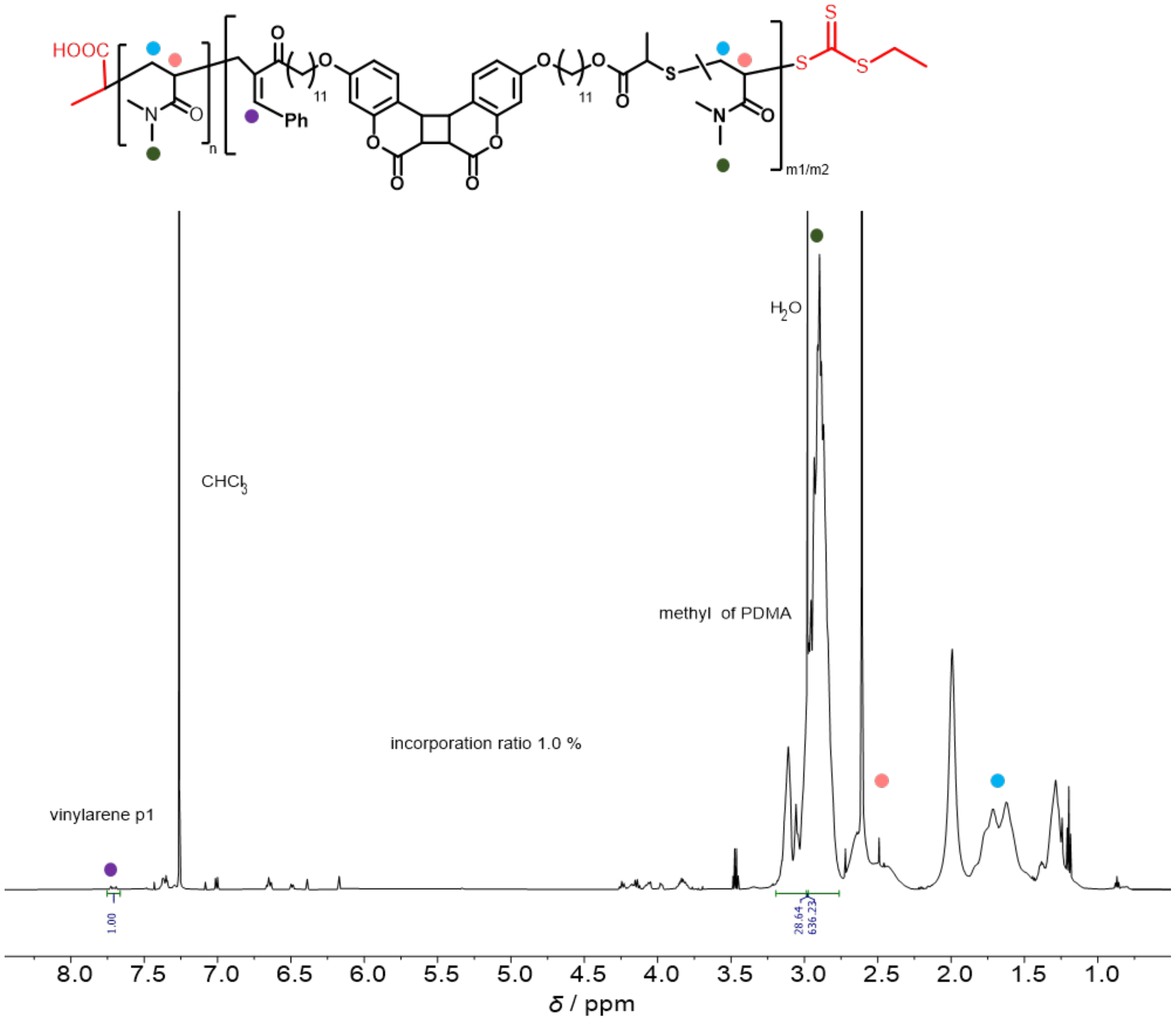

*Figure S 26. ¹H NMR spectra of **P2** (PDMA-b-P(DMA-co-**C3**)) in $CDCl_3$. Integral of dimethyl proton in PDMA (δ= 2.9 ppm) is obtained from subtraction of integral of water protons. The incorporation ratio of coumarin monomer **C3** to DMA is calculated from vinylarene proton of ring-opening product of **C3** and methyl protons of PDMA.*

Derived from Mp of polymer:

$Mp = DP \times M_{monomer}$

M_{DMA}= 99 g.mol^{-1}, M_{C3}= 894 g.mol^{-1}

+) First block PDMA: Mp= 11000 g.mol^{-1} ➔ polymerisation degree of DMA DP = 111

+) In second block, denote DP_{DMA} and DP_{C3} as polymerisation degree of DMA and **C3** then:

$DP_{DMA} \times M_{DMA}(g.mol^{-1}) + DP_{C3} \times M_{C3}(g.mol^{-1}) + Mp_{\text{first block}}\ (g.mol^{-1}) = Mp_{P2}\ (g.mol^{-1})$

➔ $99 \times DP_{DMA} + 894 \times DP_{C3} + 11000 = 23000$ (3)

Incorporation ratio of **C3** to DMA in **P2** is 1.0%: $\frac{DP_{C3}}{DP_{DMA}+111} = 0.01$ (4)

from (3) and (4), it can be calculated that DP_{DMA}=103 and DP_{C3}=2.

Thus, **P2** is estimated to consist of one block of PDMA (111 units of DMA) and one block of copolymer of DMA (103 units) and coumarin monomer **C3** (2 units)

b) Estimation of polymer degradation

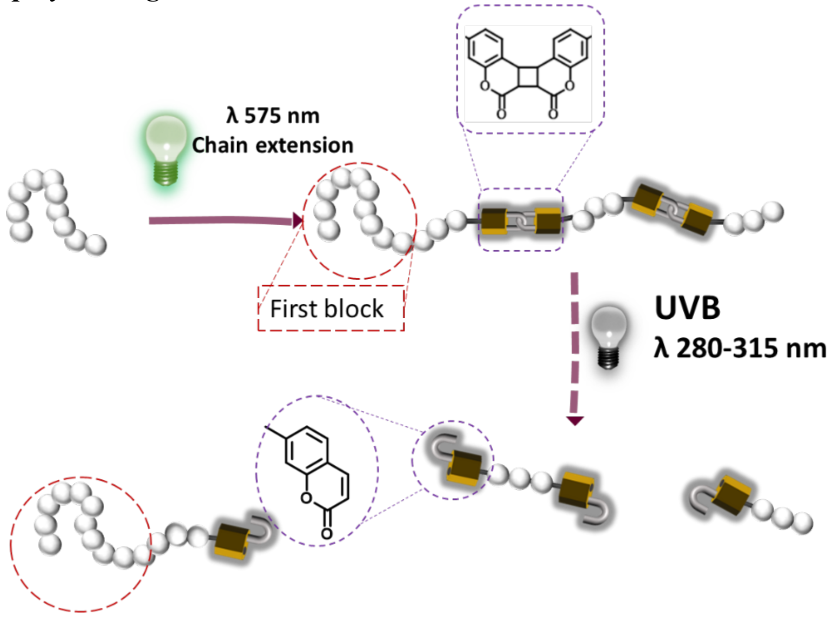

*Figure S 27. Illustrative representation of the degradation of diblock copolymer **P2** PDMA-b-(DMA-co-**C3**)*

Based on Mp of polymer, estimated structure of diblock **P2**: DMA_{111}-*b*-(DMA_{103}-*co*-$\mathbf{C3}_2$). As illustrated in above Figure, if all 2 coumarin cycloadducts undergo cycloreversion reaction under UVA irradiation, each **P2** chain will disintegrate into 2+1=3 fragments. The largest fragment is the one connected to the nondegradable first block and contains only half of the coumarin cycloadduct monomer. Assuming coumarin cycloadduct distributes evenly in the second block of **P2**, if disregards the first block in largest fragment, number of DMA units is equal for each fragment. Hence if **P2** is fully degraded, Mp of the product (mass of largest fragment) will be:

$$Mp_{degraded} = Mp_{first\ block} + \frac{DP_{DMA}}{3} \times M_{DMA} + \frac{1}{2} \times M_{C3}$$

➔ $Mp_{degraded} = 11{,}000 + \frac{103}{3} \times 99 + \frac{1}{2} \times 894 = 14{,}846\ g.mol^{-1}$

6.3. Calculation and estimation of triblock copolymer P3

a) Calculation of incorporation ratio of photoresponsive groups

+) Incorporation ratio of photoresponsive groups (coumarin and styrylpyrene) is calculated from integral of vinylarene proton in copolymer resulted from ring opening polymerisation of both coumarin monomer **C3** and styrylpyrene monomer **SC5** (δ=7.70 ppm) and integral of 6 proton of DMA (δ= 2.90 ppm).

Incorporation ratio of both photoresponsive groups: $I_{total} = \frac{0.87}{418} \times 6 \times 100 = 1.3\%$

+) Incorporation ratio of styrylpyrene is calculated from integral of 2 protons of cyclobutane ring (δ= 5.81 and 4.99 ppm corresponding to two isomers of **SC5**) and integral of 6 proton of DMA (δ= 2.9 ppm)

➔ incorporation ratio of **SC5** to DMA: $I_{SC5} = \frac{\frac{0.82+0.4}{2}}{418} \times 6 \times 100 = 0.9\%$

+) Incorporation ratio of **C3**: $I_{C3}= I_{total} - I_{SC5}= 0.4\%$

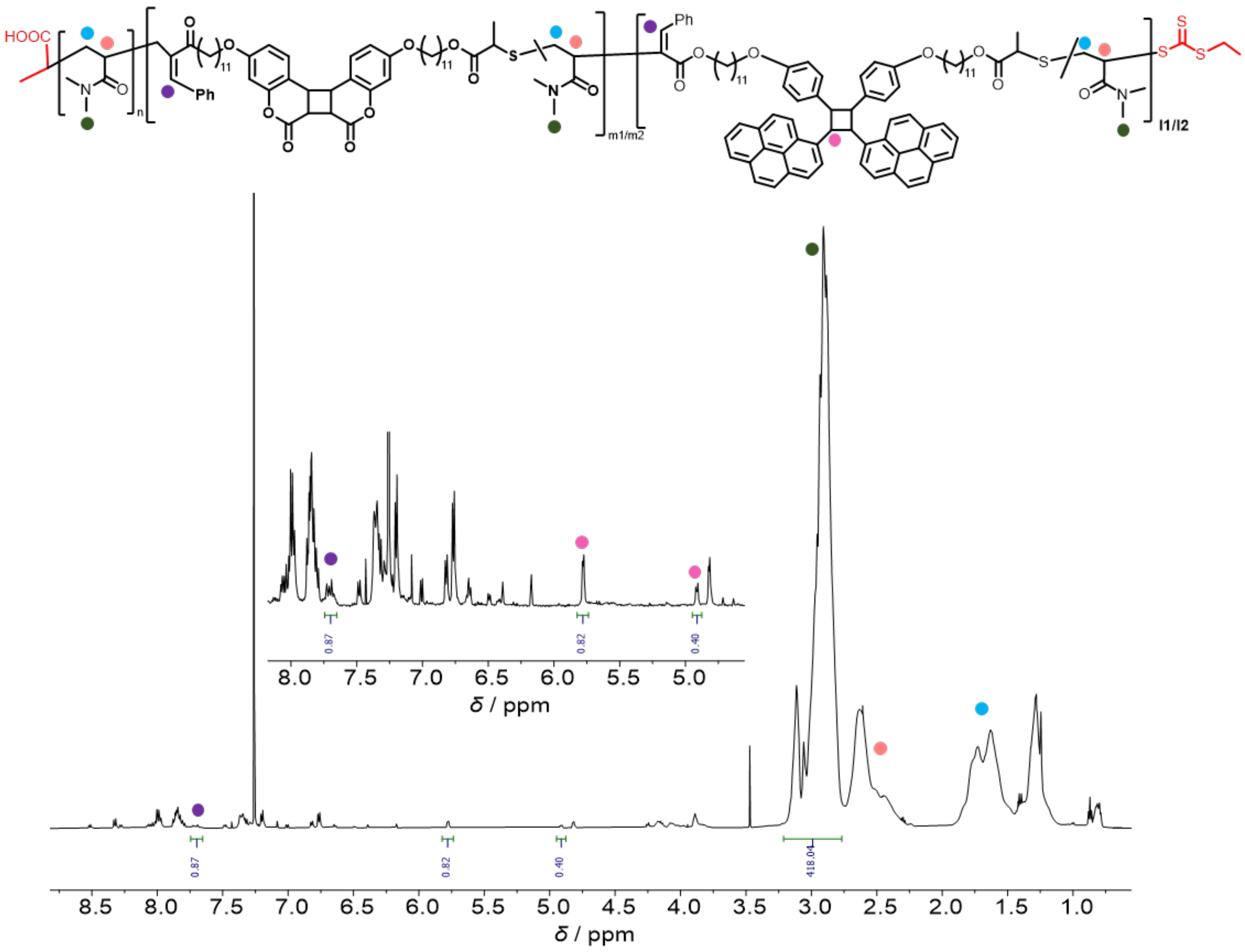

*Figure S 28. ^{1}H NMR of polymer **P3** acquired in $CDCl_3$ with integrals of resonances used for incorporation ratio of photoresponsive groups in **P3**.*

b) *Estimation of degree of polymerization*

Derived from Mp of polymer:

$Mp = DP \times M_{monomer}$

$M_{DMA}= 99$ g.mol^{-1}, $M_{C3}= 894$ g.mol^{-1}

+) First block PDMA: Mp= 10000 g.mol^{-1} ➔ polymerisation degree of DMA DP = 101

+) In second block, denote $DP_{DMA(2)}$ and DP_{C3} as polymerisation degree of DMA and **C3**

then:

$$DP_{DMA(2)} \times M_{DMA}(g.mol^{-1}) + DP_{C3} \times M_{C3}(g.mol^{-1}) + Mp_{\text{first block}}\ (g.mol^{-1}) = Mp_{\text{diblock}}\ (g.mol^{-1})$$

➔ $99 \times DP_{DMA(2)} + 894 \times DP_{C3} + 10000 = 21000$ (5)

Incorporation ratio of **C3** to DMA in **P2** is 1.0%: $\frac{DP_{C3}}{DP_{DMA(2)}+101} = 0.01$ (6)

From (5) and (6), it can be calculated that $DP_{DMA(2)}$=93 and DP_{C3}=2.

+) In third block, denote $DP_{DMA(3)}$ and DP_{SC5} as polymerisation degree of DMA and **SC5**

then:

$$DP_{DMA(3)} \times M_{DMA}(g.mol^{-1}) + DP_{SC5} \times M_{SC5}(g.mol^{-1}) + Mp_{\text{diblock}}\ (g.mol^{-1}) = Mp_{P3}\ (g.mol^{-1})$$

➔ $99 \times DP_{DMA(3)} + 1212 \times DP_{SC5} + 21000 = 78000$ (7)

Incorporation ratio of **SC5** to DMA in **P3** is 0.9%: $\frac{DP_{SC5}}{DP_{DMA(3)}+DP_{DMA(2)}+101} = 0.009$

$\leftrightarrow \frac{DP_{SC5}}{DP_{DMA(3)}+93+101} = 0.009$ (8)

From (7) and (8), it can be calculated that $DP_{DMA(3)}$=499 and DP_{SC5}=9.

In conclusion, triblock is estimated to consist of first block of PDMA (101 units), second block of copolymer of DMA and **C3** (93 units of DMA, 2 units of **C3**) and third block of copolymer of DMA and **SC5** (499 units of DMA, 6 units of **SC5**)

c) Estimation of photodegradation

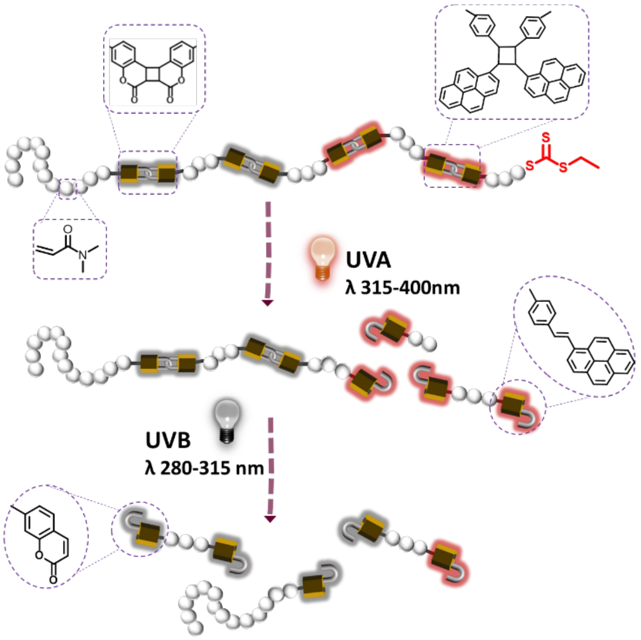

*Figure S 29. Illustrative representation of the degradation of triblock copolymer **P3** PDMA-b-(DMA-co-**C3**)-b-(DMA-co-**SC5**)*

Based on Mp of polymer, estimated structure of triblock **P3**: DMA_{101}-*b*-(DMA_{93}-*co*-$\mathbf{C3}_2$)-*b*-(DMA_{499}-*co*-$\mathbf{SC5}_6$). The estimated Mp of the third block thus is 56673 $g.mol^{-1}$. The full photodegradation of a copolymer equal to the third block would give 7 fragments with mass reduced to 8096 $g.mol^{-1}$ (see section 6.1.b). This fragment is still smaller than the first block of **P3**. Therefore, if the full degradation of **P3** will give the largest fragment similar to that from the full degradation of the diblock. Hence if **P3** is fully degraded, Mp of the product (mass of largest fragment) will be:

$$Mp_{degraded} = Mp_{first\ block} + \frac{DP_{DMA(2)}}{3} \times M_{DMA} + \frac{1}{2} \times M_{C3}$$

➔ $Mp_{degraded} = 10000 + \frac{93}{3} \times 99 + \frac{1}{2} \times 894 = 13{,}516\ g.mol^{-1}$

6.4. Conversion and incorporation ratio in kinetic study of copolymerisation of DMA and SC5

Feed ratio:

Molar ratio of SC5/DMA (denoted as ***F***) in feed is calculated from ^1H NMR of polymerisation mixture. Specifically, SC5 is derived from integral of 8 pyrene aromatic proton resonances at 7.8-7.93 ppm, and DMA is derived from integral of 1 double bond proton resonance at 6.58 ppm (Figure S29). F=1.6%

$$F = \frac{n_{SC5\ in\ polymerisation\ mixture\ at\ 0min}}{n_{DMA\ in\ polymerisation\ mixture\ at\ 0min}} = \frac{\frac{Integral_{Pyrene}}{8}}{\frac{Integral_{DMA}}{1}} = 1.6\% \qquad (9)$$

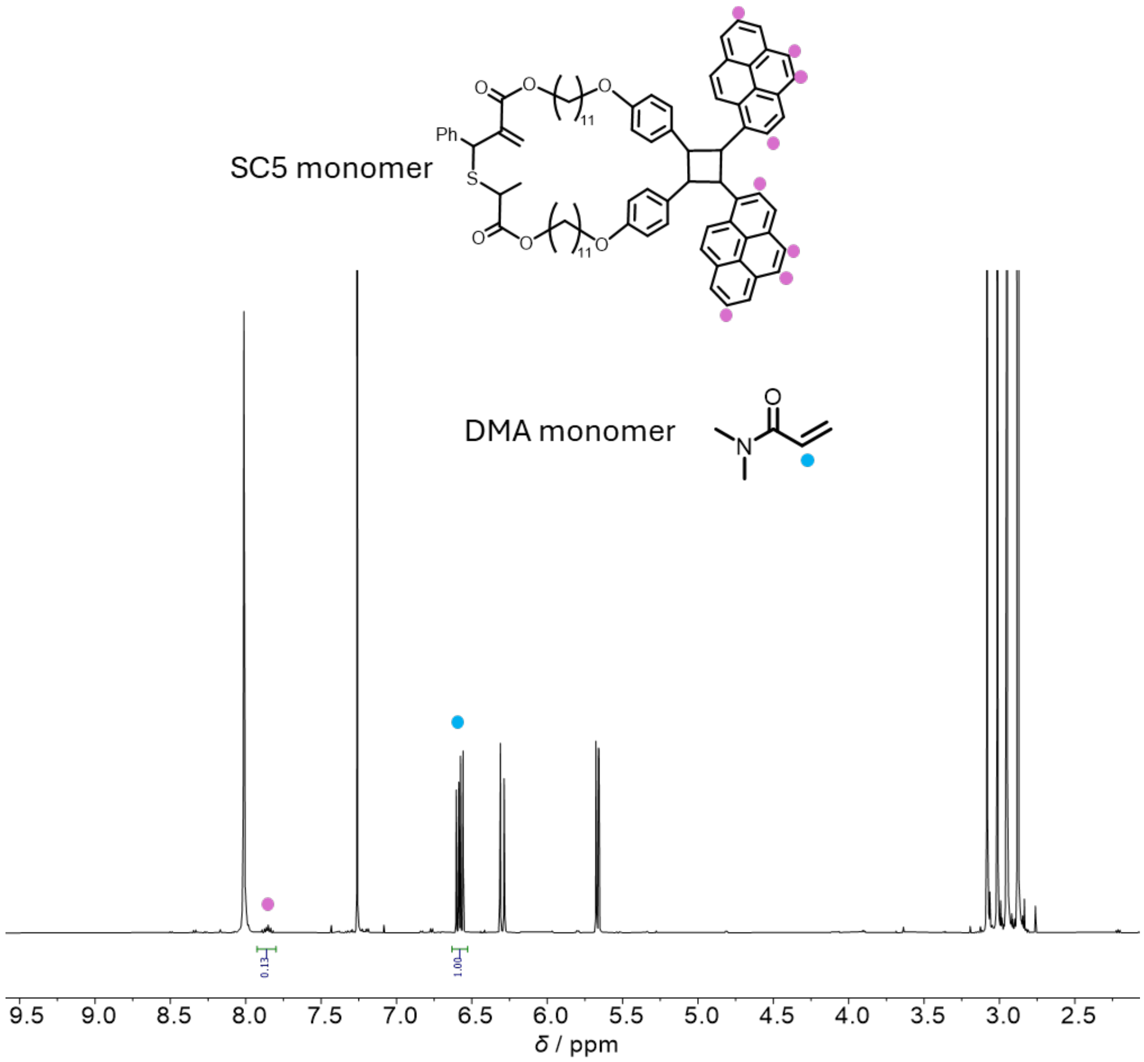

Figure S 30. *¹H NMR spectra of polymerisation mixture at 0 min in kinetic study experiment (acquired in $CDCl_3$).*

Conversion of DMA monomer

Conversion of DMA monomer is calculated from integral of 1 double bond proton of DMA at 6.58 ppm and integral of 6 methyl protons of PDMA (δ= 2.90 ppm) in ¹H NMR of crude polymerisation product. The integral of methyl protons of PDMA is derived from subtraction of integral of DMF solvent and unreacted DMA monomers which are derived from integral of carbonyl proton of DMF and integral of double bond proton of DMA, respectively.

$$C_{DMA} = \frac{n_{DMA\ in\ copolymer}}{n_{DMA\ in\ polymerisation\ mixture\ at\ 0min}} = \frac{n_{DMA\ in\ copolymer}}{n_{DMA\ in\ copolymer} + n_{unreacted\ DMA}} = \frac{\frac{Integral_{PDMA}}{6}}{\frac{Integral_{PDMA}}{6} + Integral_{DMA}}$$

(10)

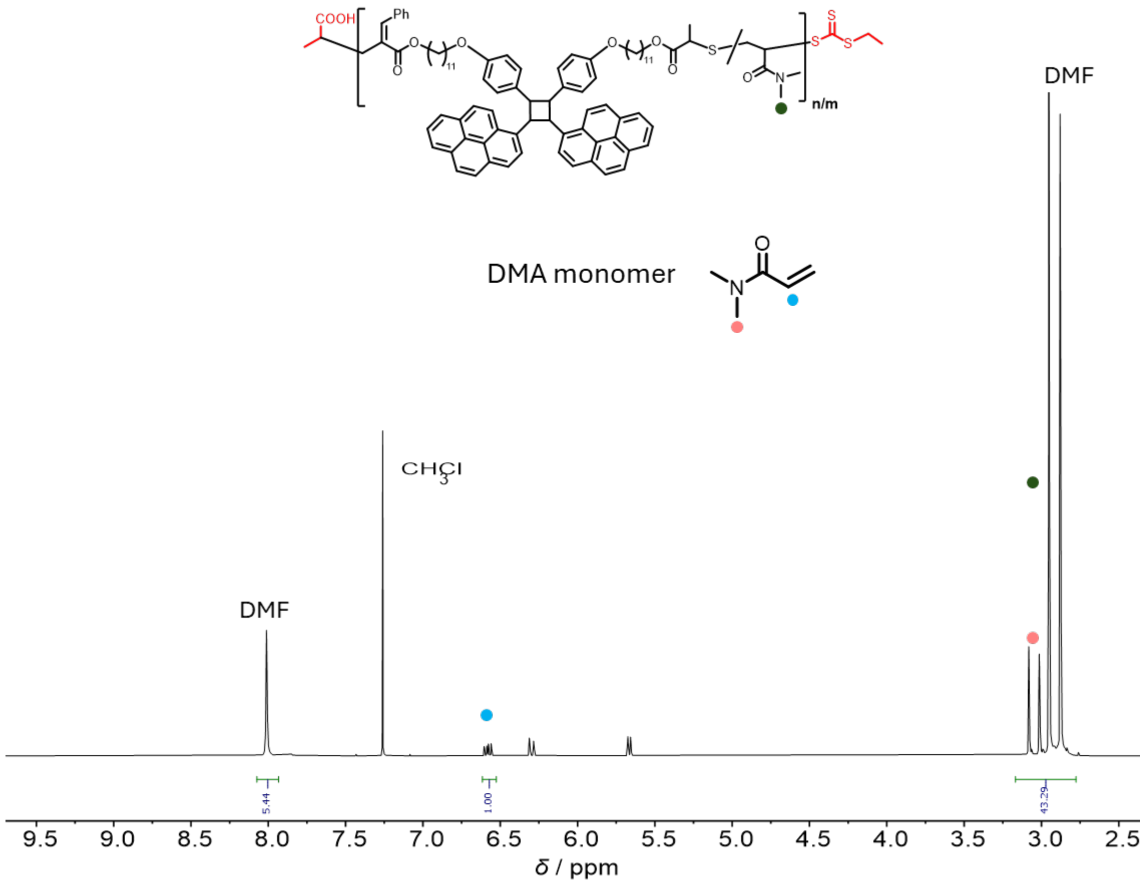

Figure S 31. ^{1}*H NMR spectra of crude polymerisation product obtained from kinetic study experiment at polymerisation time of 60 min (acquired in* $CDCl_3$*).*

Incorporation ratio of copolymer

Incorporation ratio of styrylpyrene cycloadduct groups is calculated from integral of 8 pyrene aromatic protons at 7.8-7.93 ppm and integral of 6 protons of PDMA (δ= 2.90 ppm) in the ^{1}H NMR of the purified copolymer.

$$I = \frac{n_{SC5\ in\ copolymer}}{n_{DMA\ in\ copolymer}} = \frac{\frac{Integral_{Pyrene}}{8}}{\frac{Integral_{PDMA}}{6}} \qquad (11)$$

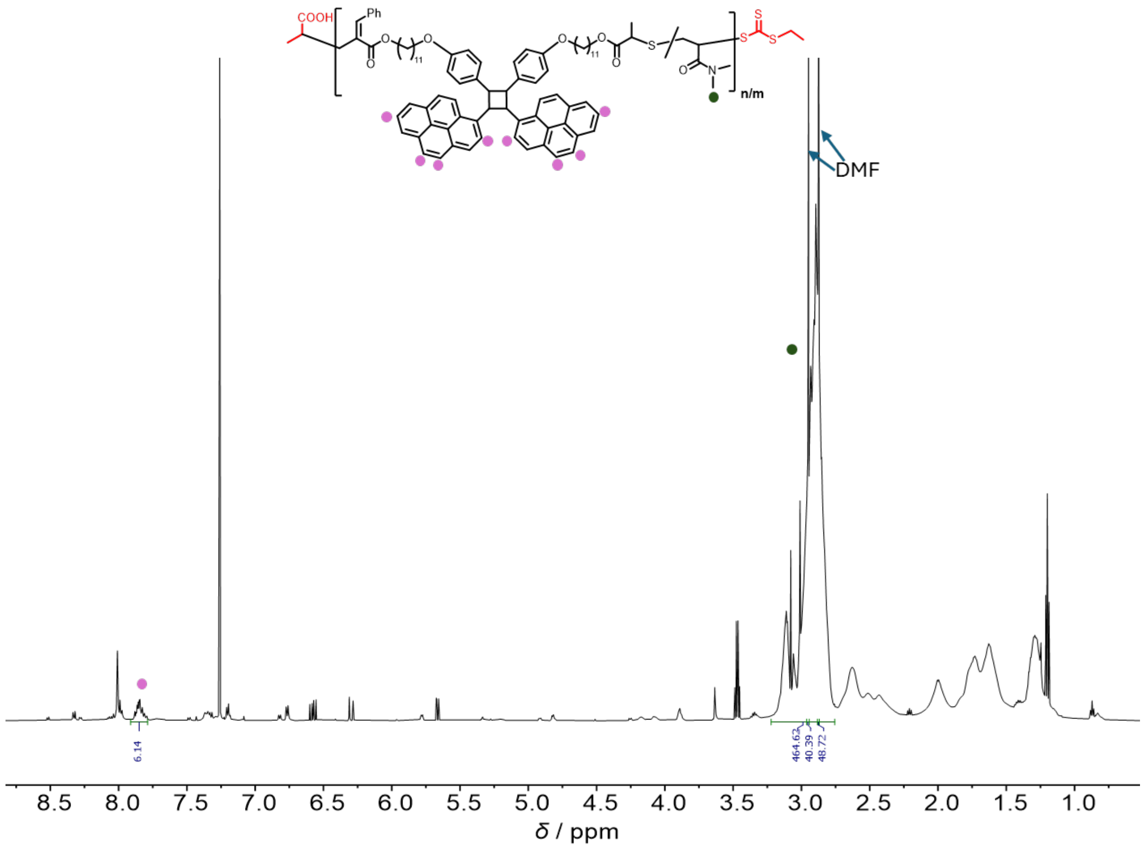

Figure S 32. [1]*H NMR spectra of purified copolymer obtained from kinetic study experiment at polymerisation time of 60 min (acquired in* $CDCl_3$*). Integral of dimethyl proton in PDMA (δ= 2.9 ppm) is obtained from subtraction of integral of DMF protons.*

Conversion of SC5 monomer

Conversion of SC5 (C_{SC5}) is calculated from conversion of DMA(C_{DMA}), feed ratio (F) incorporation ratio of SC5 to DMA in copolymer (I) as follows:

+) from equation (9):

$$n_{SC5\ in\ polymerisation\ mixture\ at\ 0min} = F \times n_{DMA\ in\ polymerisation\ mixture\ at\ 0min} \quad (12)$$

+) from equation (11):

$$n_{SC5\ in\ copolymer} = I \times n_{DMA\ in\ copolymer} \quad (13)$$

+) From equations (12) and (13), conversion of SC5 monomer:

$$C_{SC5} = \frac{n_{SC5\ in\ copolymer}}{n_{SC5\ in\ polymerisation\ mixture\ at\ 0min}} = \frac{I \times n_{DMA\ in\ copolymer}}{F \times n_{DMA\ in\ polymerisation\ mixture\ at\ 0min}}$$

$$C_{SC5} = \frac{I}{F} \times \frac{n_{DMA\ in\ copolymer}}{n_{DMA\ in\ polymerisation\ mixture\ at\ 0min}}$$

$$C_{SC5} = \frac{I}{F} \times C_{DMA} \qquad (14)$$

7. SANS APPENDIX

In the elastic SANS experiment, the neutrons with a wavelength λ scatter from the sample without changing energy. The incident neutrons on the sample have a momentum $\vec{k_\iota}$ and the neutrons scattered by the sample have a momentum $\vec{k_s}$. The angle between the two vectors is the scattering angle, 2θ.

SANS data are acquired as a function of the momentum transfer:

$$\vec{q} = \vec{k_s} - \vec{k_\iota},$$

where the magnitude of the momentum transfer is defined by: $q = \frac{4\pi}{\lambda}\sin(\theta)$.

Initial model-independent analysis has been performed using Guinier approximation and the power-law fit of the intermediate range of the data.

The middle-range q fits are all in the range of the q^{-2}, indicating scattering of a Gaussian polymer.

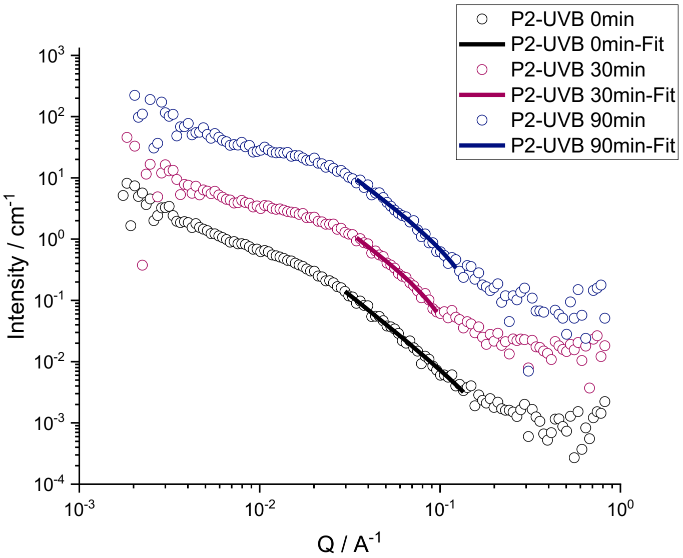

*Figure S 33. Scattering intensity, I(q) plotted versus momentum transfer q obtained from SANS experiments of diblock polymer **P2** dispersion in water irradiated with UVB light for 0 min, 30 min and 90 min and their fit performed by SasView with model Power-Law for the middle-range range of the data. Note that the data and fit of **P2** irradiated with UVB 30 min and 90 min are offset for clarity by 10 and 100, respectively.*

Radius of gyration has R_g been estimated using the Guinier approach and calculated directly from a distance distribution function, $p(r)$.

The Guinier approximation allows to estimate the R_g from the low q part of the $I(q)$ as following:

$I(q) = I(0)\exp\left(-\frac{R_g^2}{3}q^2\right)$, $qR_g < 1$ for compact particles.

One shall be careful selecting the q-range, do not take into account possible aggregation or concentration effects. The polymers solutions studies here are dilute, hence there is no structural factor to be taken into consideration. The aggregation effect, however, shows itself on the data.

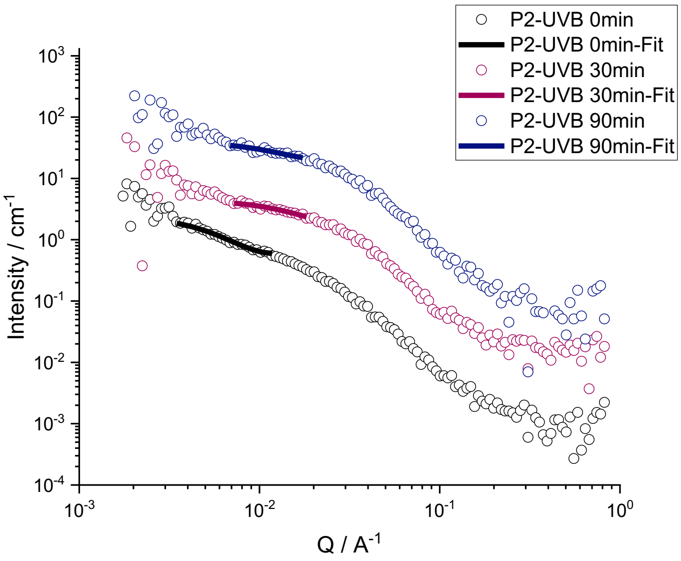

*Figure S 34. Scattering intensity, I(q) plotted versus momentum transfer q obtained from SANS experiments of diblock polymer **P2** dispersion in water irradiated with UVB light for 0 min, 30 min and 90 min and their Guinier approximation fit performed by SasView in the low q-range. Note that the data and fit of **P2** irradiated with UVB 30 min and 90 min are offset for clarity by 10 and 100, respectively.*

The relation between the scattering intensity and the density distribution inside a particle is given by the Fourier transformation:

$$I(q) = 4\pi \int_0^\infty p(r)\frac{\sin(qr)}{qr}dr$$

Where the distribution of interatomic distances, p(r), is calculated as $p(r) = \gamma(r) \cdot r^2$ where γ(r) is a spherically averaged autocorrelation function of the excess scattering density. The distribution becomes zero at r = 0 and r = D_{max}, where D_{max}, is the maximum dimension of a particle (diameter for a case of a sphere).

$$p(r) = \frac{r^2}{2\pi^2}\int_0^\infty q^2 I(q)\frac{\sin qr}{qr}\,\mathrm{d}q \quad [1]$$

R_g can be calculated from $p(r)$ as following:

$$R_g^2 = \frac{\int_V p(r)r^2 dr}{\int_V p(r)dr} \quad [2]$$

For a sphere, the radius R of it can be calculated from the R_g values as following:

$$R_g^2 = \frac{3}{5}R^2 \quad [3]$$

Below are results calculated using Primus from ATSAS software package[6], assuming that the solutions are monodisperse, i.e. all particles have equal shape. This requirement is strong, considering possible presence of aggregation and potential unevenness in the shape considering polymers' crown of the particles. Here we are using $p(r)$ to quantitively compare the data, to describe general tendency of the structural changes observed in

the samples, i.e. position of its maximum and approximate value of the maximum dimension D_{max} of the particles.

Figure S30 shows normalised p(r) for all three data sets:

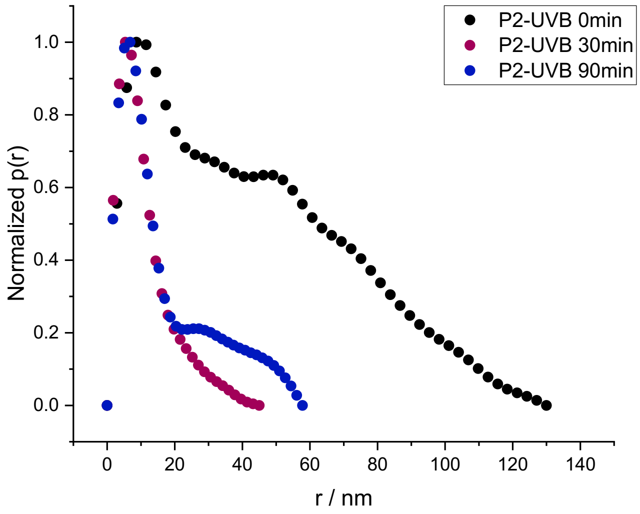

Figure S 35. Normalised p(r) for all three data sets.

Table S3 below summarise estimated values of the R_g and diameter for the three data sets:

Table S 3. Summary of estimated values of the Rg and radii for the three data sets.

	R_g, nm<br>Guinier approximation	R_g, nm<br>see eq [2]	Average particle diameter D, nm,<br>see eq [3]	*D_{max} ($p(r)$), nm<br>see eq [1]
P2-UVB 0 min	33	36	~100	120-140
P2-UVB 30 min	12	9	~25-30	40-50
P2-UVB 90 min	15	15	~40	50-60

*D_{max} for these data sets is very sensitive to the choice of the lowest feasible value of q, hence the solution is not always stable, therefore there is a reasonable range presented for the D_{max}.

Next, the scattering intensity of the polymer micelle was modelled by spherical polymer micelle model via Sasview [SasView 5.0.5. http://www.sasview.org/]:

The form factor, $P(q)$, defined as following:

$$P(q) = N^2\beta_s^2\Phi(qr)^2 + N\beta_c^2P_c(q) + 2N^2\beta_s\beta_cS_{sc}(q) + N(N-1)\beta_c^2S_{cc}(q)$$

$$\beta_s = V_{\text{core}}(\rho_{\text{core}} - \rho_{\text{solvent}})$$

$$\beta_c = V_{\text{corona}}(\rho_{\text{corona}} - \rho_{\text{solvent}})$$

where ρ_{core}, ρ_{corona} and $\rho_{solvent}$ are the scattering length densities *sld_core, sld_corona and sld_solvent.*

During the modelling, the following parameters have been fixed: sld_solvent and d_penetration (the polymers chains do not penetrate the core).

The following parameter have been kept close for all three data sets: sld_core, n_densoty and n_aggreg.

The following parameter have been set to a variable range: sld_core and sld_solvent.

The following parameter were let free: scale, background, v_core, v_corona, sld_corona, radius_core (*Rc*) of an individual polymer' head forming the core, and R_g of an individual polymer chains attached to the core.

Table S 4. Structure values of polymer micelle obtained from performing fitting of experimental SANS data with spherical polymer micelle model via SasView.

	**Volume$_{core}$** (nm^3)	**Volume$_{corona}$** (nm^3)	***Rc*** (nm)	***Rg*** (nm)
**UVB 0min**	$2.1*10^3$	$3.6*10^3$	6.73	7.00
**UVB 30min**	$0.6*10^3$	$0.12*10^3$	4.70	3.80
**UVB 90min**	$0.1*10^3$	$0.1*10^3$	4.50	4.20

8. DOSY APPENDIX

Diblock polymer P2

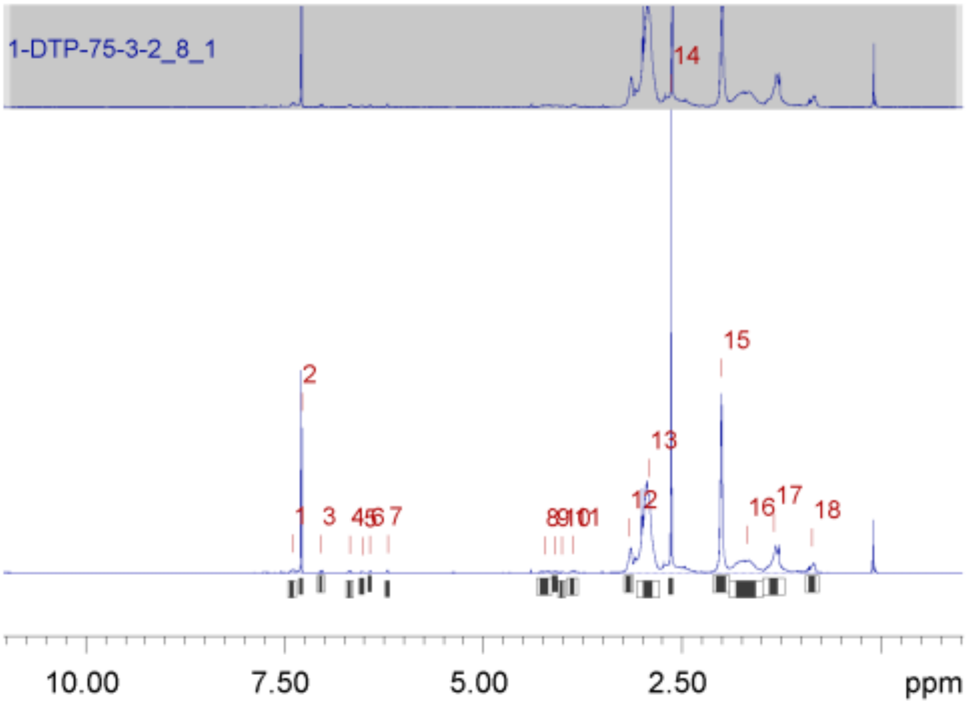

Dosy/Fit

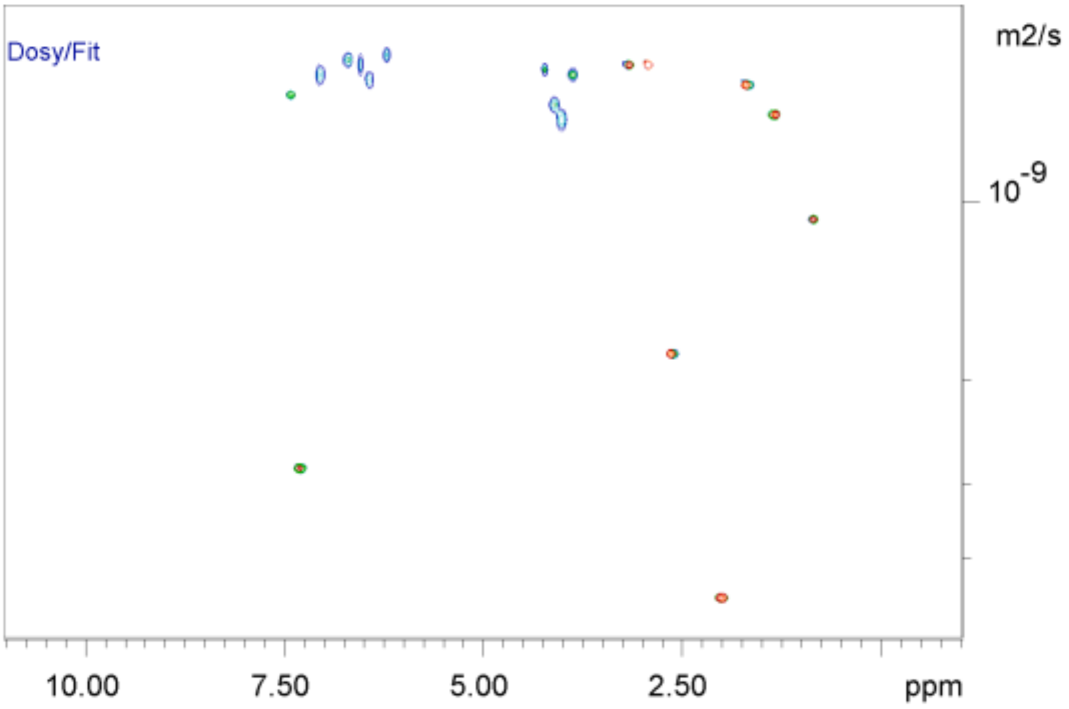

Fitted function:	f (x) = Io * exp (-D * x^2 * gamma^2 * littleDelta^2 (bigDelta-littleDelta/3)* 10^4
used gamma:	26752 rad/(s*Gauss)
used little delta:	0.0026000 s
used big delta:	0.099900 s
used gradient strength:	variable
Random error estimation of data:	RMS per spectrum (or trace/plane)
Systematic error estimation of data:	worst case per peak scenario
Fit parameter Error estimation method:	from fit using calculated y uncertainties
Confidence level:	95%
Used peaks:	peaks from C:/Data/Chemists/FedericaS/1-DTP-75-3-2/8/pdata/1/peaklist1D.xml
Used integrals:	area integral
Used Gradient strength:	all values (including replicates) used

Peak name	F2 [ppm]	Io	error	D [m2/s]	error	fitInfo
1	7.402	4.54e+08	1.578e+06	6.64e-10	5.292e-12	Done
2	7.285	3.10e+09	1.581e+06	2.82e-09	3.287e-12	Done
3	7.037	1.20e+08	1.479e+06	6.11e-10	1.734e-11	Done
4	6.677	1.91e+08	1.476e+06	5.78e-10	1.029e-11	Done
5	6.519	8.57e+07	1.275e+06	5.87e-10	2.013e-11	Done
6	6.422	8.90e+07	1.111e+06	6.20e-10	1.782e-11	Done
7	6.199	1.10e+08	1.199e+06	5.68e-10	1.422e-11	Done
8	4.227	4.07e+08	2.138e+06	6.03e-10	7.288e-12	Done
9	4.093	1.77e+08	1.384e+06	6.79e-10	1.216e-11	Done
10	4.008	1.22e+08	1.564e+06	7.24e-10	2.128e-11	Done
11	3.874	3.53e+08	1.824e+06	6.08e-10	7.231e-12	Done
12	3.168	3.93e+09	1.672e+06	5.88e-10	5.766e-13	Done
13	2.921	3.17e+10	2.592e+06	5.90e-10	1.111e-13	Done
14	2.632	8.55e+09	1.579e+06	1.81e-09	7.606e-13	Done
15	2.007	1.70e+10	3.726e+06	4.63e-09	2.329e-12	Done
16	1.691	9.72e+09	3.203e+06	6.38e-10	4.831e-13	Done
17	1.344	8.18e+09	2.645e+06	7.15e-10	5.297e-13	Done
18	0.861	1.96e+09	2.307e+06	1.08e-09	2.872e-12	Done

Current fit display

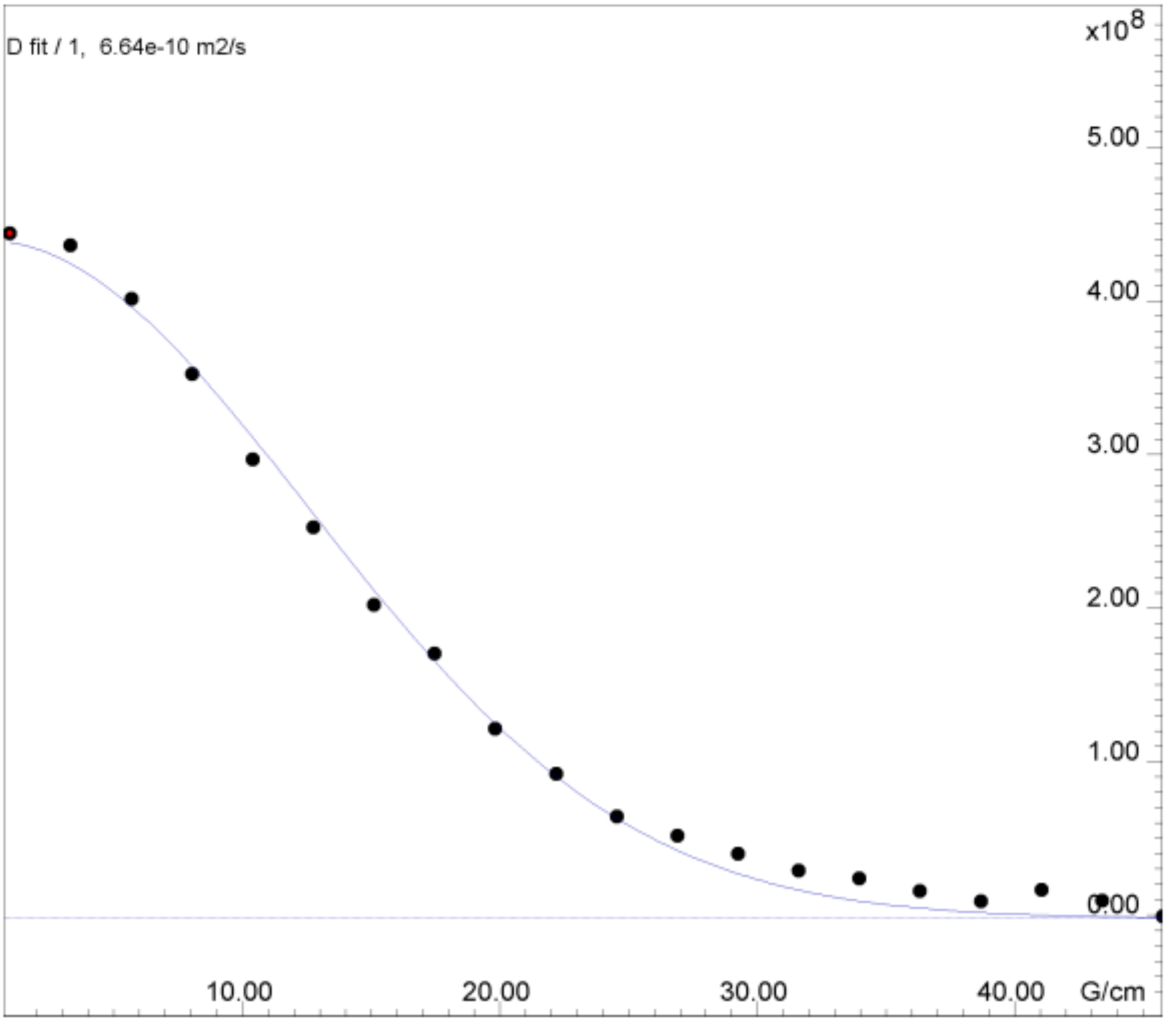

Triblock polymer P3

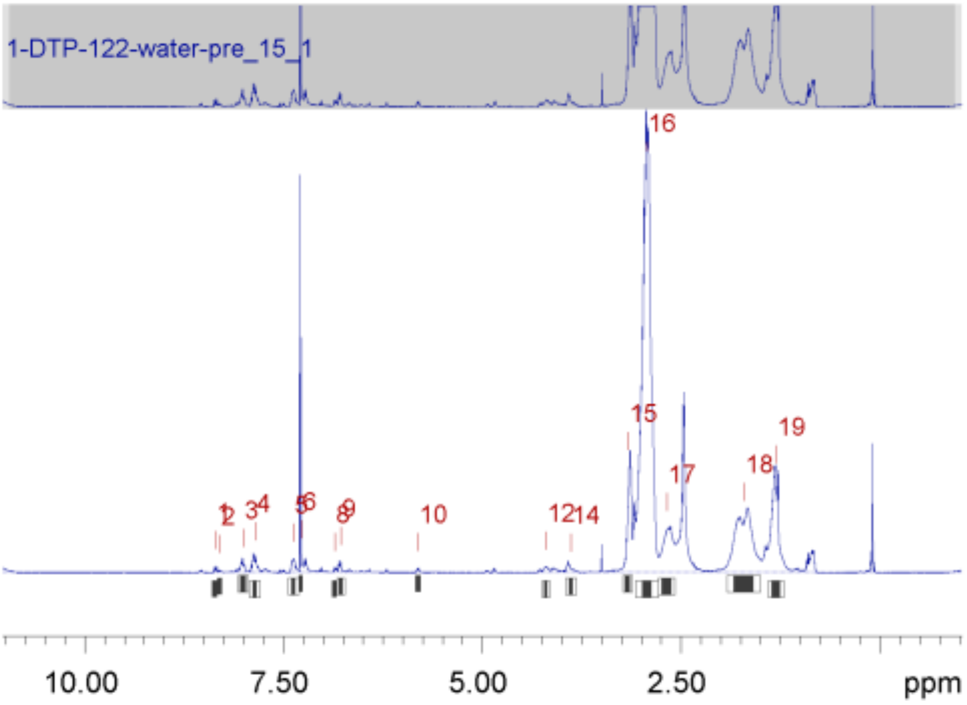

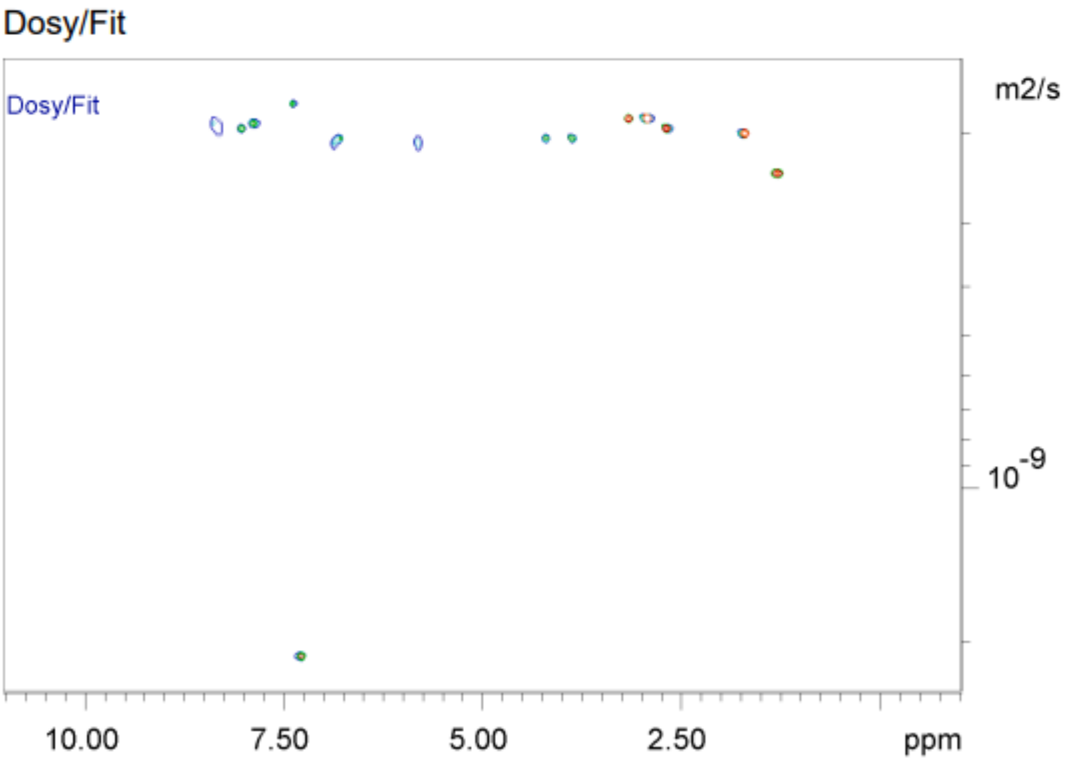

Fitted function:	f (x) = Io * exp (-D * x^2 * gamma^2 * littleDelta^2 (bigDelta-littleDelta/3)* 10^4
used gamma:	26752 rad/(s*Gauss)
used little delta:	0.0028000 s
used big delta:	0.099900 s
used gradient strength:	variable
Random error estimation of data:	RMS per spectrum (or trace/plane)
Systematic error estimation of data:	worst case per peak scenario
Fit parameter Error estimation method:	from fit using calculated y uncertainties
Confidence level:	95%
Used peaks:	peaks from C:/Data/Chemists/FedericaS/1-DTP-122-water-pre/15/pdata/1/peaklist1D.xml
Used integrals:	area integral
Used Gradient strength:	all values (including replicates) used

Peak name	F2 [ppm]	Io	error	D [m2/s]	error	fitInfo
1	8.357	2.01e+08	7.462e+05	1.91e-10	1.697e-12	Done
2	8.294	1.19e+08	6.688e+05	1.95e-10	2.620e-12	Done
3	8.004	1.05e+09	9.824e+05	1.94e-10	4.323e-13	Done
4	7.855	1.68e+09	1.056e+06	1.91e-10	2.878e-13	Done
5	7.371	1.33e+09	1.028e+06	1.75e-10	3.298e-13	Done
6	7.274	3.24e+09	1.127e+06	2.13e-09	1.775e-12	Done
8	6.851	2.30e+08	6.930e+05	2.08e-10	1.480e-12	Done
9	6.774	6.04e+08	8.891e+05	2.06e-10	7.177e-13	Done
10	5.802	1.83e+08	7.464e+05	2.07e-10	1.997e-12	Done
12	4.192	5.69e+08	8.767e+05	2.05e-10	7.497e-13	Done
14	3.881	9.20e+08	1.060e+06	2.05e-10	5.600e-13	Done
15	3.164	1.01e+10	9.550e+05	1.87e-10	4.276e-14	Done
16	2.926	9.31e+10	1.493e+06	1.89e-10	7.271e-15	Done
17	2.678	1.10e+10	1.310e+06	1.94e-10	5.510e-14	Done
18	1.707	2.49e+10	1.838e+06	1.98e-10	3.490e-14	Done
19	1.299	1.58e+10	1.289e+06	2.38e-10	4.513e-14	Done

Current fit display

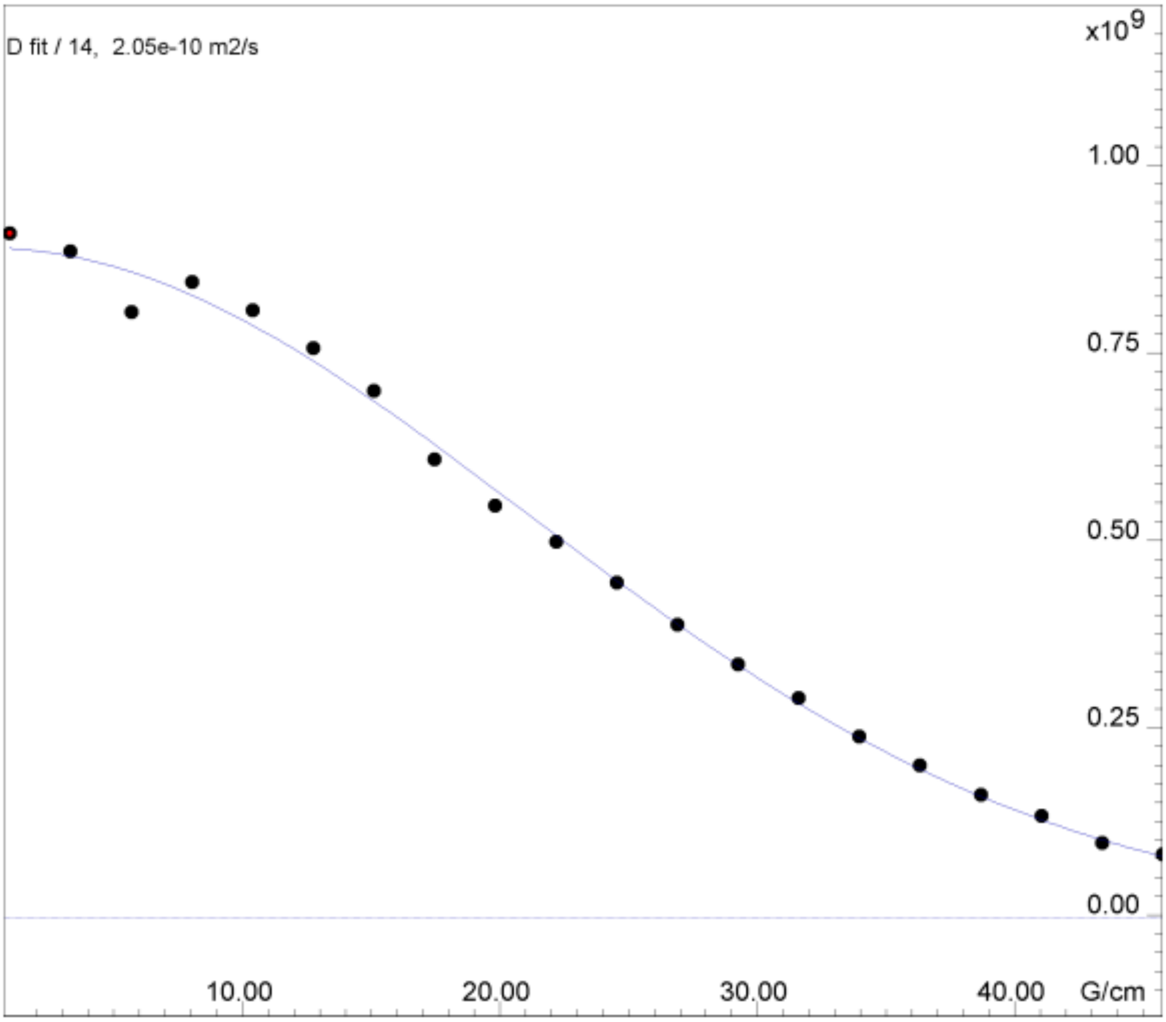

1. Gruendling, T.; Guilhaus, M.; Barner-Kowollik, C., Fast and Accurate Determination of Absolute Individual Molecular Weight Distributions from Mixtures of Polymers via Size Exclusion Chromatography−Electrospray Ionization Mass Spectrometry. *Macromolecules* **2009,** *42* (17), 6366-6374.
2. Fulmer, G. R.; Miller, A. J.; Sherden, N. H.; Gottlieb, H. E.; Nudelman, A.; Stoltz, B. M.; Bercaw, J. E.; Goldberg, K. I., NMR chemical shifts of trace impurities: common laboratory solvents, organics, and gases in deuterated solvents relevant to the organometallic chemist. *Organometallics* **2010,** *29* (9), 2176-2179.
3. Sokolova, A.; Christoforidis, J.; Eltobaji, A.; Barnes, J.; Darmann, F.; Whitten, A. E.; de Campo, L., BILBY: Time-of-Flight Small Angle Scattering Instrument. *Neutron News* **2016,** *27* (2), 9-13.
4. Sokolova, A.; Whitten, A. E.; de Campo, L.; Christoforidis, J.; Eltobaji, A.; Barnes, J.; Darmann, F.; Berry, A., Performance and characteristics of the BILBY time-of-flight small-angle neutron scattering instrument. *Journal of Applied Crystallography* **2019,** *52* (1), 1-12.
5. Arnold, O.; Bilheux, J.-C.; Borreguero, J.; Buts, A.; Campbell, S. I.; Chapon, L.; Doucet, M.; Draper, N.; Leal, R. F.; Gigg, M., Mantid—Data analysis and visualization package for neutron scattering and μ SR experiments. *Nuclear instruments and methods in physics research section a: accelerators, spectrometers, detectors and associated equipment* **2014,** *764*, 156-166.
6. Manalastas-Cantos, K.; Konarev, P. V.; Hajizadeh, N. R.; Kikhney, A. G.; Petoukhov, M. V.; Molodenskiy, D. S.; Panjkovich, A.; Mertens, H. D.; Gruzinov, A.; Borges, C., ATSAS 3.0: expanded functionality and new tools for small-angle scattering data analysis. *Journal of applied crystallography* **2021,** *54* (1), 343-355.
7. Do, P. T.; Poad, B. L.; Frisch, H., Programming Photodegradability into Vinylic Polymers via Radical Ring-Opening Polymerization. *Angewandte Chemie* **2023,** *135* (6), e202213511.
8. Huang, H.; Sun, B.; Huang, Y.; Niu, J., Radical Cascade-Triggered Controlled Ring-Opening Polymerization of Macrocyclic Monomers. *J Am Chem Soc* **2018,** *140* (33), 10402-10406.
9. Chambers, L. C.; Barner-Kowollik, C.; Barner, L.; Michalek, L.; Frisch, H., Photostationary State in Dynamic Covalent Networks. *ACS Macro Letters* **2022,** *11* (4), 532-536.
10. Foster, H.; Stenzel, M. H.; Chapman, R., PET-RAFT Enables Efficient and Automated Multiblock Star Synthesis. *Macromolecules* **2022,** *55* (14), 5938-5945.
11. Olson, R. A.; Levi, J. S.; Scheutz, G. M.; Lessard, J. J.; Figg, C. A.; Kamat, M. N.; Basso, K. B.; Sumerlin, B. S., Macromolecular Photocatalyst for Synthesis and Purification of Protein–Polymer Conjugates. *Macromolecules* **2021,** *54* (10), 4880-4888.
